# 2′-*O*-Methylation of the second transcribed nucleotide within the mRNA 5′ cap impacts the protein production level in a cell-specific manner and contributes to RNA immune evasion

**DOI:** 10.1093/nar/gkac722

**Published:** 2022-08-26

**Authors:** Karolina Drazkowska, Rafal Tomecki, Marcin Warminski, Natalia Baran, Dominik Cysewski, Anaïs Depaix, Renata Kasprzyk, Joanna Kowalska, Jacek Jemielity, Pawel J Sikorski

**Affiliations:** Centre of New Technologies, University of Warsaw, Banacha 2c, 02-097 Warsaw, Poland; Institute of Biochemistry and Biophysics, Polish Academy of Sciences, Pawinskiego 5a, 02-106 Warsaw, Poland; Institute of Genetics and Biotechnology, Faculty of Biology, University of Warsaw, Pawinskiego 5a, 02-106 Warsaw, Poland; Division of Biophysics, Institute of Experimental Physics, Faculty of Physics, University of Warsaw, Pasteura 5, 02-093 Warsaw, Poland; Centre of New Technologies, University of Warsaw, Banacha 2c, 02-097 Warsaw, Poland; Department of Environmental Microbiology and Biotechnology, Institute of Microbiology, Faculty of Biology, University of Warsaw, Miecznikowa 1, 02-096 Warsaw, Poland; Institute of Biochemistry and Biophysics, Polish Academy of Sciences, Pawinskiego 5a, 02-106 Warsaw, Poland; Clinical Research Centre, Medical University of Bialystok, M. Sklodowskiej-Curie 24a, 15-276 Bialystok, Poland; Centre of New Technologies, University of Warsaw, Banacha 2c, 02-097 Warsaw, Poland; Centre of New Technologies, University of Warsaw, Banacha 2c, 02-097 Warsaw, Poland; Division of Biophysics, Institute of Experimental Physics, Faculty of Physics, University of Warsaw, Pasteura 5, 02-093 Warsaw, Poland; Centre of New Technologies, University of Warsaw, Banacha 2c, 02-097 Warsaw, Poland; Centre of New Technologies, University of Warsaw, Banacha 2c, 02-097 Warsaw, Poland; Department of Environmental Microbiology and Biotechnology, Institute of Microbiology, Faculty of Biology, University of Warsaw, Miecznikowa 1, 02-096 Warsaw, Poland

## Abstract

In mammals, m^7^G-adjacent nucleotides undergo extensive modifications. Ribose of the first or first and second transcribed nucleotides can be subjected to 2′-*O*-methylation to form cap1 or cap2, respectively. When the first transcribed nucleotide is 2′-*O*-methylated adenosine, it can be additionally modified to *N*6,2′-*O*-dimethyladenosine (m^6^A_m_). Recently, the crucial role of cap1 in distinguishing between ‘self’ and ‘non-self’ in mammalian cells during viral infection was revealed. Here, we attempted to understand the impact of cap methylations on RNA-related processes. Therefore, we synthesized tetranucleotide cap analogues and used them for RNA capping during *in vitro* transcription. Using this tool, we found that 2′-*O*-methylation of the second transcribed nucleotide within the mRNA 5′ cap influences protein production levels in a cell-specific manner. This modification can strongly hamper protein biosynthesis or have no influence on protein production levels, depending on the cell line. Interestingly, 2′-*O*-methylation of the second transcribed nucleotide and the presence of m^6^A_m_ as the first transcribed nucleotide serve as determinants that define transcripts as ‘self’ and contribute to transcript escape from the host innate immune response. Additionally, cap methylation status does not influence transcript affinity towards translation initiation factor eIF4E or *in vitro* susceptibility to decapping by DCP2; however, we observe the resistance of cap2-RNA to DXO (decapping exoribonuclease)-mediated decapping and degradation.

## INTRODUCTION

A key feature of eukaryotic mRNA is the presence of a 5′ cap structure, which is indispensable for several biological processes such as pre-mRNA splicing, mRNA export and translation (1,2). The 5′ cap of mRNA consists of 7-methylguanosine (m^7^G) linked by a 5′,5′-triphosphate bridge to the first transcribed nucleotide. This m^7^GpppN modification, referred to as cap0, is formed enzymatically during transcription. Cap0 can be further modified by nuclear cap 2′-*O*-methyltransferase 1 (CMTR1) to form cap1 (m^7^GpppN_m_), wherein the ribose of the first transcribed nucleotide is methylated at the 2′-*O*-position (3). Transcripts bearing cap1 can be additionally subjected to ribose 2′-*O*-methylation at the second transcribed nucleotide. This reaction is catalysed by another methyltransferase, CMTR2, resulting in the formation of cap2 (m^7^GpppN_m_pN_m_) (4). Interestingly, studies have shown that CMTR2 can also act on substrates lacking 2′-*O*-methylation at the m^7^G-adjacent nucleotide *in vitro* (4), thereby forming an m^7^GpppNpN_m_ capping structure called cap2-1. In addition to 2′-*O*-methylation, when adenosine is the first transcribed nucleotide, it can be methylated at the *N*6-position of the nucleobase by phosphorylated CTD-interacting factor 1 (PCIF1) to form *N*6,2′-*O*-dimethyladenosine (m^6^A_m_) (5–7). Importantly, transcriptome-wide mass spectrometry (MS)-based studies have shown that in some mammalian cells, *N*6-methyladenosine (m^6^A) is as abundant as the first transcribed nucleotide as m^6^A_m_ (8). However, it is unclear whether PCIF1 can add an *N*6-methyl group to adenosine without prior ribose 2′-*O*-methylation or, conversely, if the presence of m^6^A within the cap is a consequence of the activity of an unknown demethylase.

For a long time, the role of extensive methylation of the mammalian cap structure was not characterized adequately. Recently, it was reported that 2′-*O*-methylation of the first transcribed nucleotide is important for the differentiation between ‘self’ and ‘non-self’ RNA during viral infection in mammalian cells (9–11). Among the sensors of viral RNAs, interferon (IFN)-induced proteins with tetratricopeptide repeats (IFITs) can be distinguished. IFIT1 competes with eukaryotic translation initiation factor 4E (eIF4E) for m^7^G-capped RNAs, and lack of methylation at the 2′-*O*-position of the first transcribed nucleotide favours IFIT1 binding (12,13). Cellular mRNAs are protected from IFIT1 sensing by the presence of cap1.

The 5′ cap is a crucial element in mRNA translation, which occurs in a cap-dependent manner. The eIF4F cap-binding complex, containing eIF4E cap-binding protein, promotes translation initiation when bound to the cap (1,2). The presence of cap0 guarantees efficient mRNA translation, and it was shown that the protein production level may be further elevated by cap1 in particular cell lines (14). Recently, we also found that exogenously delivered mRNAs carrying m^6^A as the first transcribed nucleotide show increased protein yields in some mammalian cell lines, but only in the context of cap1 (14). Intriguingly, studies by other authors published so far indicate that the presence of the *N*6-methyl group on adenosine as the m^7^G-adjacent nucleotide can increase (5,15), decrease (6,7,16) or not change (6) translation properties of mammalian mRNAs. However, the impact of 2′-*O*-methylation of the second transcribed nucleotide on translation remains elusive.

mRNA stability is largely controlled by the presence of the cap, as it protects transcripts from exonucleolytic degradation mediated by cellular RNases acting from the 5′ end (1,2,17). Enzymes of the XRN1 family are unable to degrade capped mRNAs. Independent of the type of cap modification tested to date (i.e. cap0 and cap1), transcripts are prone to the activity of DCP2, a major cellular decapping enzyme (14), which, in cooperation with its cellular partner DCP1, releases m^7^GDP and the monophosphorylated RNA chain (17). Another protein, DXO (decapping exoribonuclease), serves as a quality control factor to eliminate aberrantly capped transcripts and acts as a decapping enzyme. However, in contrast to DCP2, DXO removes the entire cap structure by hydrolysing the phosphodiester bond between the first and second transcribed nucleotide, resulting in the release of m^7^GpppN and monophosphorylated RNA, and continues to degrade RNA as an exoribonuclease in the 5′–3′ direction (18). Interestingly, DXO decapping activity was shown to be inhibited by cap1 2′-*O*-methylation (19), suggesting its potential role in distinguishing ‘self’ from ‘non-self’ RNAs. The influence of 2′-*O*-methylation of the second transcribed nucleotide on decapping by DCP2 or DXO is yet to be investigated.

One of the experimental approaches to study the impact of modifications within the mRNA 5′ cap on mRNA-related processes is the preparation of *in vitro* transcribed mRNA (IVT mRNA) and analysis of how the introduced modifications change the biological properties of the transcript. Until recently, IVT mRNA bearing methylations of m^7^G-adjacent nucleotides were prepared through the use of appropriate methyltransferases (3,4,7,15). However, this method is time-consuming and loabour-intensive, requiring the use of recombinant methyltransferases, preparation of IVT mRNA with an unmodified cap, modifying the cap structure with purified enzymes and analysis of cap modification efficiency after each attempt. Recently, we and other researchers have used a novel approach to obtain IVT mRNA bearing modifications at the first transcribed nucleotide introduced co-transcriptionally, i.e. the use of trinucleotide cap analogues as initiators of *in vitro* transcription (14,20). However, trinucleotide cap analogues enable the preparation of transcripts only with modifications of the first transcribed nucleotide. Therefore, to generate capped IVT mRNA that is methylated at the second transcribed nucleotide, i.e. a transcript with cap2 or cap2-1, we generated new molecular tools—tetranucleotide cap analogues chemically decorated with 2′-*O*-methylation at the first and second transcribed nucleotides (cap2) or exclusively at the second transcribed nucleotide (cap2-1)—and used these for efficient co-transcriptional RNA capping. These new tools, together with previously synthesized trinucleotide cap analogues (14), enabled us to comprehensively study the impact of cap methylation status, i.e. 2′-*O*-methylation of the first and second transcribed nucleotides, as well as *N*6-nucleobase methylation of adenosine as the first transcribed nucleotide, on protein production levels in mammalian cells grown under optimal or stress conditions. The results showed that 2′-*O*-methylation of the second transcribed nucleotide regulates protein biosynthesis in a cell-dependent manner. To understand this phenomenon, we characterized the interactomes of transcripts with differently methylated cap structures in mammalian cells originating from different tissues. Moreover, we observed the resistance of RNA capped with cap2 to DXO-mediated decapping and degradation, and preserved vulnerability to DCP2 action. We also showed that 2′-*O*-methylation of the second transcribed nucleotide and *N*6-methylation of adenosine as the first transcribed nucleotide serve as determinants defining transcripts as ‘self’ and contribute to transcript immune evasion.

## MATERIALS AND METHODS

### Synthesis of cap analogues

Tetranucleotide cap2 (m^7^GpppN_m_pG_m_pG) and cap2-1 (m^7^GpppNpG_m_pG) analogues were synthesized according to the procedure described earlier (14). Briefly, trinucleotide 5′-phosphates pN_m_pG_m_pG and pNpG_m_pG were synthesized by the phosphoramidite method on a high-loaded solid support (DMT-2′-*O*-TBDMS-rG^iBu^ 3′ lcaa PrimerSupport 5G, 308 μmol/g) using ÄKTA Oligopilot plus 10 synthesizer (GE Healthcare) or in a syringe, respectively. When using the manual method, the support was loaded directly into a syringe and the reagents and solvents were flowed through the support. Additionally, when the coupling steps were performed, the syringe containing the support and phosphoramidite solutions was shaken in a thermomixer for ∼20 min. In the coupling steps, 2 equivalents (eqv) of the phosphoramidite (G_m_^iBu^/A_m_^Bz^/m^6^A_m_^Pac^), 2′-OH phosphoramidite (A^Bz^/m^6^A^Ac^) or biscyanoethyl phosphoramidite and 0.30 M 5-(benzylthio)-1-H-tetrazole in acetonitrile (except for m^6^A^Ac^ dissolved in dichloromethane/acetonitrile 2/1) were recirculated through the column for 15 min. A solution of 3% (v/v) dichloroacetic acid in toluene was used as a detrytilation reagent and 0.05 M iodine in pyridine for oxidation. After the last cycle of synthesis, 20% (v/v) diethylamine in acetonitrile was passed through the column to remove 2-cyanoethyl protecting groups. Finally, the solid support was washed with acetonitrile and dried with argon. The product was cleaved from the solid support and deprotected with AMA (methylamine/ammonium hydroxide 1:1 v/v; 50°C, 1 h), evaporated to dryness and re-dissolved in dimethylsulphoxide (DMSO; 200 μl). The TBDMS groups were removed using triethylammonium trihydrofluoride (TEA·3HF; 250 μl, 65°C, 3 h), and then the mixture was cooled down and diluted with 0.25 M NaHCO_3_(aq) (20 ml). The product was isolated by ion-exchange chromatography on DEAE Sephadex [gradient elution 0–1.2 M tetraethylammonium bromide (TEAB)] to afford—after evaporation—the triethylammonium salt of pN_m_pG_m_pG and pNpG_m_pG trinucleotides. The scales, yields, and high resolution mass spectrometry (HRMS) data for particular trinucleotides are summarized in Table 1.

**Table 1.** List of the synthesized pNpG_m_pG trinucleotides

Sequence	Synthesis scale (μmol)	Yield (μmol)	*m/z*_calculated_	*m/z*_found_
pA_m_pG_m_pG	50	29.60	1064.18198	1064.18216
p(m^6^A_m_)pG_m_pG	25	13.30	1078.19763	1078.19870
pApG_m_pG	100	63.80	1050.16633	1050.16740
p(m^6^A)pG_m_pG	150	116.00	1064.18198	1064.18339

The triethylammonium salt of pN_m_pG_m_pG was dissolved in DMSO to 0.05 M, and the P-imidazolide of *N*7-methylguanosine 5′-diphosphate (m^7^GDP-Im) (21) (2 eqv) and anhydrous ZnCl_2_ (20 eqv) were added. The mixture was stirred for ∼48 h and the reaction was quenched by addition of 10 volumes of aqueous solution of EDTA (20 mg/ml) and NaHCO_3_ (10 mg/ml). The product was isolated by ion-exchange chromatography on DEAE Sephadex (gradient elution 0–1.2 M TEAB) and purified by semi-preparative reverse phase high performance liquid chromatography (RP-HPLC; gradient elution 0–15% acetonitrile in 0.05 M ammonium acetate buffer pH 5.9) to afford—after evaporation and repeated freeze-drying from water—the ammonium salt of tetranucleotide cap m^7^GpppNpG_m_pG.

For cap2-1 derivatives, P-imidazolide activation was performed on trinucleotides. The triethylammonium salt of pNpG_m_pG was dissolved in DMSO (0.08 M) followed by the addition of imidazole (16 eqv), 2,2′-dithiodipyridine (6 eqv), triethylamine (3 eqv) and triphenylphosphine (6 eqv). The reaction was stirred at room temperature, until a complete conversion was found by RP-HPLC analysis. The reaction was quenched by the addition of a cold solution of sodium perchlorate (3 eqv) in acetone (10× V_reaction_). The resulting precipitate was washed with acetone, centrifuged until the supernatant was clear and dried under vacuum, affording the expected product as a white powder. The resulting P-imidazolide was directly used in a coupling reaction as described for pN_m_pG_m_pG with m^7^GDP (1.5 eqv) instead of m^7^GDP-Im. The final semi-preparative RP-HPLC was performed using a gradient elution from 0 to 50% acetonitrile in 120 min or 60 min for m^7^GpppApG_m_pG and m^7^Gppp(m^6^A)pG_m_pG, respectively. The reagent amounts, yields, and HRMS data for particular cap analogues are summarized in Table 2.

**Table 2.** List of the synthesized m^7^GpppNpG_m_pG tetranucleotide cap analogues

Sequence	Im-pNpG_m_pG (μmol)	m^7^GDP (μmol)	ZnCl_2_ (mmol)	m^7^GpppNpG_m_pG (μmol)	Yield	*m/z*_calculated_	*m/z*_found_
m^7^GpppA_m_pG_m_pG	8.50	17.0	170	4.74	56%	1503.21139	1503.21153
m^7^Gppp(m^6^A_m_)pG_m_pG	13.30	26.5	265	8.39	63%	1517.22704	1517.22838
m^7^GpppApG_m_pG	55	82.5	1.10	16	29%	1489.19574	1489.19661
m^7^Gppp(m^6^A)pG_m_pG	94.6	141.9	1.89	23.5	25%	1503.21139	1503.21179

### RNA preparation

#### Short RNA

Short RNAs were obtained as described in (14) with some modifications. Annealed IVTshF and IVTshR oligonucleotides, containing the T7 promoter sequence and an additional 35 nucleotides including the recognition site for DNAzyme, served as a template for *in vitro* transcription reactions. Reactions were set in 80 μl mixtures which contained RNA Pol buffer [40 mM Tris–HCl pH 7.9, 10 mM MgCl_2_, 1 mM dithiothreitol (DTT), 2 mM spermidine]; 1.25 μM annealed oligonucleotides; ATP, CTP, UTP, 3 mM each, and 0.75 mM GTP (R0481, Thermo Fisher Scientific); 2.25 mM of appropriate cap analogue (trinucleotide cap analogue for RNA capping with cap0 or cap1 or tetranucleotide analogue for capping with cap2 or cap2-1); 2 U/μl RiboLock RNase inhibitor (EO0381, Thermo Fisher Scientific); and 4 μl of home-made T7 RNA polymerase, and were incubated at 37°C. After 2 h, an additional 4 μl of home-made T7 RNA polymerase were added and reactions were conducted for another 2 h at 37°C; next, DNase I (EN0521, Thermo Fisher Scientific) was added (0.1 U/μl) for 30 min incubation at 37°C. The control sample—non-capped short RNA—was prepared in a reaction with ATP, CTP, UTP and GTP, 3 mM each, without any cap analogue. Transcripts were purified utilizing the RNA Clean & Concentrator-25 kit (R1017, Zymo Research). RNAs of the intended size were recovered from particular fractions collected during HPLC purification. For purification, a Phenomenex Clarity® 3 μm Oligo-RP column was utilized and a linear gradient of buffer B (0.1 M triethylammonium acetate pH 7.0 and 50% acetonitrile) from 10% to 26.7% in buffer A (0.1 M triethylammonium acetate pH 7.0) over 25 min at 1 ml/min was applied. Fractions containing both uncapped and capped transcripts were collected. RNAs after HPLC were recovered by isopropanol precipitation. To generate homogenous 3′ ends in these, transcripts were incubated at 1 μM concentration with 1 μM DNAzyme in 50 mM MgCl_2_ and 50 mM Tris–HCl pH 8.0 at 37°C for 1 h and were purified utilizing an RNA Clean & Concentrator-25 kit and again HPLC-purified, when fractions containing uncapped and capped transcripts, devoid of DNAzyme, were collected. RNAs after isopropanol precipitation and resuspension in water were utilized for enzymatic assays and for examination of capping efficiency, which was analysed on a 15% polyacrylamide/7 M urea/TBE gel.

Crude short transcripts after clean-up were used for preparation of RNAs utilized for affinity purification. Biotinylated pAp (pAp *N*6-PEG-biot) (22) was ligated to RNAs by T4 RNA ligase 1 (M0204, New England Biolabs). Reactions were set up with 10 μM RNAs, 400 μM pAp analogue, 1 mM ATP, 10% (v/v) DMSO and 10% (v/v) ligase in 1× commercial ligase buffer overnight at 16°C. RNAs after ligation were HPLC-purified and fractions containing transcripts with ligated biotinylated pAp analogue were collected. Precipitated and re-suspended RNAs were treated with calf intestinal alkaline phosphatase (18009027, Thermo Fisher Scientific) to remove any triphosphates from the 5′ ends of uncapped molecules, which would interact with IFIT proteins later in the experiments. The enzyme was heat-inactivated (15 min at 65°C).

#### mRNAs

mRNAs encoding *Gaussia* luciferase were obtained as previously described in (14) with modifications. pJET-based construct, enabling preparation of polyadenylated [containing 36 nt-long poly(A) tail] mRNA, linearized with AarI enzyme served as a template for *in vitro* transcription reactions. Reactions were set up in 20 μl mixtures which contained RNA Pol buffer (40 mM Tris–HCl pH 7.9, 10 mM MgCl_2_, 1 mM DTT, 2 mM spermidine); 40 ng/μl of DNA template; ATP, CTP, UTP, 2 mM each, and 0.5 mM GTP (R0481, Thermo Fisher Scientific); 1.5 mM of appropriate cap analogue (trinucleotide cap analogue for RNA capping with cap0 or cap1 or tetranucleotide analogue for capping with cap2 or cap2-1); 2 U/μl RiboLock RNase inhibitor (EO0381, Thermo Fisher Scientific) and 1 μl of home-made T7 RNA polymerase, and were incubated at 37°C. After 2 h, an additional 1 μl of home-made T7 RNA polymerase was added and the reaction was conducted for another 2 h at 37°C; next, DNase I (EN0521, Thermo Fisher Scientific) was added (0.1 U/μl) for 30 min incubation at 37°C. Control sample—non-capped mRNA—was prepared in reaction with ATP, CTP, UTP and GTP, 2 mM each, and no cap analogue added. The crude mRNAs were purified using NucleoSpin RNA Clean-up XS (740903, Macherey-Nagel). mRNAs were HPLC-purified using an RNASep Prep RNA Purification Column (ADS Biotec) at 55°C, a linear gradient of buffer B (0.1 M triethylammonium acetate pH 7.0 and 50% acetonitrile) from 17.5% to 25.8% in buffer A (0.1 M triethylammonium acetate pH 7.0) over 20 min and a flow rate of 0.9 ml/min. mRNAs from collected fractions were recovered by isopropanol precipitation. Non-capped mRNAs from samples with capped mRNAs were removed by two separate treatments, i.e. with RNA 5′ polyphosphatase (RP8092H, Epicentre) and Xrn1 (M0338, New England Biolabs), including purification using a NucleoSpin RNA Clean-up XS step in between. Transcripts after enzymatic reactions were purified again utilizing NucleoSpin RNA Clean-up XS.

### Cell lines and culture conditions

A549 human carcinoma lung cells (ATCC CCL-185) and 3T3-L1 murine embryonic fibroblasts (ATCC CL-173), human embryo kidney (HEK) 293 Flp-In T-REx cell line (R78007, Thermo Fisher Scientific) and stable cell lines obtained in this background (generated as described below) were cultured in Dulbecco’s modified Eagle’s medium (DMEM; Gibco) supplemented with 10% foetal bovine serum (FBS), GlutaMAX (Gibco) and penicillin*/*streptomycin. THP-1 human monocytes (ATCC TIB-202) and the murine immature dendritic cell line JAWS II (ATCC CRL-11904) were grown in RPMI 1640 (Gibco) supplemented with 10% FBS, sodium pyruvate (Gibco), penicillin*/*streptomycin and, in the case of JAWS II cells, additionally with 5 ng*/*ml granulocyte–macrophage colony-stimulating factor (GM-CSF; PeproTech). Cells were cultured at 37°C in a 5% CO_2_ atmosphere.

#### Doxycycline induction

To induce tet promoter-regulated expression in Flp-In T-REx cell lines, cells were cultured with addition of 100 ng/ml doxycycline (D9891, Sigma-Aldrich) during the experiments and for 5–7 days before (with splitting in the meantime).

#### Stress conditions

To stress the cells, incubation in medium with addition of Universal Type I Interferon (IFNα) (11200-1, PBL Assay Science) was performed. For quantitative reverse transcription—PCR (RT–qPCR) assessment of cellular stress phenotypes upon IFNα treatment, A549 cells were seeded in 96-well plates (10 000 cells/well), 24 h before addition of IFNα for 5 h at 0, 50, 500 or 5000 U/ml concentration. Cells for protein production studies were seeded 5 h before mRNA transfection and treated with 0, 50, 500 or 5000 U/μl IFNα during seeding; after transfection, cells were cultured without exchanging medium for an additional 72 h. For affinity purification and subsequent western blot analysis, A549 cells were cultured in a 100 mm dish to 90–95% confluency and incubated with 0 or 500 U/μl of IFNα for 5 h; JAWS cells were cultured in T-75 flasks and incubated with 0 or 5000 U/μl of IFNα for 5 h.

### Stable cell lines

The HEK 293 Flp-In T-REx (R78007, Thermo Fisher Scientific) cell line was utilized to obtain stable cell lines expressing short hairpin microRNAs (shmiRs) upon tetracycline/doxycycline induction. Cell lines with shmiR for CMTR1, shmiR for CMTR2 and shmiRs for both CMTR1 and CMTR2 were generated. pKK-RNAi-nucCHERRYmiR-EGFP-TEV (a gift from Andrzej Dziembowski and Roman Szczesny, Addgene plasmid #105810) (23) was used as the negative control (sh neg) cell line. To obtain cell lines with shmiRs for CMTRs, modified pKK-RNAi-nucCHERRYmiR-EGFP-TEV plasmids were utilized. In each stable cell line, the bidirectional inducible promoter regulates both mCherry and EGFP (enhanced green fluorescent protein) reporter protein expression. shmiR sequences were introduced downstream of the fluorescent protein coding sequence (CDS) as described below.

#### shmiR cloning

The scheme of the cloning strategy is presented in Supplementary Figure S1A. shmiRs were designed as in (24,25) (sequences presented in Supplementary Figure S1B, C) and synthesized by Thermo Fisher Scientific GeneArt service. pKK-RNAi-nucCHERRYmiR-EGFP-TEV was digested with AfeI and NotI, and extracted from the gel. Plasmid devoid of the negative shmiR insert was treated with Klenow fragment (EP0051, Thermo Fisher Scientific) for blunting, and circularized by T4 DNA ligase. The obtained vector and insert coding for shmiR for CMTR1 flanked with sequences for BshTI and NheI enzymes were treated with the BshTI and NheI restriction endonuclease pair. Digestion products were then ligated to generate the sh CMTR1 construct. The sh CMTR2 construct was generated by ligation of pKK-RNAi-nucCHERRYmiR-EGFP-TEV digested with BspTI and NotI, and the insert coding for the shmiR for CMTR2 (flanked with BspTI and NotI sites) was cut out with the same enzyme pair. The sh CMTR2 construct was treated with BshTI and NheI enzymes and ligated with the insert coding for shmiR for CMTR1 with matching sticky ends to generate the sh CMTR1 + 2 construct.

#### HEK 293 Flp-In T-REx stable cell line generation

HEK 293 Flp-In T-REx cells were seeded in a 6-well plate at 500 000 cells/well. Next day, the cells were transfected with 1.8 ng of pOG44 plasmid encoding the Flp recombinase (V600520, Thermo Fisher Scientific) and 200 ng of sh CMTR1, sh CMTR2, sh CMTR1 + 2 or pKK-RNAi-nucCHERRYmiR-EGFP-TEV (negative control, sh neg) via lipofection with the use of Lipofectamine 3000 (L3000, Thermo Fisher Scientific). Five hours later, the medium with the transfection mixture was discarded and replaced with a fresh portion of complete DMEM. After 2 days, cells were trypsinized and transferred to 60 mm plates with a selection medium containing 50 μg/ml hygromycin B (10687010, Gibco). The selection medium was replaced every few days. After ∼2 weeks, single cells with integrated constructs formed colonies which were detached with trypsin and transferred to a multi-well plate. Hygromycin selection was continued while further amplifying cells from each cell line. Integration of constructs was validated by microscopic analysis, where fluorescent proteins were detected. Efficiency of CMTR silencing upon doxycycline induction was analysed by western blot.

#### Microscopic analysis

Stable cell lines and non-transfected parental HEK 293 Flp-In T-REx cells as a control were seeded on poly-l-lysine (P4707, Sigma-Aldrich)-coated coverslips in 24-well plates at 50 000 cells/well, with or without doxycycline, 24 h before fixation. On the day of imaging, cells were washed with phosphate-buffered saline (PBS) and fixed using 4% paraformaldehyde in PBS for 10 min at room temperature. After three washes with PBS, coverslips were mounted on slides using Fluoromount Aqueous Mounting Medium (F4680, Sigma-Aldrich) supplemented with 1 μg/ml 4′,6-diamidino-2-phenylindole (DAPI). Cells were imaged with a Zeiss AXIO Observer.Z1 inverted fluorescence microscope, using a ×40/0.6 objective. DAPI, EGFP and mCherry emissions were detected at emission wavelengths of 410–440, 499–529 and 659–759 nm, respectively, after excitation at 370–400 nm for DAPI, 450–488 nm for EGFP and 505–605 nm for mCherry. Data were analysed using ZEN Lite software (Carl Zeiss).

#### Western blot analysis of CMTR depletion

Lysates from induced and non-induced stable shmiR cell lines were prepared utilizing Cell Culture Lysis 5× Reagent (E1531, Promega) and mixed with Laemmli sample buffer [63 mM Tris–HCl (pH 6.8), 10% glycerol, 2% sodium dodecylsulphate (SDS), 0.01% bromophenol blue and 5% 2-mercaptoethanol] and heated for 5 min at 95°C. Samples were separated in a pre-cast 4–20% TGX Stain-Free polyacrylamide gel (456-8093, Bio-Rad). A PageRuler Prestained Protein Ladder, 10–180 kDa (26616, Thermo Fisher Scientific) was used as a molecular weight marker during electrophoresis. Proteins were transferred onto the nitrocellulose membrane (GE10600002, GE Healthcare) using the Trans-Blot Turbo Transfer System (Bio-Rad). Proteins on the membrane were stained with Ponceau S buffer (0.5% Ponceau S, 1% acetic acid) followed by 1 h blocking in 5% skim milk in PBST (PBS with 0.1% Tween-20). The membrane was cut horizontally into two pieces and each of them was incubated with the appropriate primary antibody in PBST to detect CMTR2 (HPA048265, Sigma-Aldrich) diluted 500-fold and glyceraldehyde phosphate dehydrogenase (GAPDH; NB300-327, Novus Biologicals) diluted 5000-fold, overnight at 4°C. After washing with PBST, membranes were incubated with secondary horseradish peroxidase (HRP)-conjugated goat anti-rabbit antibody (32260, Thermo Fisher Scientific) diluted 10 000-fold in PBST for 1 h at room temperature. Detection was performed with the use of Immobilon Western Chemiluminescent HRP Substrate (WBKLS0, Merck) in an Amersham Imager 600. Following CMTR2 detection, the membrane was stripped using mild stripping buffer (200 mM glycine, 0.1% SDS, 1% Tween-20, pH 2.2) and re-probed with antibody recognizing CMTR1 (HPA029980, Sigma-Aldrich) diluted 1000-fold.

### Reporter mRNA protein production in cells: luminescence assay

For protein biosynthesis studies, 10 000 A549, JAWS II, 3T3-L1 or THP-1 cells were seeded on the day of the experiment in 100 μl of medium/well of 96-well plates, with a particular concentration of IFNα, if applicable. HEK 293 Flp-In T-REx cells were seeded a day before transfection (15 000 cells/well), in 100 μl of medium/well of 96-well plates, with or without doxycycline. Cells in each well were transfected with a mixture containing 5 ng of HPLC-purified mRNA in 5 μl of Opti-MEM (51985026, Gibco) mixed with 0.3 μl Lipofectamine MessengerMAX Transfection Reagent (LMRNA, Invitrogen) in an additional 5 μl of Opti-MEM. Three wells for each mRNA sample served as technical replicates. After 72 h incubation, the medium was collected for examination of secreted luciferase activity. For detection of *Gaussia* luciferase activity, 50 μl of 10 ng/ml h-coelenterazine (301, NanoLight) in PBS was added to 10 μl of cell culture medium and the luminescence was measured with the use of a Synergy H1 (BioTek) microplate reader. Total protein production for each mRNA over 3 days (cumulative luminescence) was reported as a mean value ± SEM. Data were collected for three biological replicates.

### Affinity purification with MS and western blot analysis

#### Affinity purification

For affinity purification/MS (AP-MS) analysis, A549 cells were cultured to 90–95% confluency; two 100 mm culture dishes were used for each replicate. All subsequent steps were carried on ice with pre-cooled reagents, if not stated otherwise. PBS-washed cells were detached with the use of a scraper in 800 μl of lysis buffer (20 mM Tris–HCl pH 7.4, 150 mM NaCl, 2 mM MgCl_2_, 2 mM DTT, 0.2% IGEPAL CA-630 and a cocktail of protease inhibitors) per dish. Cells and buffer from each dish were collected and transferred to one Eppendorf-type tube, and this mixture was aspirated into a syringe and passed through a 26G needle seven times. Next, lysates were centrifuged at 4°C, 10 000 × *g*, for 10 min. Lysates from both plates were pooled and then aliquoted. Each of five portions was mixed with freshly denatured RNA (or lysis buffer as a control). During lysate centrifugation, short capped and biotinylated RNAs (10 pmol of each molecule in 10 μl of water) were heated at 95°C for 3 min and cooled down on ice to denature them. Then, to each sample, 10 μl of 2× concentrated lysis buffer was added. RNAs with lysates were incubated at 4°C for 1 h with rotation. Then, samples were mixed with pre-washed streptavidin magnetic beads, 40 μl of 50% slurry per sample (CMG-227, PerkinElmer) and incubated at 4°C for 30 min with rotation. Pre-washing of beads comprised three washes with lysis buffer (without protease inhibitors) and two washes with wash buffer (20 mM Tris–HCl pH 7.4, 150 mM NaCl, 2 mM MgCl_2_, 2 mM DTT, 0.2% Tween-20). After incubation with lysates, beads were washed twice with lysis buffer and twice with wash buffer. After discarding the buffer from the last wash, beads were frozen until the next steps for MS analysis were performed. For the experiment with JAWS II, lysate was prepared from ∼2 × 10^6^ cells. The suspension fraction of JAWS II was collected by centrifuging medium, while adherent cells were detached with the use of trypsin. Cells were pooled, washed thoroughly with PBS and transferred to two Eppendorf-type tubes. Cells from each of the two pellets were suspended in 800 μl of lysis buffer and the next steps of the procedure were the same as described above.

For affinity purification/western blot analysis, A549 cells were cultured to 90–95% confluency, and the lysate from one 100 mm culture dish was prepared for each condition (–/+IFNα). For JAWS II cells, lysates were prepared from ∼6 × 10^6^ cells for each condition. Subsequent steps were carried as described above, but 2 pmol of each short capped and biotinylated RNA molecule and 8 μl of bead slurry, respectively, were used per sample.

#### Mass spectrometry

To each sample, 20 μl of 100 mM NH_4_HCO_3_ and 2.5 μl of 200 mM TCEP [tris(2-carboxyethyl)phosphine] were added; samples were vortexed and placed in a horizontal shaker (10 000 rpm) at room temperature for 30 min. Subsequently, 2 μl of MMTS (methyl methanethiosulphonate) were added and samples were shaken for 20 min at room temperature. Trypsin/LysC (V5071, Promega) was solved in 8 M urea in 100 mM NH_4_HCO_3_, to a final enzyme concentration of 0.02 μg/μl; 50 μl was added to each sample. Samples were incubated with shaking at 37°C for 4 h, then 300 μl of NH_4_HCO_3_ was added and the digestion was performed overnight. Samples were acidified with 10 μl of 5% trifluoroacetic acid (TFA). The resulting peptide mixture was purified on Oasis HLB 96-well plates containing 10 mg of sorbent per plate (186000128, Waters), vacuum-dried and suspended in 60 μl of 2% acetonitrile, 0.1% TFA. Samples were measured in an online LC-MS set-up of EvosepOne (Evosep Biosystems) coupled to an Orbitrap Exploris 480 Thermo Fisher Scientific mass spectrometer.

Peptide mixtures were loaded on Evotips C18 trap columns, according to the manufacturer’s protocol: activation of sorbent with 0.1% formic acid (FA) in acetonitrile, 2 min incubation in 1-propanol and chromatographic sorbent equilibration with 0.1% FA in water. Samples were loaded in 30 μl of 0.1% FA after each step, EvoTips were centrifuged at 600 × *g* for 1 min. Chromatographic separation was carried out at a flow rate of 500 nl/min using the 44 min (30 samples per day) performance gradient on an EV1106 analytical column (Dr. Maisch C18 AQ, 1.9 μm beads, 150 μm ID, 15 cm long, Evosep Biosystems, Odense, Denmark). Data were acquired in positive mode with a data-dependent method using the following parameters: the MS1 resolution was set to 60 000 with a normalized AGC target of 300%, auto maximum injection time and a scan range of 350–1400 *m/z*. For MS2, the resolution was set to 15 000 with a standard normalized AGC target, auto maximum injection time, and the top 40 precursors within an isolation window of 1.6 *m/z* were considered for MS/MS analysis. Dynamic exclusion was set at 20 s with an allowed mass tolerance of ± 10 ppm and a precursor intensity threshold of 5 × 1000. Precursors were fragmented in HCD mode with a normalized collision energy of 30%. The spray voltage was set to 2.1 kV, with a funnel RF level of 40 and heated capillary temperature of 275°C.

Raw data were analysed with PEAKS Studio 10.6 64bit Bioinfor (26) and searched against Uniprot human (78 120 entries, for the A549 samples) or mouse (55 360 entries for the JAWS II samples) reference proteomes. Fixed modifications: methylthio (MMTS) at cysteines; variable: oxidation methionine, acetyl n-term. MS error and 0.1 Da, MS/MS level 0.2 Da, false discovery rate (FDR) 1%, digestion: trypsin semi-specific, max variable PTM per peptide: 3. Protein level analysis was performed using the ‘Label Free’ PEAKS module.

Each group consisted of three biological replicates; the average signal intensity was calculated for every condition. Data were analysed in such a way as to indicate which proteins co-precipitate with the immobilized decoy in a repeatable and specific manner compared with the given control group.

The ratio of any protein identified and quantified was calculated in relation to the average level in the mock-treatred samples normalized to ‘1.0’. Heat maps, clustering and group correlation were done in Perseus software (27). Ratio values were represented as log2 in heat maps; rows and columns were clustered based on Euclidean distance calculation.

#### Western blot analysis

Beads after protein affinity purification and input samples (cell lysates) were mixed with Laemmli sample buffer and heated for 5 min at 95°C. Samples were separated by 10% SDS–polyacrylamide gel electrophoresis (PAGE). Then, proteins were transferred onto a nitrocellulose membrane using the Trans-Blot Turbo Transfer System (Bio-Rad). Proteins on the membrane were stained with Ponceau S buffer, and 1 h blocking in 5% skim milk in PBST was performed. Membranes were cut horizontally into three pieces and each was incubated with the appropriate primary antibody diluted 1000-fold in PBST to detect human eIF4E (2067, Cell Signaling), IFIT1 (PA3-848, Thermo Fisher Scientific) and NCBP1 (PA5-83607, Thermo Fisher Scientific) or murine counterparts overnight at 4°C. After washing with PBST, membranes were incubated with secondary goat anti-rabbit (HRP) antibody diluted 10 000-fold in PBST for 1 h at room temperature. Detection was performed with the use of Immobilon Western Chemiluminescent HRP Substrate in an Amersham Imager 600.

### eIF4E affinity assay: microscale thermophoresis (MST)

To determine the dissociation constant for eIF4E protein and capped short RNA complexes, an MST-based method was applied, previously described in (28), with modification of the utilized ligand form; here, capped short RNAs were analysed. Binding assay mixtures were prepared in 20 μl as follows: 10 nM fluorescent probe m^7^Gp_5_OC_3_(5)FAM, 50 nM murine eIF4E and ligand (differently capped short RNAs ranging from 1.75 μM to 0.05 nM) in an MST buffer (50 mM HEPES–KOH pH 7.2, 100 mM KCl, 0.5 mM EDTA, 0.05% Tween-20). Samples after preparation, without any additional incubation, were loaded into Monolith NT.115 Capillaries (MO-KO22; NanoTemper Technologies). MST was performed using a Monolith NT.115 instrument (NanoTemper Technologies) at 25°C. Instrument parameters were adjusted to 100% LED Blue power and Medium MST power. To determine the *K_D_*_,app_ values, a standard 1:1 binding model was fitted to the data using PALMIST software (version 1.4.4). Confidence intervals were determined using error-surface projection (ESP) (29).

### Recombinant human DCP2 (hDCP2) and hDXO cloning, overproduction and purification

#### hDXO open reading frame (ORF) cloning

cDNA obtained from total RNA isolated from HEF 293 Flp-In T-REx cells was used as a template to amplify the ORF coding for the full-length human DXO wild type by PCR with the primer pair hDXO1For–hDXO1Rev (0.2 μM both) and using Phusion High-Fidelity DNA Polymerase (F530, Thermo Fisher Scientific) with 1× HF buffer, 0.2 mM dNTP mix and 3% DMSO. The PCR product corresponding to the hDXO wild-type ORF was gel-purified using the Gel-Out kit (023-50, A&A Biotechnology) and inserted by SLIC into BamHI/XhoI sites of the pET28M N-6xHis-TEV vector (30), giving rise to the phDXOwt construct. *Escherichia coli* strain MH1 (*araD lacX74 galU hsdR hsdM rpsL*) was used for transformation with SLIC products. An insert encompassing the ORF encoding hDXO mut (E234A D236A) was obtained in a two-step amplification. In the first step, two PCRs were performed as above, but using hDXOfor–hDXOmutR and hDXOmutF–hDXOrev primer pairs and phDXOwt as a template. The two PCR products were gel-purified as above and their mixture was used in the second round of amplification. Initially, 10 cycles of PCR product joining in the absence of primers was performed, followed by addition of the hDXOfor–hDXOrev primer pair and normal PCR (as for hDXO wild type). Finally, the resulting PCR product corresponding to the hDXO mut ORF was purified and cloned into pET28M N-6xHis-TEV vector as described above, giving rise to the phDXOmut construct. Oligonucleotides and plasmids used in the study are listed in Supplementary Table S1 and S2, respectively.

#### hDCP2 cloning

The 1–350 amino acid-coding region of hDCP2(E147Q, E148Q) was PCR amplified from the pET28a-hDCP2(E147Q, E148Q) plasmid [a gift from Megerditch Kiledjian (Addgene plasmid #72215) (31)] with the primer pair hDCP2F and hDCP2R and using Phusion High-Fidelity DNA Polymerase with 1× HF buffer and 0.2 mM dNTP mix. The PCR product was purified using NucleoSpin Gel and PCR Clean-up (740609, Macherey-Nagel), and was cloned into pJET1.2/blunt vector using the CloneJET PCR Cloning Kit (K1231, Thermo Fisher Scientific). Chemocompetent *E. coli* TOP10 bacteria were transformed with ligation mixture. The selected plasmid containing the 1–350 amino acid-coding region of hDCP2(E147Q, E148Q) of interest was cut with BamHI and NotI, and the insert containing the hDCP2 fragment was ligated, utilizing the same sites, into pET28b vector, giving rise to phDCP2(1-350)mut. phDCP2(1-350)mut was obtained as a counterpart for plasmid coding for the wild-type 1–350 amino acid region of the hDCP2 protein, herein named phDCP2(1-350)wt, kindly gifted by Megerditch Kiledjian.

#### Recombinant protein production and purification

The *E. coli* BL21-CodonPlus(DE3)-RIL strain (Agilent; *E. coli* B F^–^*ompT hsdS*[r_B_^–^ m_B_^–^] *dcm*^+^ Tet^r^*gal λ[DE3] endA* Hte [*argU ileY leuW* Cam^r^]) was transformed with either phDXOwt, phDXOmut, phDCP2(1-350)wt or phDCP2(1-350)mut. Transformants were grown in a standard Luria–Broth (LB) medium supplemented with 50 μg/ml kanamycin and 34 μg/ml chloramphenicol. Subsequently, 1 l of Auto Induction Medium (AIM) Super Broth Base including trace elements (AIMSB02, Formedium) containing 2% glycerol and both antibiotics was inoculated with 30 ml of the starter culture. Bacteria were grown for 48 h at 18°C with shaking (150 rpm) and eventually collected by centrifugation at 4500 rpm in a Sorvall H6000A/HBB6 swinging-bucket rotor for 15 min at 4°C.

The bacterial pellet was resuspended in 70 ml of lysis buffer [50 mM Tris–HCl pH 8.0, 200 mM NaCl, 10 mM imidazole, 10 mM 2-mercaptoethanol, 1 mM phenylmethylsulphonyl fluoride (PMSF), 0.02 μM pepstatin A, 0.02 μg/ml chymostatin, 0.006 μM leupeptin, 20 μM benzamidine hydrochloride], incubated with lysozyme (50 μg/ml; Roth) for 30 min in a cold cabinet and then broken up in an EmulsiFlex-C3 High Pressure homogenizer at 1500 bar. The homogenate was centrifuged in a Sorvall WX Ultra Series ultracentrifuge (F37L rotor) at 33 000 rpm for 45 min at 4°C.

The extract (supernatant after high-speed ultracentrifugation) was used for protein purification using the ÄKTA Xpress system (GE Healthcare), employing nickel affinity chromatography on an ÄKTA-compatible 5 ml column that was manually filled with Ni-NTA Superflow resin (Qiagen). The column was equilibrated with 25 ml of low-salt (LS) buffer (50 mM Tris–HCl pH 7.4, 200 mM NaCl, 10 mM imidazole, 10 mM 2-mercaptoethanol) prior to extract loading. After protein binding, the resin was sequentially washed with 40 ml of LS buffer, 25 ml of high-salt (HS) buffer (50 mM Tris–HCl pH 7.4, 1 M NaCl, 10 mM imidazole, 10 mM 2-mercaptoethanol) and again 20 ml of LS buffer. Bound proteins were recovered by elution with 30 ml of buffer E (50 mM Tris–HCl pH 7.4, 200 mM NaCl, 300 mM imidazole). Pooled eluate fractions (∼5 ml) were dialysed overnight at 4°C against 2 l of LS buffer in the presence of 100/50 μg of home-made TEV/SUMO protease (hDXO wt and mut/hDCP2 wt and mut). This mixture was afterwards subjected to a second round of purification on the nickel resin, performed using the ÄKTA Purifier system (GE Healthcare) and employing LS buffer for collection of the flow-through, containing the protein of interest devoid of the tag, and buffer E2 (50 mM Tris–HCl pH 8.0, 1 M NaCl, 300 mM imidazole) for elution of 6×His-tagged SUMO or TEV protease and the cleaved-off 6×His-SUMOTag or 6×His-TEV sequence. For hDXO variants, further purification from contaminating chaperones and degradation products was achieved by separation of pooled flow-through obtained in the second round of affinity chromatography on a size exclusion Superdex 75 10/300 GL column (GE Healthcare) using 1.2 column volumes of gel filtration (GF) buffer (20 mM Tris–HCl pH 8.0, 150 mM NaCl). Two fractions corresponding to the maximum of *A*_280_ nm absorbance were collected after gel filtration and pooled together. Solutions containing purified recombinant were mixed with glycerol (30% v/v), aliquoted, snap-frozen in liquid nitrogen and stored at –80°C until needed for biochemical activity assays. Proteins were inspected by 10% SDS–PAGE stained with Coomassie Brilliant Blue R-250. A PageRuler Prestained Protein Ladder, 10–180 kDa was used as a molecular weight marker during electrophoresis.

### RNA decapping assays

#### hDCP2

Purified short uncapped pppRNA (25 nt) and differently capped RNAs with their uncapped fractions (27 nt + 25 nt, respectively) were utilized as substrates in hDCP2 enzymatic assay. Reactions were set up in 25 μl with 1.6 U/μl of RiboLock in the reaction buffer (50 mM Tris–HCl pH 8.0, 50 mM NH_4_Cl, 0.01% NP-40, 5 mM MgCl_2_, 2 mM MnCl_2_, 1 mM DTT, where both DTT and MnCl_2_ were always added shortly before setting up the reaction) at 37°C. hDCP2 (wt or mut) at a concentration of 70 nM was tested; substrates were utilized at a concentration of 120 nM. Reactions without enzyme served as controls. During assays, 5 μl of reaction mixtures were collected at each time point (0, 5, 15, 30 and 60 min), and reactions were stopped by mixing with formamide loading dye (for PAGE) containing 20 mM EDTA and snap-frozen in liquid nitrogen. Samples were separated on denaturing 15% polyacrylamide/7 M urea/TBE gels. After staining with SYBR Gold Nucleic Acid Gel Stain (S11494, Thermo Fisher Scientific), gels were scanned in a Typhoon FLA9500 Imager (GE Healthcare).

#### hDXO

Purified short uncapped pppRNA (25 nt) and differently capped RNAs with its uncapped fractions (27 nt + 25 nt, respectively) were utilized as substrates in hDXO enzymatic assay. Reactions were set up in 25 μl in reaction buffer [10 mM Tris–HCl pH 7.9, 50 mM KOAc, 2 mM Mg(OAc)_2_, 2 mM MnCl_2,_ 1 mM DTT, where both DTT and MnCl_2_ were always added shortly before setting up the reaction] at 37°C. Concentrations of 0.6 μM (wt or mut) and 1.5 μM hDXO (wt) were tested; substrates were utilized at a concentration of 70 nM. Reactions without enzyme served as controls. Assays and sample analysis were conducted as for those with hDCP2 protein, described above.

### RT–qPCR

Cells were incubated for 5 h with various concentrations of IFNα and then lysed utilizing the SingleShot Cell Lysis Kit (1725080, Bio-Rad). Lysates were used for reverse transcription reactions with oligo(dT_20_) primer and M-MLV Reverse Transcriptase (28025, Invitrogen). cDNAs generated by reverse transcription served as templates for quantitative PCRs (qPCRs) performed utilizing SsoAdvanced Universal SYBR Green Supermix (1725271, Bio-Rad) with primer pairs for amplification of *IFIT1, IFIT2, IFIT3, RIG-I, MDA5, PKR, OAS1* and *GAPDH*; the latter was employed as a reference gene. The sequences of all used primers are shown in Supplementary Table S1.

## RESULTS

### Tetranucleotide cap analogues enable efficient co-transcriptional capping of RNA with cap2 and cap2-1 structures *in vitro*

**Figure 1.**
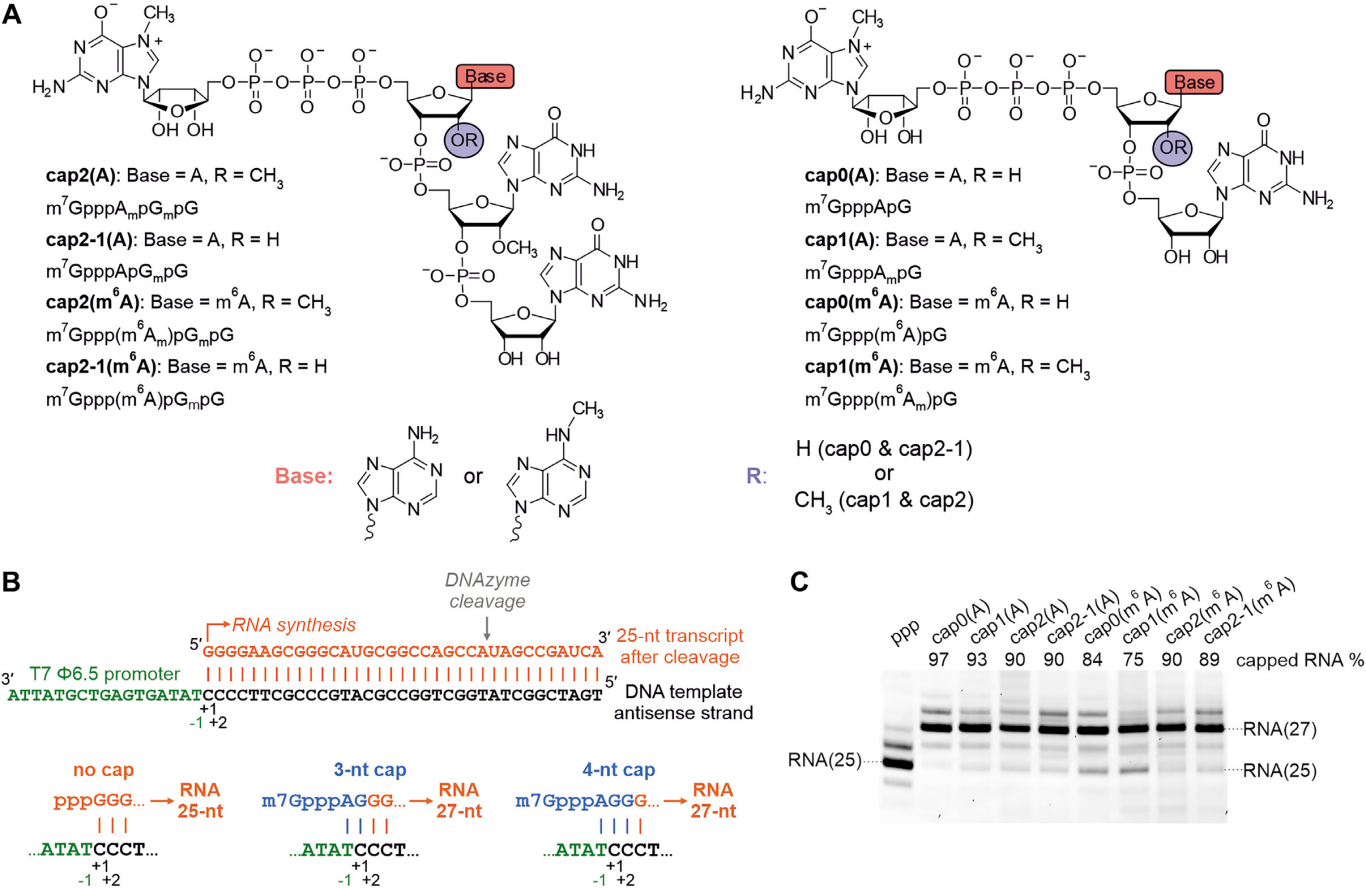
Tetranucleotide cap analogues act as initiators of *in vitro* transcription reactions. (**A**) Structure of the cap analogues used in this study; newly synthesized tetranucleotide and previously obtained trinucleotide (14) cap analogues are presented on the left and right side, respectively. (**B**) Comparison of the major transcription initiation events during the *in vitro* transcription reaction when either no cap analogue, a trinucleotide cap analogue or a tetranucleotide cap analogue was used as an initiator. Capped RNA obtained in the *in vitro* transcription reaction with a tri- or tetranucleotide cap analogue is 27 nt long, as uncapped RNA is 25 nt long. (**C**) Analysis of short RNAs obtained by *in vitro* transcription using T7 RNA polymerase in the presence of different cap analogues (DNAzyme-trimmed and HPLC-purified transcripts). The capping efficiency values (percentage) determined by densitometric quantification of the major bands corresponding to capped and uncapped RNA are shown at the top of the gel. Minor extra bands most probably arise from unspecific addition of nucleotides during *in vitro* transcription.

To investigate the influence of 2′-*O*-methylation of the second transcribed nucleotide on the biological and biochemical properties of RNA, we required a convenient tool to generate capped RNAs with this modification. Similar to previously described trinucleotide cap analogues utilized for generating IVT mRNA efficiently capped with cap0 or cap1 (14), we chemically synthesized tetranucleotide cap analogues bearing ribose 2′-*O*-methylation at the first and second transcribed nucleotide m^7^GpppN_m_pN_m_, i.e. cap2, or only at the second nucleotide m^7^GpppNpN_m_, i.e. cap2-1. The first transcribed nucleotide was adenosine [cap2(A) and cap2-1(A)] or adenosine methylated within the nucleobase at the *N*6-position [cap2(m^6^A) and cap2-1(m^6^A)] (Figure 1A). By combining these two compounds with the previously synthesized analogue of cap1, we created a set of different tri- and tetranucleotide cap analogues combining all possible cap methylation statuses (cap0, cap1, cap2 and cap2-1) with A and m^6^A (8,32,33). Tetranucleotide cap analogues were obtained via a combination of solid-phase and solution chemistry. Highly loaded solid supports generated the trinucleotide 5′-phosphates pN_(__m)_pG_m_pG, which were conjugated with the P-imidazolide of *N*7-methylguanosine 5′-diphosphate (m^7^GDP-Im). The product was purified using ion-exchange chromatography followed by RP-HPLC. The purity and identity of the compounds obtained were confirmed by RP-HPLC and HRMS, respectively (HPLC profiles and HRMS spectra are provided in Supplementary Table S3).

Next, we investigated the efficiency of incorporation of the new tetranucleotide analogues into RNA during *in vitro* transcription compared with the previously tested trinucleotide set. Annealed oligo DNA with a Φ 6.5 promoter sequence followed by 35 nucleotides served as a template for the reaction with T7 RNA polymerase, in which the concentration of the cap analogues was 3-fold higher than that of GTP. The transcripts obtained were HPLC-purified, treated with DNAzyme to trim transcripts at the 3′ end and separated on a 15% denaturing polyacrylamide gel. Trimmed uncapped transcripts were 25 nt long, whereas the respective RNAs capped with either tri- or tetranucleotide analogues were 27 nt long (including m^7^G), as expected based on the T7 promoter sequence (Figure 1B). We observed a very high capping efficiency for the new tetranucleotide analogues at 90% for cap2(A), cap2-1(A) and cap2(m^6^A), and slightly less for cap2-1(m^6^A) (89%). The highest percentage of capped RNA was achieved in the presence of cap0(A) and cap1(A) trinucleotides (97% and 93%, respectively) (Figure 1C). Trinucleotides with *N*6-methylated adenosine were incorporated with slightly reduced efficiencies at 84% and 75% for cap0(m^6^A) and cap1(m^6^A), respectively (Figure 1C), as observed previously (14).

### Differences in cap methylation status affect protein production in a cell-specific manner

**Figure 2.**
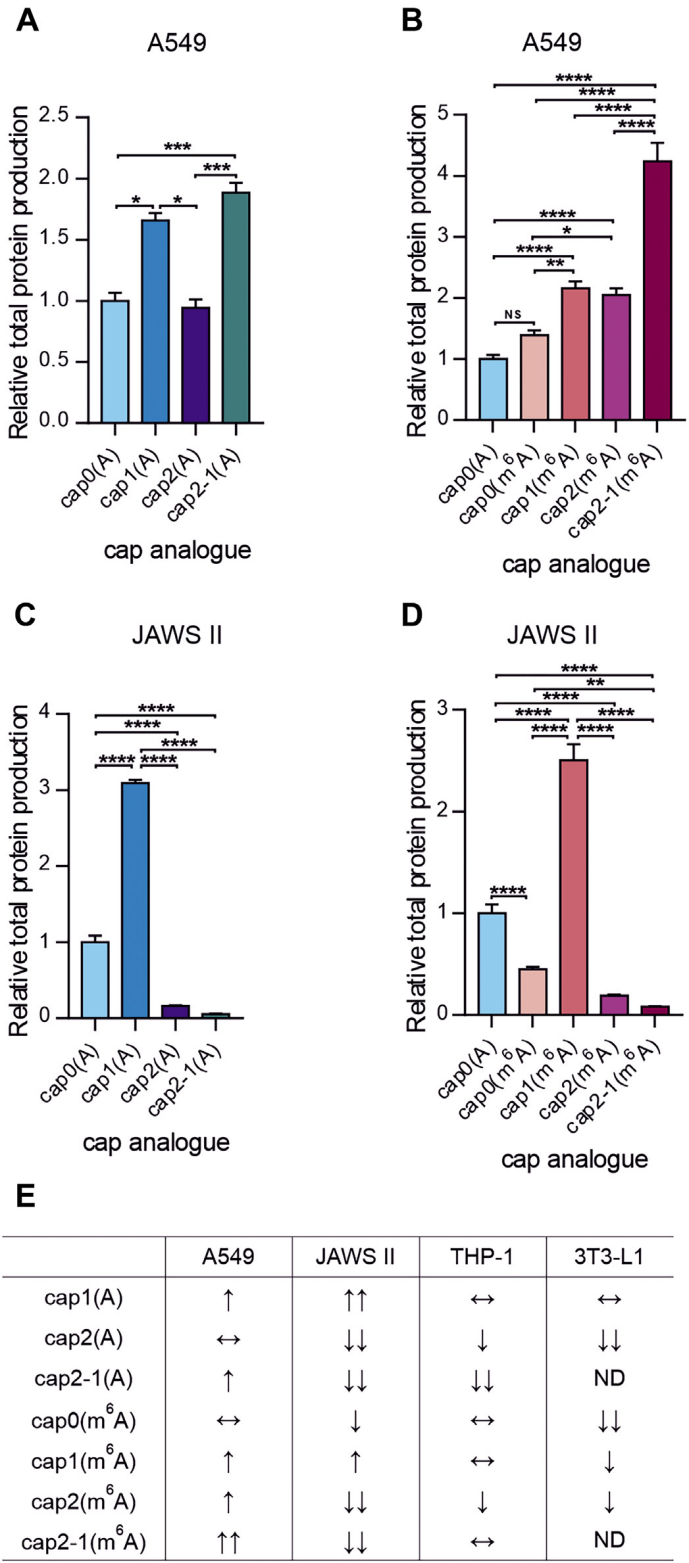
Protein production levels are affected by cap methylation status. Relative total protein production after 72 h measured in the medium from culture of (**A** and **B**) A549 and (**C** and **D**) JAWS II cells transfected with IVT mRNAs encoding *Gaussia* luciferase bearing various cap analogues at their 5′ ends. Bars represent the mean value ± SEM normalized to transcripts with cap0(A). Statistical significance: NS, not significant; *P <0.05; **P <0.01; ***P <0.001; ****P <0.0001 (one-way ANOVA with Turkey's multiple comparisons test). (Raw data from three independent biological replicates are shown in Supplementary Figure S2; each independent biological replicate consisted of three independent transfections.) (**E**) Summary table presenting the influence of the cap methylation status on protein production levels in A549, JAWS II, THP-1 and 3T3-L1 cells relative to transcript with cap0(A), ↑, increase of protein production; ↓, decrease of protein production; double arrows indicate a large (at least 3-fold) increase/decrease; ↔, no significant change.

Given that new tetranucleotide cap analogues are efficiently incorporated into RNA during *in vitro* transcription reactions, we studied the impact of 2′-*O*-methylation status within the cap structure on protein production levels in mammalian cells. We prepared IVT mRNAs encoding *Gaussia* luciferase capped with differently methylated cap analogues and performed luminescence assays. However, before IVT mRNA can be used for cell transfection, it should be of sufficient purity. Double-stranded RNA impurities, which together with the uncapped version of the studied mRNA could unspecifically interfere with the cell immune system, were of special concern to us (14,34,35). Therefore, all capped IVT mRNAs were purified by RP-HPLC, followed by enzymatic removal of uncapped mRNAs by treatment with 5′ polyphosphatase and Xrn1 (14). Mammalian cells from the human lung carcinoma A549 cell line and murine immature dendritic JAWS II cells were transfected with the purified IVT mRNAs. In A549 cells, cap methylation status had a moderate effect on protein production (Figure 2A, B). Notably, protein production levels were not affected by the identity of the first transcribed nucleotide (A versus m^6^A) in the context of cap0 (Figure 2B). In contrast, the presence of 2′-*O*-methylation at the first transcribed nucleotide modestly increased the total protein production for IVT mRNAs bearing cap1 relative to transcripts containing respective cap0 versions (Figure 2A, B). Introduction of the 2′-*O*-methyl group only at the second transcribed nucleotide [cap2-1(A)] had a similar effect on protein biosynthesis to the presence of 2′-*O*-methylation at the first transcribed nucleotide [cap1(A)] (Figure 2A). Interestingly, protein biosynthesis was 2- and 3-fold higher for mRNA bearing cap2-1(m^6^A) relative to transcripts with cap1(m^6^A) or cap0(m^6^A), respectively (Figure 2B). Surprisingly, the combination of both 2′-*O*-methylations led to a decrease in protein production compared with mRNAs with the respective cap2-1 versions, such that protein production from mRNA with cap2(A) dropped to the level observed for transcripts with cap0(A) (Figure 2A). Additionally, the luminescence measured for cells transfected with mRNA bearing cap2(m^6^A) was almost at the same level as for cells transfected with transcripts bearing cap1(m^6^A) (Figure 2B).

Interestingly, differences in cap methylation status had a greater impact on protein production levels in JAWS II than in A549 cells (Figure 2A–D). As expected, the presence of the 2′-*O*-methyl group at the first transcribed nucleotide significantly increased protein production in murine immature dendritic cells (14) (Figure 2C, D). mRNAs with cap1(A) provided three times more protein than transcripts with the respective cap0 version (Figure 2C), whereas transcripts with cap1(m^6^A) provided 5.5 times higher luminescence compared with the protein level produced from mRNA with cap0(m^6^A) (Figure 2D). In contrast to the results from A549 cells, the presence of *N*6-methyladenosine as the first transcribed nucleotide in JAWS II cells decreased the protein production level 2-fold in the context of cap0 (Figure 2B, D). The introduction of a methyl group at the second transcribed nucleotide had the opposite effect on protein production in JAWS II relative to A549 cells. Protein biosynthesis from mRNA with cap2-1(A) and cap2-1(m^6^A) was 56 and 31 times lower compared with the amount of protein produced from transcripts with the cap1 versions (Figure 2C, D). The presence of both 2′-*O*-methyl groups within the cap structure modestly improved the protein production level from IVT mRNAs compared with transcripts with cap2-1; however, these changes were not statistically significant.

Given that differences in cap methylation status shape protein production levels in A549 and JAWS II cells, we evaluated the protein biosynthesis from differently capped mRNAs and whether these were characteristic of the two cell lines or a general phenomenon due to the presence of a 2′-*O*-methyl group at m^7^G-adjacent nucleotides. We also analysed whether *N*6-methylation of the adenosine nucleobase influenced protein production levels in a cell-specific manner. To examine the impact of cap methylation on protein biosynthesis, we extended our analyses to two more cell lines from different tissues of origin, namely human THP-1 monocytic cells and murine 3T3-L1 embryonic fibroblasts (Supplementary Figures S3 and S4). In the two tested cell lines, the presence of *N*6-methyladenosine as the first transcribed nucleotide in the context of cap0 reduced protein production in 3T3-L1 cells alone. Moreover, 2′-*O*-methylation of adenosine as the first transcribed nucleotide did not influence protein production compared with the protein level obtained for mRNA with cap0(A), whereas the same modification in the context of *N*6-methyladenosine as an m^7^G-adjacent nucleotide increased the protein production level in 3T3-L1 cells alone. The addition of a second 2′-*O*-methyl group within the cap structure (cap2) decreased protein production, regardless of the identity of the first transcribed nucleotide (A versus m^6^A) in both cell lines (Supplementary Figures S3 and S4). The only exception was the production of *Gaussia* protein from mRNA with cap2(m^6^A) in 3T3-L1 cells, where protein levels did not change compared with protein production from mRNA with cap0(m^6^A) (Supplementary Figure S4). 2′-*O*-Methylation of the second transcribed nucleotide alone (cap2-1) influenced protein production levels differently in THP-1 cells depending on the identity of the first transcribed nucleotide (A versus m^6^A). Protein production from mRNA bearing cap2-1(m^6^A) did not change, but was severely impaired for transcripts with cap2-1(A) compared with that from IVT mRNA bearing cap0 analogues (Supplementary Figure S3). The change in protein production levels caused by differences in cap methylation for the four tested cell lines is summarized in Figure 2E.

To gain insight into why differences in the cap methylation status of reporter mRNA affect protein production in a cell-specific manner, we attempted to identify proteins that interacted with 5′-capped RNAs in mammalian cells. We were particularly interested in understanding how cap structure methylation influences the early stage of protein biosynthesis because m^7^G is indispensable for cap-dependent translation initiation. Moreover, it is widely assumed that translation regulation depends primarily on the rate-limiting initiation step (36). To identify proteins that bind to differently capped transcripts, we employed AP-MS (Supplementary Figure S5). RNAs with different cap structures were biotinylated using a pAp–biotin conjugate (22). Cell lysates were subsequently incubated with the prepared RNAs, and ribonucleoprotein (RNP) complexes formed on capped transcripts were purified using streptavidin beads. MS/MS followed by label-free quantification revealed protein interactors for differently capped RNAs. In this analysis, we focused on two cell lines, A549 and JAWS II, for which the effect of cap methylation on the protein production level was the most extreme (Figure 2E). Moreover, we studied the influence of only cap 2′-*O*-methylation on protein binding to capped transcripts, as it had the greatest impact on protein biosynthesis (Figure 2E).

In the course of affinity purification experiments for A549 and JAWS II cell lines, we identified, respectively, 248 and 247 proteins that were more abundant than in the control group (mock) for at least one cap variant (Supplementary Datasets S1 and S2). The data were analysed to reveal proteins that co-purified with capped transcripts in a recurrent and specific manner compared wiht the control group. Next, the ratio of an identified protein relative to its average level in the mock samples normalized to ‘1.0’ was determined (Supplementary Figure S6). It was unclear which proteins among those identified as bound to RNA with 2′-*O*-methylated cap structures were responsible for modulating protein production levels in JAWS II cells. Therefore, we analysed the correlation between protein abundance for differently capped RNAs in both cell lines (Supplementary Figure S7). Regardless of the 2′-*O*-methylation status, the subset of proteins bound to RNA was quite similar in A549 cells; the Pearson correlation coefficient ranged from 0.670 [cap2(A) versus cap2-1(A)] to 0.906 [cap0(A) versus cap1(A)]. However, the composition of RNA–protein interactomes largely depended on the cap 2′-*O*-methylation status in JAWS II cells, and the Pearson correlation coefficient ranged from 0.026 [cap0(A) versus cap2-1(A)] to 0.665 [cap2(A) versus cap2-1(A)]. Interestingly, capped RNA interactomes from JAWS II cells formed two subgroups: (i) interactomes for transcripts bearing an unmethylated cap [cap0(A)] or 2′-*O*-methylated first transcribed nucleotide [cap1(A)]; and (ii) interactomes identified for capped RNA with a 2′-*O*-methylated second transcribed nucleotide [cap2(A) and cap2-1(A)]. Interactomes in each subgroup were more similar to each other than to any of the interactomes in the other subgroup.

### The affinity of capped RNA for the translation machinery is not affected by the 5′ end methylation status

**Figure 3.**
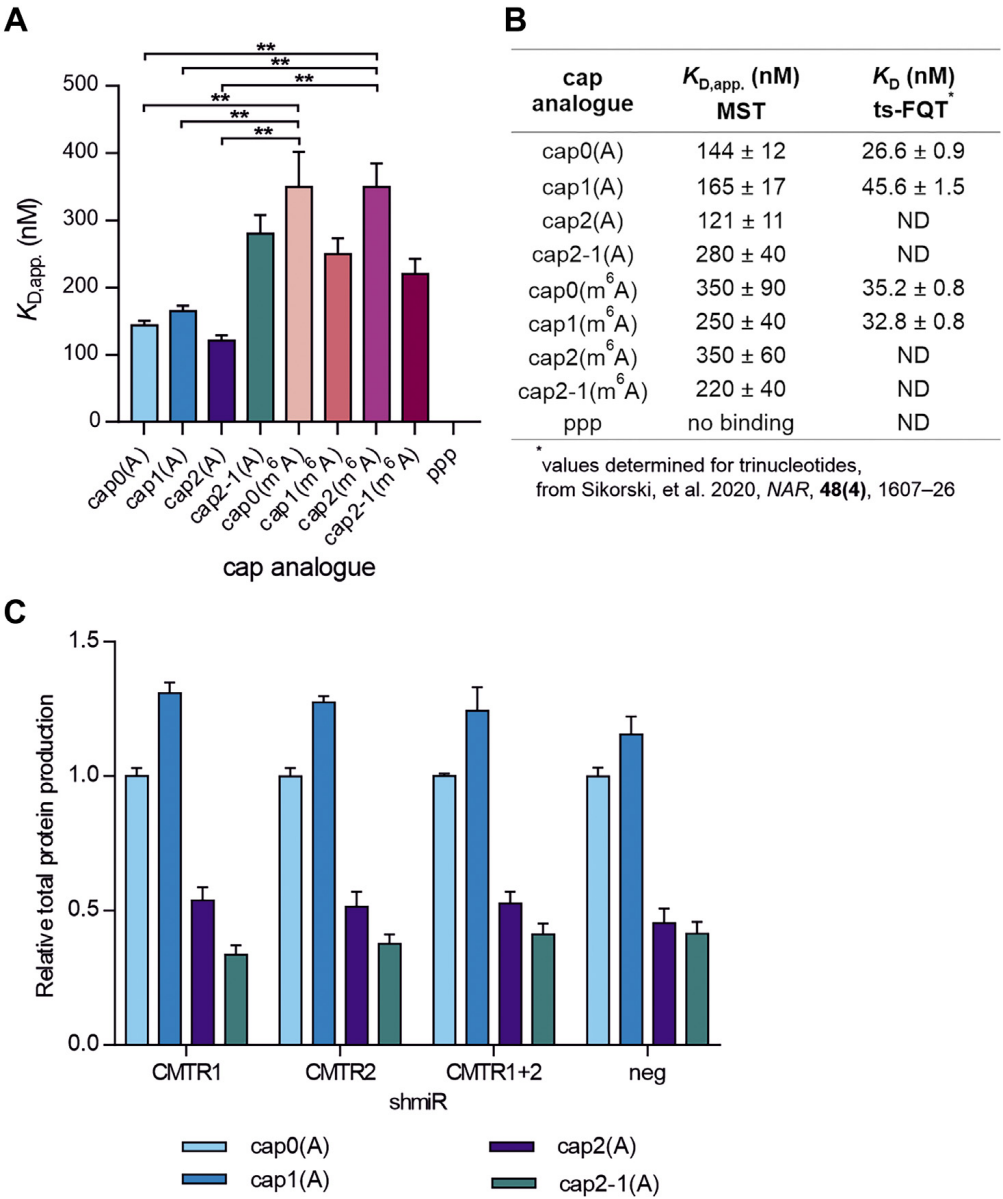
2′-*O*-Methylation within the cap structure does not influence RNA affinity for the translational machinery. (**A**) Relative affinities of transcripts bearing different cap analogues for murine eIF4E determined using MST. Bars represent the mean value ± SEM from three independent replicates. Statistical significance: **P <0.01 (one-way ANOVA with Turkey's multiple comparisons test). (Representative MST curves and competitive binding curves obtained in the experiment are presented in Supplementary Figure S8.) (**B**) Comparison of apparent binding constant values *K*_D,app_ for capped RNAs in complexes with murine eIF4E measured with MST with dissociation constants of eIF4E–cap complexes obtained using time-synchronized fluorescence quenching titration (ts-FQT) (14). For all RNAs, three independent replicates were performed, besides cap2(A) and cap2-1(A) RNAs, for which data from two replicates are presented. (**C**) Competition of differently capped IVT mRNAs encoding *Gaussia* luciferase with endogenous mRNAs for the translation machinery. HEK 293 Flp-In T-REx cells expressing shmiRs targeting CMTR1, CMTR2 or both (a negative control was utilized in parallel) were transfected with IVT mRNAs, and medium was collected after 72 h for luciferase activity analysis. Bars represent the mean value ± SEM normalized to *Gaussia* luciferase activity measured for transcripts with cap0(A). Data for three independent experiments are presented (each biological replicate consisted of three transfections).

To gain a deeper understanding of the influence of 2′-*O*-methylation of the cap structure on protein biosynthesis, we evaluated the binding affinities of differently capped RNAs for the translation initiation factor eIF4E. We used the recently developed MST assay for measuring temperature-induced changes in fluorescence for fluorescein amidite-labelled m^7^Gp_5_OC_3_(5)FAM upon competitive displacement from eIF4E by the capped transcripts (28). The MST assay confirmed previous results identifying the dissociation constants of eIF4E and trinucleotide cap analogues (14). The presence of 2′-*O*-methylation at adenosine as the first transcribed nucleotide did not influence RNA affinity for eIF4E (Figure 3A, B). We observed a similar effect for the methylation of the second transcribed nucleotide, regardless of the 2′-*O*-methylation status of the m^7^G-adjacent nucleotide. Interestingly, we observed a tendency for the capped RNA–eIF4E complex to be slightly destabilized in the presence of m^6^A as the first transcribed nucleotide, regardless of the cap 2′-*O*-methylation status, except for transcripts with cap2-1 (Figure 3A, B).

Next, to validate the biochemical data, we investigated the competition for the translation machinery between exogenously delivered reporter transcripts and endogenous mRNAs. We were interested in studying whether depriving endogenous mRNAs of cap 2′-*O*-methylation(s) would result in an increase in protein production levels for any of the studied IVT mRNAs. To this end, we down-regulated the expression of genes encoding CMTR1, CMTR2 or both, utilizing stable cell lines originating from the HEK 293 Flp-In T-REx cell line, in which we expressed particular shmiRs in an inducible manner (verification of stable cell lines with microscopic analysis and western blot is presented in Supplementary Figure S9). CMTR1 and CMTR2 are methyltransferases responsible for 2′-*O*-methylation of the first and second transcribed nucleotides, respectively (3,4). Differentially capped mRNAs encoding *Gaussia* luciferase were introduced via lipofection into cells cultured in the presence of doxycycline, expressing shmiRs for down-regulation of CMTR(s), or negative shmiR as control. After 72 h, the growth medium was evaluated for luminescence activity (Figure 3C). As a control for the experiment, we also performed luciferase assay for non-modified HEK 293 Flp-In T-REx cells, cultured either in the presence doxycycline or without addition of an inducer. We compared the results obtained for the sh neg cell line and we concluded that neither treatment with doxycycline nor expression of negative shmiRs and fluorescent proteins impact protein production from exogenous mRNA (results for all tested cell lines are presented in Supplementary Figure S10). Irrespective of the depleted methyltransferase(s), none of the tested IVT mRNAs preferentially interacted with the cellular translation machinery (Figure 3C). Thus, these data were consistent with our MST measurements.

### The second transcribed nucleotide 2′-*O*-methylation prevents RNA from decapping by hDXO but not by hDCP2

**Figure 4.**
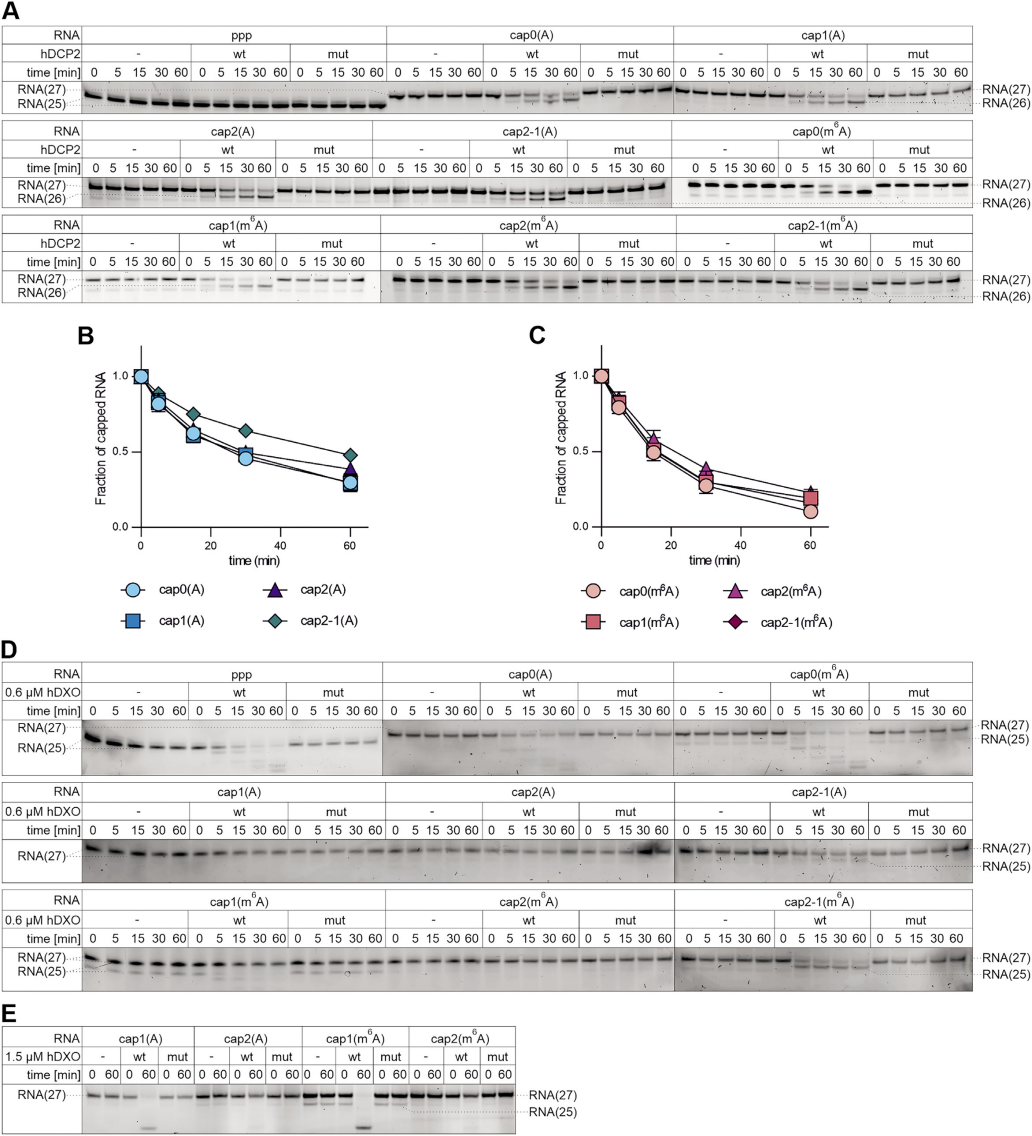
2′-*O*-Methylation of the second transcribed nucleotide prevents RNA from decapping by DXO but not by DCP2. (**A–C**) Short capped RNAs were subjected to treatment with hDCP2 (wild type or mutant) over a 60 min time-course. Reactions without enzyme served as controls. Aliquots from the indicated time points were resolved on a polyacrylamide gel and bands corresponding to capped transcripts (27 nt long) and to RNAs decapped by hDCP2 action (26 nt long) were quantified densitometrically. (**A**) Representative polyacrylamide gel analyses obtained for all tested capped RNAs (two additional repetitions with wild-type hDCP2 of this experiment are shown in Supplementary Figure S12). (**B** and **C**) Quantitative results for all studied RNAs. The fraction of capped RNA remaining in the total RNA was plotted as a function of time. Data points represent mean values ± SD from triplicate experiments. (**D**) Short capped RNAs were subjected to treatment with hDXO (wild type or mutant) over a 60 min time-course. Reactions without enzyme served as controls. Aliquots from the indicated time points were resolved on a polyacrylamide gel. (**E**) Experimental set-up as in (**D**); however, a 2.5-fold higher hDXO concentration was used.

The presence of a cap not only enables efficient translation but also protects the transcript 5′ end from degradation. Thus, we investigated whether differences in protein production from reporter mRNAs may partially arise from the different susceptibilities of transcripts to decapping. In mammalian cells, the DCP1/DCP2 heterodimer, where DCP2 is a catalytically active subunit, is the main factor responsible for cap structure elimination from transcripts designated for degradation (17). To investigate the susceptibility of RNAs bearing differently methylated caps to DCP2-mediated decapping, short transcripts capped with tri- and tetranucleotide cap analogues were prepared. Capped RNAs were then incubated with recombinant active and catalytically inactive versions of the hDCP2 protein (Supplementary Figure S11A) followed by polyacrylamide gel analysis of the products collected over time (Figure 4A). We observed a reduction in the band intensity corresponding to capped transcripts with time, and the appearance of bands representing transcripts deprived of m^7^GDP. Importantly, this process was observed for active hDCP2 alone, indicating that decapping activity can be attributed solely to hDCP2. Moreover, to directly compare the susceptibility to hDCP2 action, the fraction of capped RNAs that remained in each sample was plotted as a function of time (Figure 4B, C). The presence of one or two 2′-*O*-methylation(s) within a cap did not affect susceptibility to hDCP2 activity *in vitro*. This result is in agreement with our previous studies, in which we showed that 2′-*O*-methylation of the first transcribed nucleotide did not influence the vulnerability of transcripts to decapping, regardless of the identity of the first transcribed nucleotide (14). Moreover, we observed that the presence of *N*6-methylated adenosine at the m^7^G-adjacent position modestly increased RNA susceptibility to hDCP2 activity, irrespective of the cap 2′-*O*-methylation status (Supplementary Figure S13A–D), with the exception of transcripts with cap1 structure, for which susceptibility to decapping by hDCP2 was comparable (Supplementary Figure S13B).

Another enzyme involved in decapping of mammalian transcripts is the DXO protein (18). In contrast to DCP2, DXO not only decaps transcripts, but also has 5′-3′ exonucleolytic activity, through which it can eliminate RNA designated for degradation. Moreover, DXO accepts uncapped 5′-triphosphorylated RNA as a substrate and, unlike DCP2, it cleaves off the entire cap structure, releasing dinucleotides linked via the triphosphate bridge. Recently, it has been shown that 2′-*O*-methylation present in cap1 confers RNA resistance to hDXO decapping activity and further degradation (19). Moreover, the presence of the 2′-*O*-methyl group in RNA bodies hampers hDXO exonucleolytic activity. Thus, we hypothesized that the simultaneous presence of 2′-*O*-methylation at the first and second transcribed nucleotides can make RNAs more resistant compared with transcripts with cap1. To evaluate this hypothesis, we performed *in vitro* activity assays using active or catalytically inert versions of the recombinant hDXO protein (Supplementary Figure S11B) and differently capped short RNAs. The reaction products from the time-course were resolved using polyacrylamide gels (Figure 4D). During the reaction, we observed decapping and subsequent degradation of RNA bearing cap0, regardless of the identity of the m^7^G-adjacent nucleotide (A versus m^6^A). As expected, the presence of the 2′-*O*-methyl group at the first transcribed nucleotide alone or at both the first and second transcribed nucleotides protected transcripts from hDXO activity. Interestingly, we did not observe a decrease in decapping rate for transcripts with 2′-*O*-methylation at the second transcribed nucleotide alone; however, the presence of a methyl group at this position efficiently blocked exonucleolytic degradation; accumulation of RNA deprived of the cap structure [RNA(25)] in experiments with transcripts possessing cap2-1 was noticeable. Moreover, we attempted to differentiate the susceptibility of transcripts with cap1 and cap2 to degradation by employing a higher hDXO concentration. Under these experimental conditions, RNA bearing 2′-*O*-methylation at both the first and second transcribed nucleotides was more resistant to hDXO action than transcripts with cap1 (Figure 4E).

### 2′-*O*-Methylation of the second transcribed nucleotide and *N*6-methylation of adenosine as the first transcribed nucleotide contribute to RNA immune evasion

2′-*O*-Methylation of the first transcribed nucleotide is a known mark distinguishing ‘self’ from ‘non-self’ transcripts (9,10). IFIT proteins are the main factors responsible for regulating cap-dependent translation in stressed cells (13,37). IFIT1 binds directly to the mRNA 5′ end and competes with eIF4E for binding to the *N*7-methylguanosine triphosphate cap. The 2′-*O*-methylation in cap1 precludes mRNA–IFIT1 interaction and translational inhibition by this protein (12,13). However, methylation of the second transcribed nucleotide may also contribute to this process (38). To evaluate this hypothesis, we examined the protein production levels in stressed cells transfected with IVT mRNAs encoding *Gaussia* luciferase bearing differently methylated caps. To induce stress conditions and cause up-regulation of genes involved in innate immune response, which influence translation, we employed IFNα treatment, which is widely used to increase the production of antiviral proteins, including IFITs (13,39). To ascertain whether our experimental set-up induced up-regulation of antiviral protein production in A549 cells, we verified the expression of several antiviral genes in the presence of increasing amounts of IFNα by RT–qPCR (Supplementary Figure S14). Our results showed that up-regulation of IFITs, 2′-5′-oligoadenylate synthetase 1 (OAS1) and protein kinase R (PKR) correlated positively with the amount of IFNα added to the cell culture medium.

**Figure 5.**
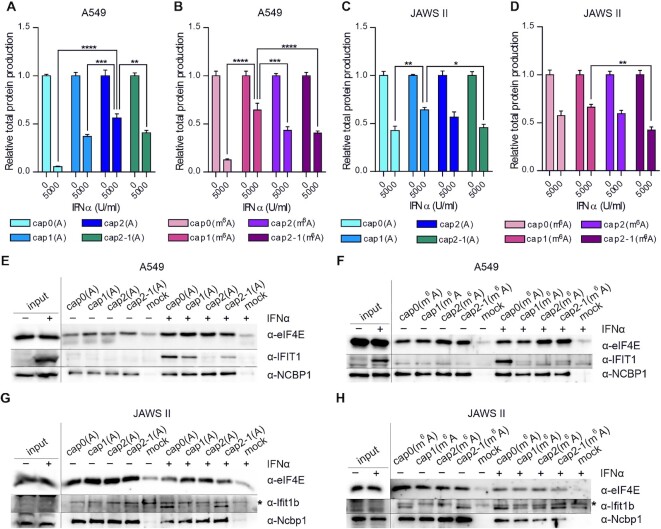
Methylation of the first transcribed nucleotides in mRNA only partially prevents protein level decrease under IFN-induced stress. Relative protein production levels 72 h after a 5 h IFNα pre-treatment in (**A** and **B**) A549 and (**C** and **D**) JAWS II cells. Data were analysed for three independent replicates (each experiment consisted of three technical replicates). Bars for each transcript represent the mean value ± SEM normalized to protein production in mock-treated cells. Statistical significance: *P <0.05, **P <0.01, ***P <0.001, ****P <0.0001 (one-way ANOVA with Turkey's multiple comparisons test) (data presenting changes in relative protein production with increasing concentration of IFNα are shown in Supplementary Figures S15 and S16 for A549 and JAWS II cells, respectively). Co-purification of endogenous proteins from lysates of IFNα-treated (**E** and **F**) A549 (IFNα concentration was 500 U/ml) and (**G** and **H**) JAWS II (IFNα concentration was 5000 U/ml) cells with biotinylated RNA bearing cap0, cap1, cap2 or cap2-1 with (**E** and **G**) A or (**F** and **H**) m^6^A as the first transcribed nucleotide. Human eIF4E, IFIT1 and NCBP1 or murine counterparts (Ifit1b: lower band) were detected in precipitates by western blotting. A 1/15th and 1/21st volume of lysate used for each incubation with beads was loaded as input sample for (**E–G**) and (**H**), respectively; a 1/3rd volume of each eluate was loaded; shorter exposure is presented for input samples than for eluates; *non-specific band.

Next, we examined the changes in protein production levels for differently capped IVT mRNAs encoding *Gaussia* luciferase in A549 cells treated with increasing amounts of IFNα (Figure 5A, B; Supplementary Figure S15). As the IFNα concentration increased, we observed a gradual decrease in protein production for all tested IVT mRNAs (Supplementary Figure S15); however, the greatest decrease in protein biosynthesis was observed for transcripts bearing cap0. As expected, 2′-*O*-methylation of the first transcribed nucleotide was associated with greater protein production levels compared with transcripts with the respective cap0 structures (Figure 5A, B; Supplementary Figure S15). Intriguingly, the effect of 2′-*O*-methylation of the second transcribed nucleotide depended on the identity of the first transcribed nucleotide (A versus m^6^A). If adenosine was the first transcribed nucleotide [cap2(A)], the presence of two 2′-*O*-methylations within the cap resulted in the least reduction in luminescence. In contrast, the combination of two 2′-*O*-methyl groups and *N*6-methyladenosine [cap2(m^6^A)] caused a decrease in protein production compared with IVT mRNA bearing the respective cap1 structure. Moreover, experiments with IVT mRNA containing the cap2-1 structure indicated that the presence of 2′-*O*-methylation at the second transcribed nucleotide alone was sufficient to ensure high protein production levels under stress conditions. Protein production from transcripts with cap2-1(A) was similar to that from IVT mRNA bearing cap1(A), whereas luminescence measured for cells transfected with transcripts bearing cap2-1(m^6^A) was comparable with that from cells transfected with IVT mRNA with cap2(m^6^A).

Subsequently, we decided to check how the treatment with increasing concentrations of IFNα influences protein production levels in another cell line. To this end, we chose murine JAWS II cells, as it is known that the cap methylation status in this cell line exerts quite different effects on protein biosynthesis yield compared with A549 cells. Moreover, the 5′ end of foreign RNA in mice is not recognized by the IFIT1 orthologue, which has been lost during evolution, but by a paralogous Ifit1b, which has been further duplicated twice, giving rise to Ifit1b2 and Ifit1b3 [according to the nomenclature introduced by Daugherty *et al.* (40)]. Recently, it has been shown that Ifit1b and Ifit1b2 are cap-binding proteins, whereas Ifit1b3 acts as a stimulatory cofactor of the two former proteins (41). Intriguingly, it is suggested that the presence of 2′-*O*-methylation of only the first transcribed nucleotide completely prevents Ifit1b from binding the mRNA 5′ end (40). Other studies showed that Ifit1b2 is still able to inhibit translation of mRNA bearing the cap1 structure in rabbit reticulocyte lysate (41). However, it is still uncertain how exactly protein production is modulated by the cap methylation status in murine cells under stress conditions. Thus, we monitored changes in luminescence in JAWS II cells treated with increasing amounts of IFNα. In contrast to A549 cells (Figure 5A, B; Suppelementary Figure S15), we did not observe a strong gradual decrease in protein production in the JAWS II cell line (Figure 5C, D: Supplementary Figure S16). The greatest difference in protein biosynthesis yield was noted for the highest concentration of interferon used, i.e. 5000 U/ml (Supplementary Figure S16). Interestingly, the level of luminescence was similar for all tested IVT mRNAs, regardless of the identity of the m^7^G-adjacent nucleotide (A versus m^6^A). However, the smallest decline in protein production was observed for transcripts with a 2′-*O*-methylated first transcribed nucleotide only (cap1).

Since IFIT1 competes with eIF4E for mRNAs in cells exposed to stress stimuli, we analysed the contribution of each cap methylation type to IFIT1 binding. To this end, we used the previously mentioned approach based on biotinylation of differently capped transcripts as baits for proteins from lysates prepared using IFNα-treated and untreated A549 and JAWS II cells. RNP complexes formed on capped transcripts were purified using streptavidin beads, and IFIT1 levels were analysed by western blotting. As expected, in the case of human cells, the presence of 2′-*O*-methylation at adenosine as the first transcribed nucleotide [cap1(A)] decreased the affinity of capped RNA for IFIT1 (Figure 5E). The 2′-*O*-methylation of only the second transcribed nucleotide [cap2-1(A)] had a similar impact on IFIT1 binding. Importantly, IFIT1 was unable to bind to RNA bearing cap2(A). 2′-*O*-Methylation did not affect eIF4E binding or interaction with the nuclear cap-binding complex. These results were in line with the protein production levels measured for differently capped reporter transcripts in IFNα-treated A549 cells (Figure 5A, B; Supplementary Figure S15). Interestingly, for transcripts with m^6^A as the first transcribed nucleotide, the presence of only one 2′-*O*-methylation made RNA almost unrecognizable for IFIT1 (Figure 5F), indicating that the identity of the m^7^G-adjacent nucleotide affects RNA recognition by a major effector of the cellular immune defence system. Moreover, 2′-*O*-methylation of the second transcribed nucleotide either resulted in a synergistic reduction in IFIT1 binding [cap2(m^6^A)] or ensured similar affinity towards IFIT1 [cap2-1(m^6^A)] when compared with RNA bearing cap1(m^6^A). Intriguingly, we did not observe any difference in RNA 5′ end recognition by Ifit1b due to the identity of m^7^G-adjacent nucleotide (A versus m^6^A) using JAWS II cell lysate (Figure 5G, H). Moreover, the presence of 2′-*O*-methylation at the first transcribed nucleotide only was sufficient to completely abolish Ifit1b binding, whereas the occurrence of the same modification at the second transcribed nucleotide did not influence association of the RNA 5′ end with this protein. Taken together, 2′-*O*-methylation of the second transcribed nucleotide as well as *N*6-methylation of adenosine as the first transcribed nucleotide in human cells may serve as determinants defining transcripts as ‘self’, and contribute to transcript escape from the host innate immune response. Instead, in murine cells, 2′-*O*-methylation of only the first transcribed nucleotide marks transcripts as ‘self’, at least regarding RNA binding by Ifit1b.

## DISCUSSION

IVT mRNA can be used as a therapeutic agent in preventive vaccines, cancer immunotherapy or gene replacement therapy (42–44). However, the utility of IVT RNA exceeds potential clinical applications. IVT RNAs can also serve as valuable tools for studying RNA-related processes *in vitro* and *in vivo* (45). Preparing IVT RNA with different modifications enables the analysis of their impact on biological processes of interest. Among the modifications that can be studied using IVT RNA are those in the cap structure, i.e. ribose 2′-*O*-methylation(s) of the first and second transcribed nucleotide(s), as well as *N*6 nucleobase methylation of adenosine when present as the first transcribed nucleotide. Importantly, the use of differently capped transcripts not only enables a straightforward comparison of how each cap methylation influences RNA-related processes but also enables the study of the interplay between selected modifications. Until recently, the introduction of modifications in the cap structure of IVT RNA was a time-consuming and elaborate process. For instance, transcripts with *N*6,2′-*O*-dimethyladenosine as the first transcribed nucleotide and bearing 2′-*O*-methylation at the second transcribed nucleotide were obtained using three consecutive enzymatic reactions. Moreover, the generation of such transcripts requires a detailed analysis of cap modification efficiency after each enzymatic step. Recently, the process of obtaining RNA bearing modifications in the cap structure has been considerably simplified using new molecular tools developed by us and other researchers, i.e. trinucleotide cap analogues as initiators of *in vitro* transcription reactions, that enable preparation of transcripts with modifications at the first transcribed nucleotide (14,20). However, the application of trinucleotide cap analogues does not enable straightforward generation of transcripts bearing 2′-*O*-methylation at the second transcribed nucleotide. To fully appreciate the impact of cap methylation on RNA-related processes, we have described the generation of new molecular tools, namely tetranucleotide cap analogues, which enable direct preparation of RNA possessing a cap also 2′-*O*-methylated at the second transcribed nucleotide.

Combining the previously generated tri- (14) and newly synthesized tetranucleotide cap analogues described herein, we were able to systematically and comprehensively study the impact of all three known cap methylations on protein biosynthesis. Our data showed that the presence of 2′-*O*-methylation at the first transcribed nucleotide (cap1) either boosted (A549 and JAWS II cells) or did not change (THP-1 cells) protein production levels compared with those obtained for IVT mRNA bearing the respective cap0 counterparts, regardless of the identity of the first transcribed nucleotide. The only exception for cap1 relative to cap0 was observed in the 3T3-L1 cell line, where the protein production level for transcripts with adenosine as the m^7^G-adjacent nucleotide was unchanged, whereas mRNA with cap1(m^6^A) showed a modestly greater protein yield than transcript bearing cap0(m^6^A). These results are in line with our previous observations showing that 2′-*O*-methylation enhances the biosynthesis process in JAWS II but not in HeLa or 3T3-L1 cells (14). Importantly, our data are also consistent with recent studies on the role of PCIF1, a cap methyltransferase responsible for *N*6-methylation of 2′-*O*-methylated adenosine as the first transcribed nucleotide, in mRNA metabolism (6,7). Sendinc *et al.* and Boulias *et al.* showed that the loss of PCIF1 resulted in either an increase or no change in the translation of transcripts, which naturally possess m^6^A_m_ in the cap structure, in the MEL624 human melanoma cell line (7) and HEK 293T cells, respectively (6). We observed that change of A to m^6^A in the context of cap1 led to a decrease in protein production (3T3-L1 and JAWS II cells) or did not affect protein biosynthesis at all (THP-1 and A549 cells).

The presence of the 2′-*O*-methyl group at the first and second transcribed nucleotides (cap2) resulted in decreased protein production levels in the tested cell lines compared with levels for transcripts bearing the respective cap1 counterparts. The greatest decrease in protein biosynthesis was observed in JAWS II cells, in which protein yield for IVT mRNA with cap2 was >30 times lower than for transcripts with cap1. However, the magnitude of reduction depended on the identity of the first transcribed nucleotide. Protein production levels for transcripts with cap2(m^6^A) were less affected than for mRNA with cap2(A) relative to protein levels for transcripts bearing the respective cap1 analogues. Interestingly, in A549 cells, the occurrence of two 2′-*O*-methylations in the context of *N*6-methyladenosine ensured the same level of protein production as the yield from mRNA with cap1(m^6^A). Taken together, our results indicate that protein production regulation via cap methylation is cell line dependent. Moreover, the 2′-*O*-methylation of the second transcribed nucleotide is the major player in this mechanism.

To understand why protein production levels differed substantially among the tested cell lines, we sought to identify proteins that interacted with differently capped transcripts in A549 and JAWS II cells. We analysed why the presence of 2′-*O*-methylation at the first transcribed nucleotide (cap1) boosted protein production in JAWS II, whereas two 2′-*O*-methylations at the first and second transcribed nucleotides (cap2) hampered protein biosynthesis in this cell line. In contrast, the same set of modifications modestly affected protein production levels in A549 cells. Among the identified cap-interacting proteins, we could not determine factors associated with transcripts bearing a 2′-*O*-methylated cap structure that impacted protein production levels in JAWS II cells. The lack of a clear indication of which proteins may be responsible for modulating protein production levels in JAWS II cells may be due to the pull-down protocol utilized herein, i.e. since we analysed RNP complexes formed on differently capped RNA in a post-cell lysis approach, it cannot be excluded that some non-specific interactions could occur preventing identification of specific intracellular interactors (46). Nevertheless, a meta-analysis of the identified proteins revealed that RNA–protein interactomes for differently capped transcripts varied substantially in JAWS II cells, whereas, in A549 cells, interactomes were similar. The biggest discrepancy among interactomes of capped RNAs was identified in JAWS II cells for transcripts differing in the 2′-*O*-methylation status of the second transcribed nucleotide. The set of proteins associated with RNA bearing either cap2(A) or cap2-1(A) were more similar to each other than to the interactomes identified for transcripts with cap1(A) or cap0(A). Interestingly, the interactomes of transcripts bearing cap0(A) and cap1(A) from JAWS II cells were very similar even though a significant difference in reporter protein production level was observed between them. Based on these results, we can assume that only protein biosynthesis was repressed (cap0/cap1 versus cap2/cap2-1) at the level of transcript 5′ end recognition or translation initiation. In contrast, an increase in protein production due to 2′-*O*-methylation of the first transcribed nucleotide (cap0 versus cap1) was not caused at the level of translation initiation. Interestingly, similar proteomic analyses have recently been performed by other researchers to characterize proteins associated with RNA bearing differently modified 5′ ends; however, in contrast to our results, Habjan *et al.* compared uncapped RNA with its counterparts bearing only cap0 or cap1 (13). Nevertheless, the results of MS/MS analysis from their study are in line with our observations, i.e. no difference was observed in the identified capped RNA–protein interactomes in human cervical carcinoma (HeLa) and mouse embryonic fibroblast (MEF) cells. Taken together, proteomics studies support the hypothesis that cap methylation status influences protein biosynthesis in a cell-dependent manner, and we show that the major determinant affecting protein production is 2′-*O*-methylation of the second transcribed nucleotide.

As protein production is affected differently in various cell lines by the cap methylation status, and 2′-*O*-methylation of the second transcribed nucleotide is the major player in this mechanism, we asked whether this modification could influence the affinity of transcripts towards the translation initiation machinery. Previously, we showed that none of the modifications of the first transcribed nucleotide affected the stability of the cap analogue–eIF4E complex (14). In this study, we expanded this analysis to transcripts with all possible methylation combinations within the cap structure and investigated RNA affinity towards eIF4E. We found that none of the studied cap methylations strongly influenced the stability of the RNA–eIF4E complex. Only *N*6-methylation of adenosine as the first transcribed nucleotide, irrespective of the cap 2′-*O*-methylation status, slightly but significantly influences RNA binding to eIF4E. We showed previously that the presence of m^6^A as an m^7^G-adjacent nucleotide within trinucleotide cap analogues induces conformational changes in the C-terminal region of eIF4E upon its binding, as compared with trinucleotide cap analogues with adenosine as the first transcribed nucleotide (14). These changes can affect the proposed path taken by the RNA chain along the eIF4E surface (28) and, as a consequence, may influence the affinity of eIF4E towards RNAs with *N*6-methyladenosine within the cap structure. This is probably a reason why changes in the eIF4E C-terminal domain have an impact on RNA binding but not on an interaction with trinucleotide cap analogues alone. We also performed competition experiments, in which exogenous reporter transcripts competed with endogenous mRNAs for the translation machinery in cells depleted of cap 2′-*O*-methyltransferases, to further study the impact of cap 2′-*O*-methylation(s) on the affinity of IVT mRNAs towards the cellular translation machinery. Knockdown of cap methyltransferases (CMTR1 and CMTR2, either individually or simultaneously) did not influence protein production levels for differently capped IVT mRNAs, indicating that regardless of the cap 2′-*O*-methylation status, the translation machinery showed no preference for the different transcript types. Therefore, to explain how the cap methylation status modulates RNA-related processes, we investigated transcript stability.

The cap not only ensures efficient protein biosynthesis, but also protects the RNA 5′ end from uncontrolled exonucleolytic attack. DCP2 is the main decapping enzyme responsible for cap elimination from transcripts designated for degradation (17). Biochemical characterization revealed that the cap methylation status did not affect RNA susceptibility to decapping by hDCP2. However, transcripts bearing adenosine as the first transcribed nucleotide were slightly more resistant to hDCP2 action than RNA with *N*6-methyladenosine as the first transcribed nucleotide, regardless of the cap 2′-*O*-methylation status. This difference in susceptibility of transcripts to hDCP2 activity is supported by recent genome-wide studies in 293T cells, in which the level of transcripts, which naturally possess m^6^A_m_ in the cap structure, was increased upon PCIF1 knockout (5). However, these observations have been questioned by other researchers. Sendinc *et al.* reported no changes in transcription or mRNA stability upon PCIF1 knockout in MEL624 cells (7). Intriguingly, we noticed that 2′-*O*-methylation impacted susceptibility to decapping by the hDXO enzyme. The presence of only one 2′-*O*-methyl group impaired hDXO action on the RNA bearing the cap1 structure compared with cap0, regardless of the identity of the first transcribed nucleotide (A versus m^6^A). Our observation is in line with a previous report by Picard-Jean *et al.*, which showed that 2′-*O*-methylation of the first transcribed nucleotide impaired the transcript’s susceptibility to decapping by hDXO. Moreover, Picard-Jean *et al.* also noted that internal 2′-*O*-methylation drastically reduced hDXO exonucleolytic activity towards transcripts with such modifications within the RNA body (19). We found that although 2′-*O*-methylation of only the second transcribed nucleotide (cap2-1) did not affect hDXO decapping activity compared with its action on RNA with cap0, subsequent exonucleolytic degradation of the decapped transcript was impaired. Thus, we envisaged that the decapping rate of transcripts with cap2 would be similar to that for RNAs with cap1 analogues; however, 2′-*O*-methylation of the second transcribed nucleotide can block subsequent exonucleolytic hydrolysis. Interestingly, we found that the decapping rate was reduced for transcripts containing cap2 relative to RNAs bearing cap1 analogues. Apart from playing a role in the removal of improperly capped transcripts (17), DXO was recently recognized as an antiviral factor responsible for the restriction of hepatitis C virus (HCV) replication (47). Therefore, we concluded that 2′-*O*-methylation of the second transcribed nucleotide may be an additional determinant of molecule ‘selfness’ that enables endogenous transcripts to escape from being recognized by the innate immune response.

It is widely assumed that 2′-*O*-methylation of the first transcribed nucleotide is not sufficient to completely protect mRNAs from being recognized as ‘non-self’ by the cellular immune defence system (38,40,48). Therefore, it is an open question what are the other RNA features that mark transcripts as ‘self’. One such feature could be an additional 2′-*O*-methylation present at the second transcribed nucleotide. Abbas *et al.* showed that only transcripts bearing caps with both m^7^G-adjacent nucleotides 2′-*O*-methylated are fully resistant *in vitro* to IFIT1 recognition (38), which plays a crucial role in distinguishing ‘self’ from ‘non-self’ RNAs (49). Moreover, based on the crystal structure of the IFIT1 in complex with capped RNA, the authors proposed that the presence of *N*6,2′-*O*-dimethyladenosine instead of 2′-*O*-methyladenosine could also provide full protection against IFIT1 binding; however, they did not provide any experimental data to support this hypothesis (38). Tartell *et al.* recently showed that the presence of m^6^A_m_, but not A_m_, as the first transcribed nucleotide of vesicular stomatitis virus (VSV) and rabies virus (RABV) transcripts enabled efficient viral replication, presumably by impeding the recognition of viral RNAs by the effectors of the innate immune system (50). Using IFNα treatment in A549 cells, we showed that protein production for IVT mRNA bearing cap2 was less affected than for transcripts with cap1; however, this effect was dependent on the identity of the first transcribed nucleotide. The combination of *N*6-methylation and 2′-*O*-methylation of adenosine as the first transcribed nucleotide ensured the highest protein production level under stress conditions. However, *N*6-methylation of adenosine as the first transcribed nucleotide did not guarantee a high level of protein biosynthesis in stressed cells, and the reduction in protein production was comparable for IVT mRNA bearing cap0(m^6^A) and cap0(A). The result indicates that 2′-*O*-methylation is indispensable for efficient protein production under stress conditions, whereas *N*6-methylation positively stimulates protein biosynthesis only if adenosine is 2′-*O*-methylated. Moreover, we found that the presence of a single 2′-*O*-methyl group, regardless of the position of the modified nucleotide, was sufficient to ensure high protein production levels in stressed cells. This observation can be crucial for understanding how cell fitness is regulated by exposure to stress. It was shown that CMTR1 controls mRNA stability and promotes protein expression of certain IFN-induced genes in stressed cells (51,52). The moderate influence of CMTR1 knockdown on the protein production level may be explained by the redundant functions of cap 2′-*O*-methylations present at different positions. We assume that upon stress conditions, a high protein production level can be achieved even for transcripts lacking 2′-*O*-methylation at the first transcribed nucleotide; however, this can happen provided that the same modification is also present at the second transcribed nucleotide.

Moreover, we found that additional methylations of cap1(A) counteracted the recognition of transcripts by IFIT1. The presence of either cap2(A) or cap1(m^6^A) causes transcripts to be undetectable by IFIT1. Interestingly, the position of the 2′-*O*-methyl group, whether linked to the first or to the second transcribed nucleotide, did not influence the efficiency of RNA escape from recognition by IFIT1. This observation is consistent with the results of our protein production studies in stressed cells. Taken together, both 2′-*O*-methylation of the second transcribed nucleotide and *N*6-methylation of adenosine as the first transcribed nucleotide may serve as additional determinants defining transcripts as ‘self’.

Finally, we conducted analogous studies for the JAWS II murine cell line; however, in this case, we did not observe substantial differences in protein production levels for differently capped transcripts in stressed cells. It was found that, regardless of the number and position of methyl groups, all tested IVT mRNAs yielded similar amounts of reporter protein. Subsequently, we tested how the cap methylation status influenced recognition of transcript by murine Ifit1 proteins. In contrast to human cells, in murine cells two Ifit1 proteins binding capped foreign RNA, namely Ifit1b and Ifit1b2, are present (40,41). It is postulated that Ifit1b displays a preference towards RNAs with cap0 (40), whereas Ifit1b2 binds only transcripts bearing the cap1 structure (41). Due to the lack of commercially available antibodies against Ifit1b2, we were only able to investigate Ifit1b affinity towards RNA with differently methylated cap structures here. Based on our pull-down experiments, it appears that transcripts with a 2′-*O*-methylated first transcribed nucleotide (cap1), but not a 2′-O-methylated second transcribed nucleotide (cap2-1), escaped recognition by Ifit1b in murine cells, while in human cells the 2′-*O*-methyl group at the second transcribed nucleotide only was sufficient to almost completely abolish IFIT1 binding to RNA. Intriguingly, in contrast to A549 cells, some basal level of Ifit1b expression was noticeable in the JAWS II cell line, which is in agreement with the observation of Zhang *et al.*, who reported that cultured dendritic cells could spontaneously express IFIT proteins (53). This finding may partially explain why protein production levels were not as strongly affected in JAWS II cells as in A549 cells upon IFNα treatment. Collectively, these observations highlight striking differences between closely related RNA metabolic processes even within the same group of vertebrates and emphasize that general conclusions based on studies conducted on a limited number of model organisms should be drawn with extreme caution.

To summarize, we have shed light on the role of RNA cap methylation. The new tools presented here enabled the generation of efficiently capped transcripts with structures modified in a defined manner. We demonstrated the impact of specific modifications and their combinations on protein production levels, which may differ in various cell lines. We also investigated an interplay between cap methylations in the context of particular biochemical features of capped transcripts. We postulate that in human cells the combination of cap methylations enhances the identity of RNA as a ‘self-molecule’ for cellular recognition factors that contribute to immune evasion.

## DATA AVAILABILITY

The mass spectrometry proteomics data have been deposited in the ProteomeXchange Consortium via the PRIDE (54) partner repository with the dataset identifier PXD028635 and PXD028636. Other relevant data are included in the paper and accompanying Supplementary data, or are available from the corresponding author upon reasonable request.

## Supplementary Material

gkac722_Supplemental_Files

## Notes

Present address: Renata Kasprzyk, Department of Chemistry, University of Konstanz, Universitätsstraße 10, D-78457 Konstanz, Germany.
