# Supplementary material for: 2′-O-Methylation of the second transcribed nucleotide within the mRNA 5′ cap impacts the protein production level in a cell-specific manner and contributes to RNA immune evasion
Source: Nucleic Acids Res. 2022 Aug 26;50(16):9051–71. doi: 10.1093/nar/gkac722 (PMC9458431; doi:10.1093/nar/gkac722)
Supplement: gkac722_Supplemental_Files [file gkac722_supplemental_files.zip › SI_final.pdf]

2'-*O*-methylation of the second transcribed nucleotide within mRNA 5' cap impacts protein production level in a cell-specific manner and contributes to RNA immune evasion

Karolina Drazkowska[1], Rafal Tomecki[2,3], Marcin Warminski[4], Natalia Baran[1,5], Dominik Cysewski[2,6], Anaïs Depaix[1], Renata Kasprzyk[1], Joanna Kowalska[4], Jacek Jemielity[1], Pawel J. Sikorski[1,5,*]

[1] Centre of New Technologies, University of Warsaw, Banacha 2c, 02-097 Warsaw, Poland
[2] Institute of Biochemistry and Biophysics, Polish Academy of Sciences, Pawinskiego 5a, 02-106 Warsaw, Poland
[3] Institute of Genetics and Biotechnology, Faculty of Biology, University of Warsaw, Pawinskiego 5a, 02-106 Warsaw, Poland
[4] Division of Biophysics, Institute of Experimental Physics, Faculty of Physics, University of Warsaw, Pasteura 5, 02-093 Warsaw, Poland
[5] Department of Environmental Microbiology and Biotechnology, Institute of Microbiology, Faculty of Biology, University of Warsaw, Miecznikowa 1, 02-096 Warsaw, Poland
[6] Clinical Research Centre, Medical University of Bialystok, M. Sklodowskiej-Curie 24a, 15-276 Bialystok, Poland

* To whom correspondence should be addressed. Tel: +48 22 55 43775; Fax: +48 22 55 43771; Email: pawelsikorski@uw.edu.pl

Present Address: [Renata Kasprzyk], Department of Chemistry, University of Konstanz, Universitätsstraße 10, 78457 Konstanz, Germany

SUPPLEMENTARY DATA

A

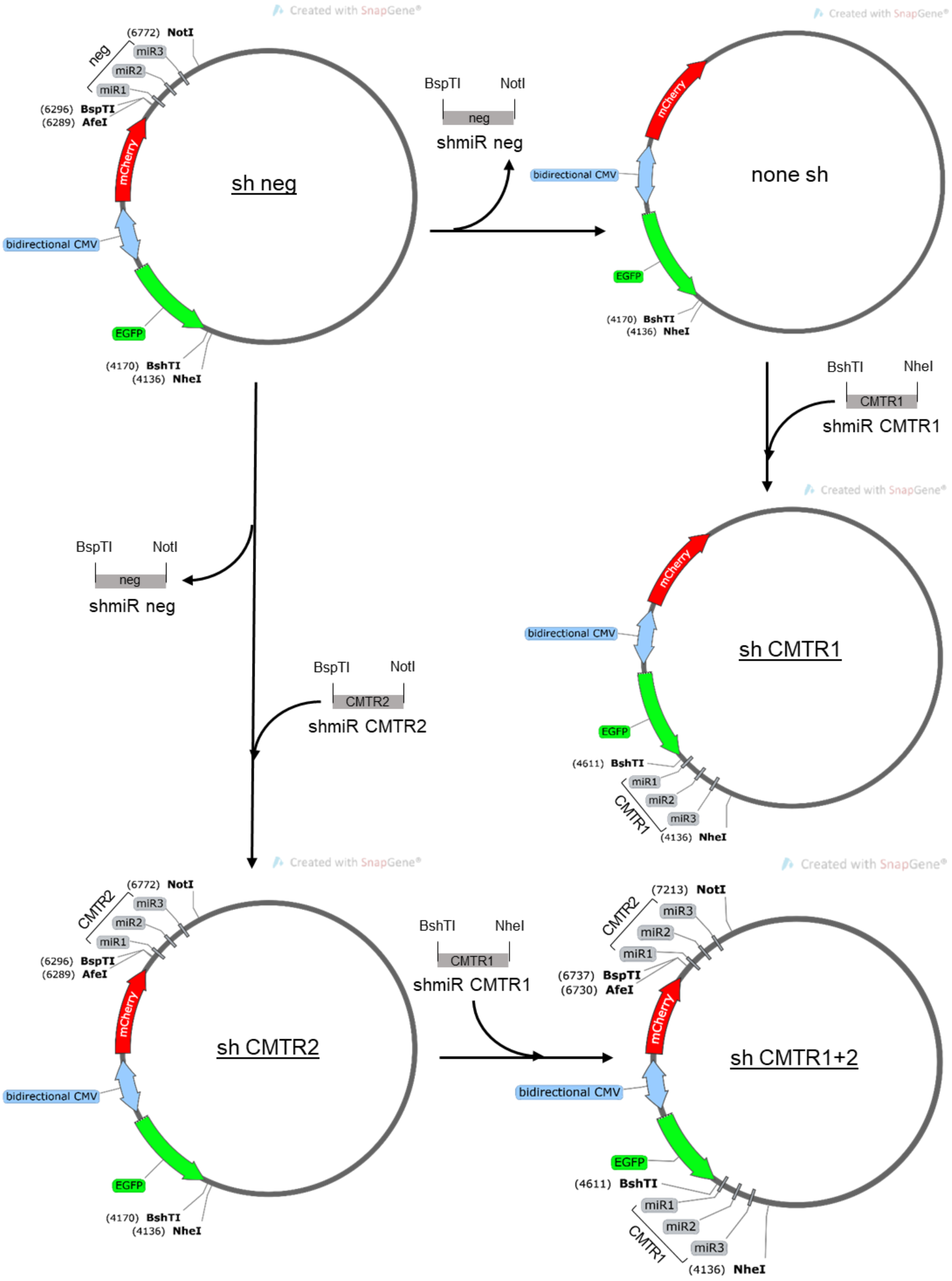

Created with SnapGene®
(6772) NotI
neg
miR3
miR2
miR1
(6296) BspTI
(6289) AfeI
mCherry
sh neg
bidirectional CMV
EGFP
(4170) BshTI
(4136) NheI
BspTI
NotI
neg
shmiR neg
none sh
(4170) BshTI
(4136) NheI
BshTI
NheI
CMTR1
shmiR CMTR1
BspTI
NotI
neg
shmiR neg
BspTI
NotI
CMTR2
shmiR CMTR2
sh CMTR1
(4611) BshTI
CMTR1
(4136) NheI
CMTR2
sh CMTR2
BshTI
NheI
CMTR1
shmiR CMTR1
(7213) NotI
(6737) BspTI
(6730) AfeI
sh CMTR1+2

B

accggtTGCTGCTGGAGGCTTGCTGAAGGCTGTATGCTGATAATCATCTTTCTCTTTCCCGTTTTGGCCACTGACTG
ACGGGAAAGAAAGATGATTATCAGGACACAAGGCCTGTTACTAGCACTCACATGGAACAAATGGCCCAGATCCTG
GAGGCTTGCTGAAGGCTGTATGCTGTTTTCGCCAGAGCTTTGATCTGTTTTGGCCACTGACTGACAGATCAAATC
TGGCGAAAACAGGACACAAGGCCTGTTACTAGCACTCACATGGAACAAATGGCCCAGATCCTGGAGGCTTGCTG
AAGGCTGTATGCTGAATCTTCTCCATCTCTTCCAGGTTTTGGCCACTGACTGACCTGGAAGATGGAGAAGATTCA
GGACACAAGGCCTGTTACTAGCACTCACATGGAACAAATGGCCCAGATCTGGCCGCACTCGAGATATCTAGTGAT
CTAGAGGGCCCGCGGTTCGCTGATgctagc

C

cttaagTGCTGCTGGAGGCTTGCTGAAGGCTGTATGCTGGTTTCTTATCACTCAGTAGGTGTTTTGGCCACTGACTG
ACACCTACTGTGATAAGAAACCAGGACACAAGGCCTGTTACTAGCACTCACATGGAACAAATGGCCCAGATCCTG
GAGGCTTGCTGAAGGCTGTATGCTGAAATATTTGTTCCTCTGCCCAGTTTTGGCCACTGACTGACTGGGCAGAAA
CAAATATTTCAGGACACAAGGCCTGTTACTAGCACTCACATGGAACAAATGGCCCAGATCCTGGAGGCTTGCTGA
AGGCTGTATGCTGTTTCTTCTCTCTCTCTTTGAAGTTTTGGCCACTGACTGACTTCAAAGAGAGAGAAGAAACAGG
ACACAAGGCCTGTTACTAGCACTCACATGGAACAAATGGCCCAGATCTGGCCGCACTCGAGATATCTAGTGATCT
AGAGGGCCCGCGGTTCGCTGATgcggccgc

Figure S1 Scheme for cloning **(A)** sequence of shmiR inserts for CMTR1 **(B)** and for CMTR2 **(C)**. **(A)** sh neg plasmid was modified as described in *Material and Methods* section to generate sh CMTR1, sh CMTR2 and sh CMTR1+2 constructs. All four were used to obtain stable cell lines in HEK 293 Flp-In T-REx background. **(B,C)** Blue, green, red, and violet letters indicate BshTI, NheI, BspTI, and NotI restriction sites, utilized in the cloning procedure. Grey and black background indicate 5' and 3' miR flanking regions, respectively; red backgrounds correspond to 21-nt-long antisense target sequences (mature miRNA sequences); magenta backgrounds correspond to nucleotides 1-8 and 11-21 of the respective sense target sequences; green backgrounds represent a 19 nt-long sequence derived from endogenous murine miR-155 with segment able to form a loop within shmiR structure.

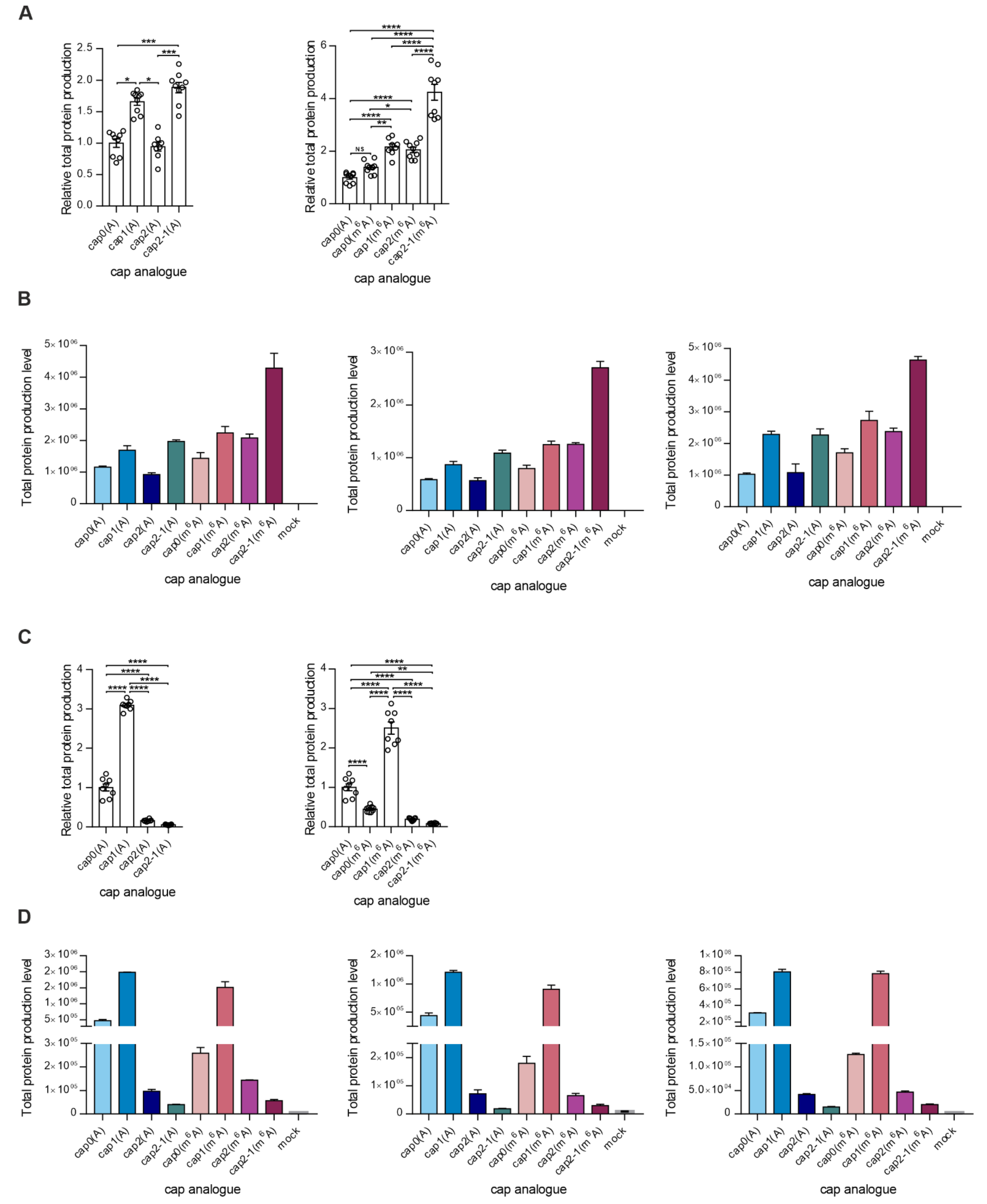

Figure S2 Total protein production levels after 72 h measured in the medium from culture of **(A,B)** A549 and **(C,D)** JAWS II cells transfected with IVT mRNAs encoding *Gaussia* luciferase bearing various cap analogues at their 5' ends. **(A)** and **(C)** Relative total protein production levels presented as in Figure 2A,B and Figure 2C,D, respectively, with additional single data point presentation. In **(B)** and **(D)** three independent biological replicates are presented, each consists of three independent transfections. Bars represent mean value ± SEM.

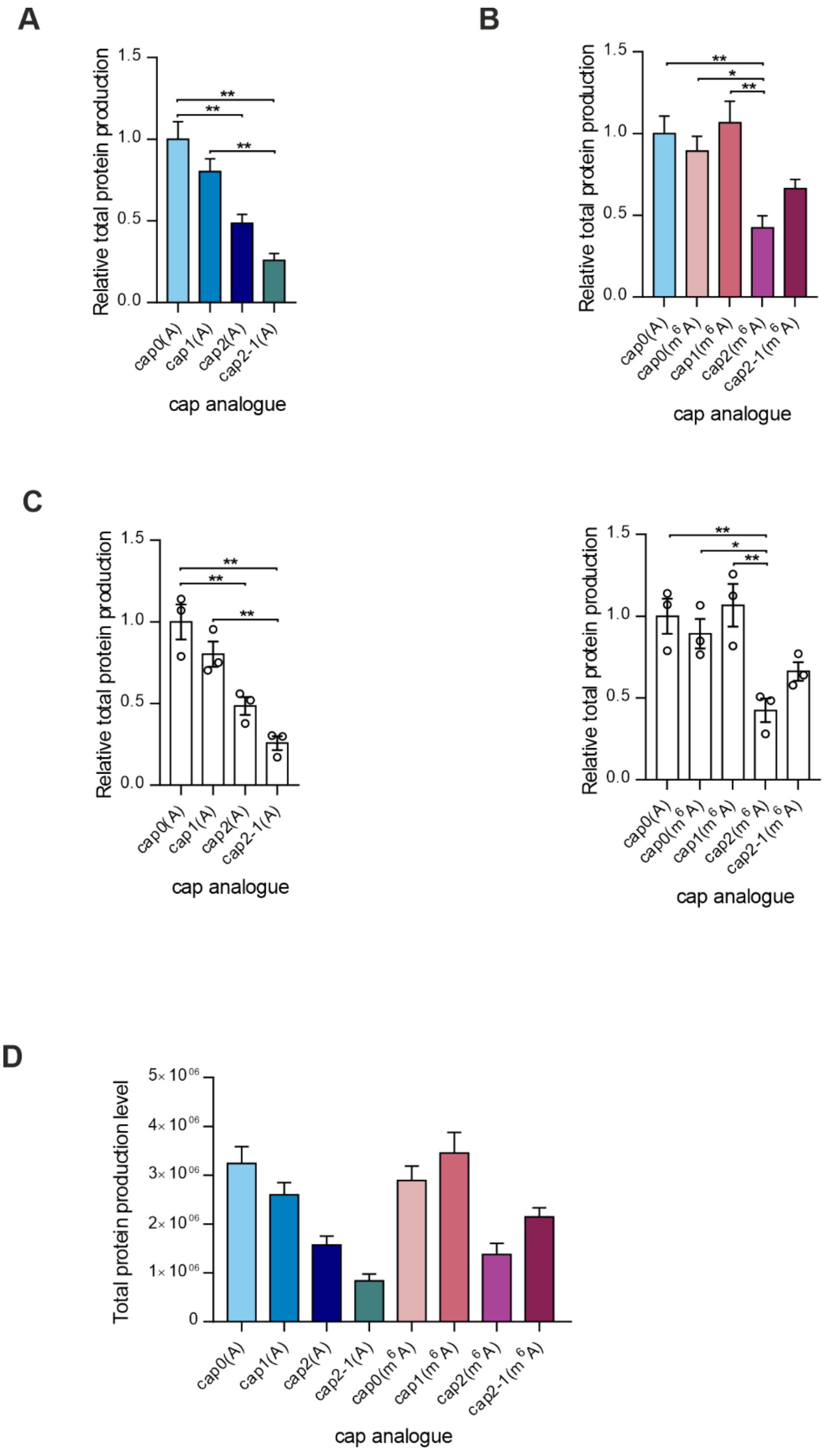

Figure S3 Protein production levels after 72 h measured in the medium from culture of THP-1 cells transfected with IVT mRNAs encoding *Gaussia* luciferase bearing various cap analogues at their 5' ends. **(A-C)** Bars represent mean value ± SEM normalized to transcripts with cap0(A). Statistical significance: * $P < 0.05$, ** $P < 0.01$, (one-way ANOVA with Turkey's multiple comparisons test). **(C)** Relative total protein productions level from **(A,B)** with additional single data point presentation. **(D)** Total protein production level used to calculate values on **(A,B)**.

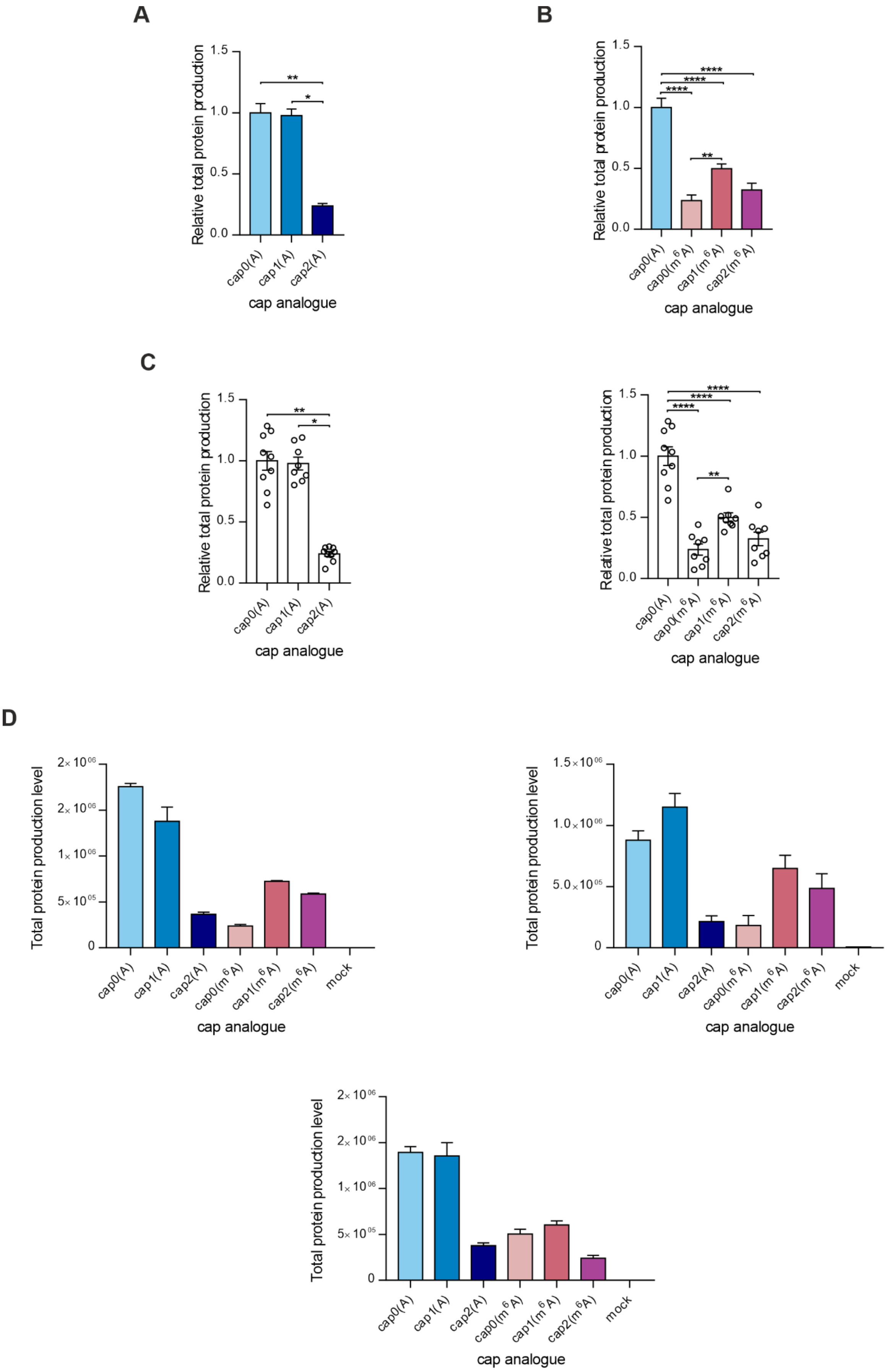

Figure S4 Protein production levels after 72 h measured in the medium from culture of 3T3-L1 cells transfected with IVT mRNAs encoding *Gaussia* luciferase bearing various cap analogues at their 5' ends. **(A-C)** Bars represent mean value ± SEM normalized to transcripts with cap0(A). Statistical significance: NS – not significant, * $P < 0.05$, ** $P < 0.01$, *** $P < 0.001$, **** $P < 0.0001$ (one-way ANOVA with Turkey's multiple comparisons test). **(C)** Relative total protein productions level from **(A,B)** with additional single data point presentation. **(D)** Three independent biological replicates are presented, each consists of three independent transfections. Bars represent mean value ± SEM.

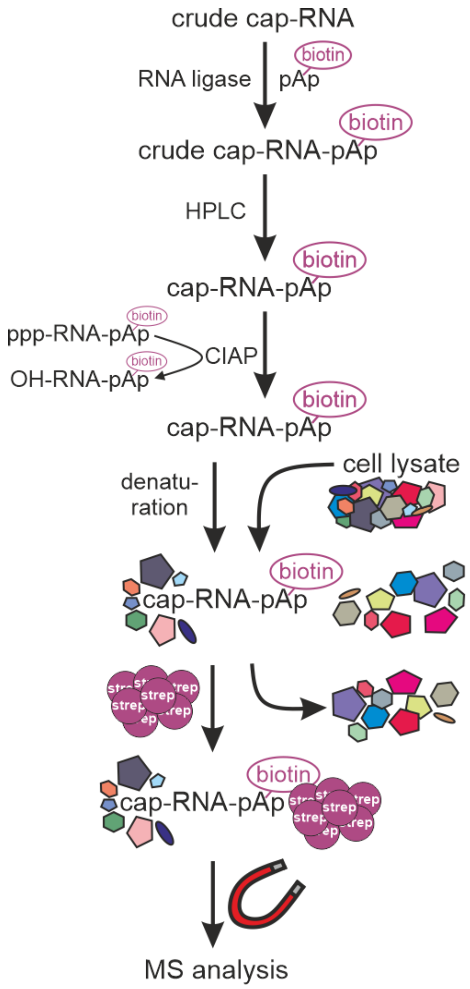

Figure S5 Scheme of affinity purification approach used to identify proteins binding to differently capped transcripts in A549 and JAWS II cell lysates.

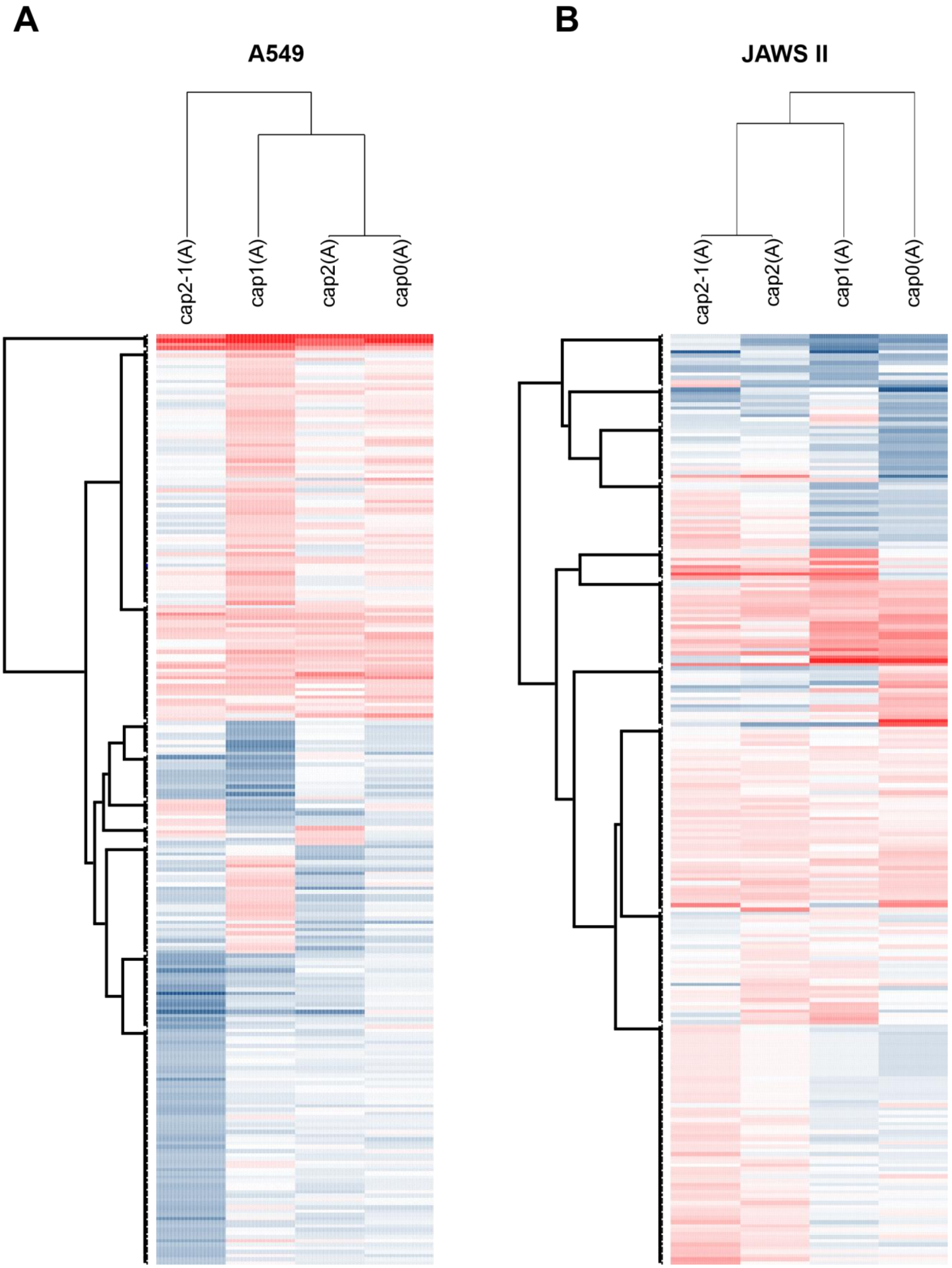

Figure S6 Heatmap showing hierarchical clustering of proteins performed on log2 ratio values using Euclidean distances generated in Perseus software (1). Ratio of any protein identified and quantified was calculated in comparison to the average level in the mock samples normalized to “1.0”.

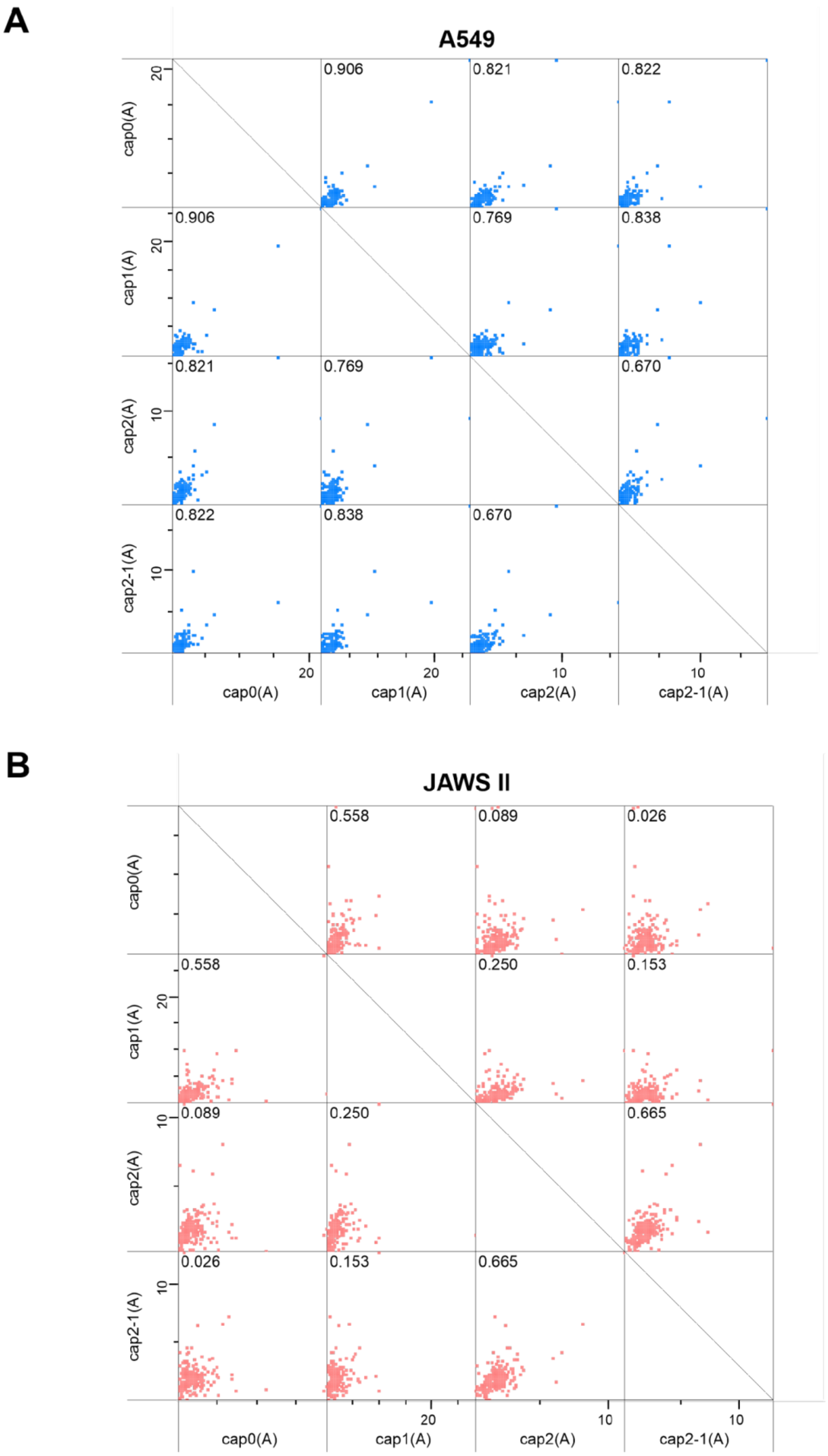

Figure S7 Correlation between identified interactomes for differently capped RNA for **(A)** A549 and **(B)** JAWS II cells. Pearson correlation coefficient for each comparison is presented in the left graph upper corner.

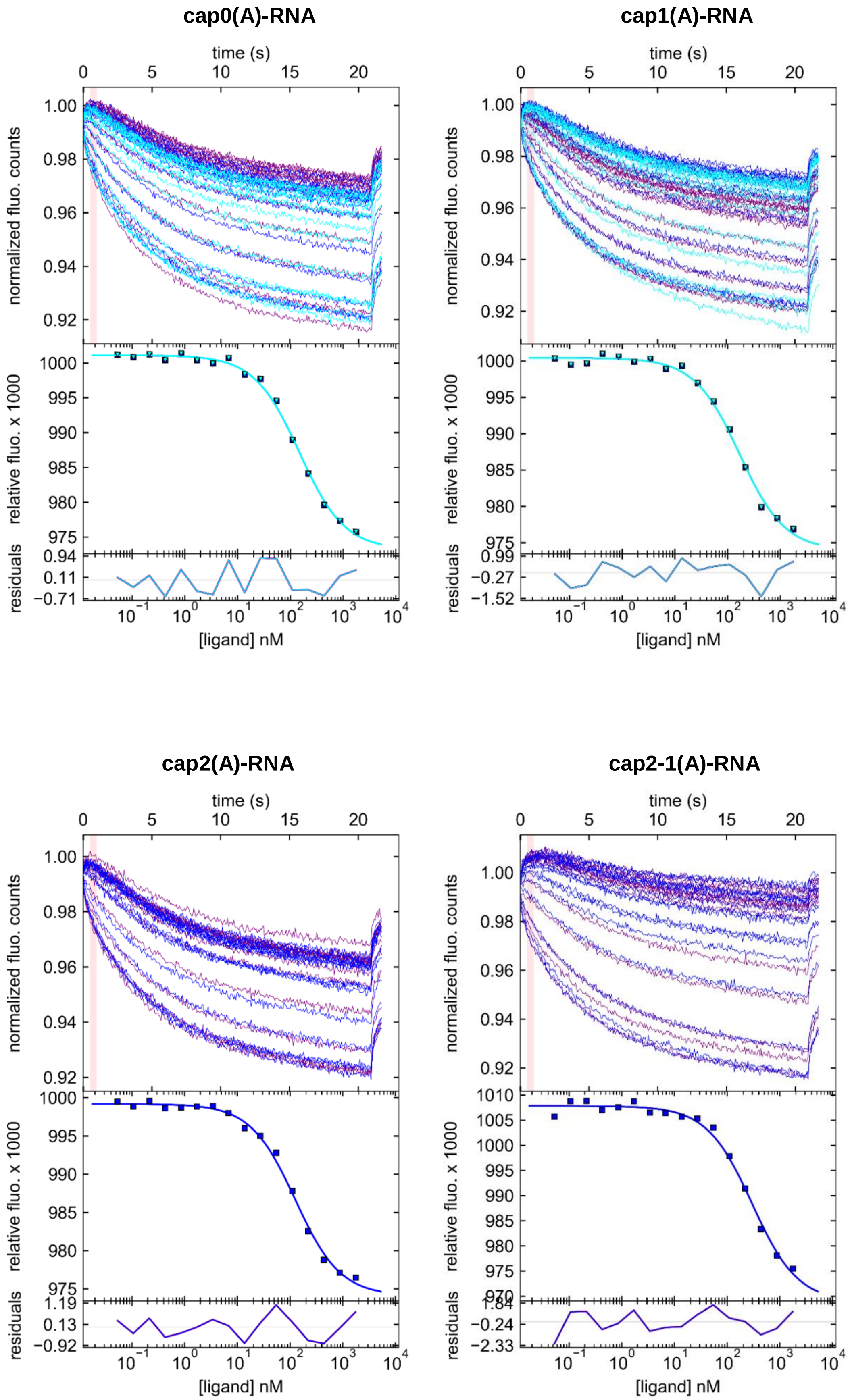

cap0(A)-RNA
cap1(A)-RNA
cap2(A)-RNA
cap2-1(A)-RNA
time (s)
normalized fluo. counts
relative fluo. x 1000
residuals
[ligand] nM
0.94
0.11
−0.71
0.99
−0.27
−1.52
1.19
0.13
−0.92
1.84
−0.24
−2.33

cap0(m^6A)-RNA

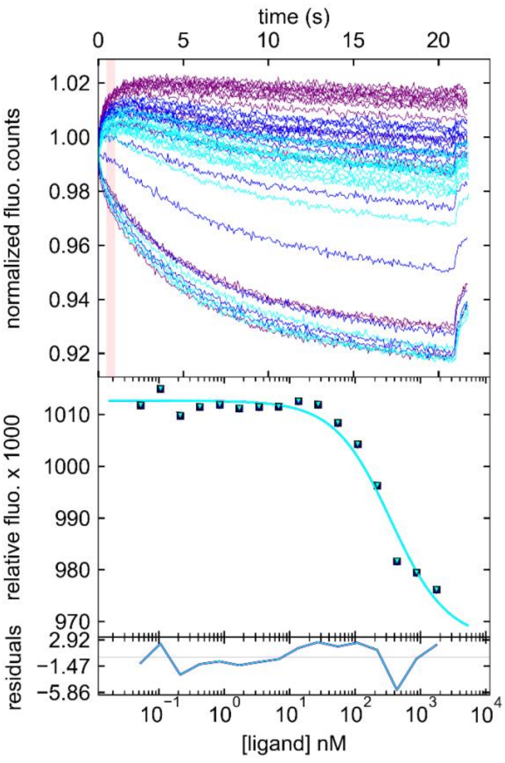

cap1(m^6A)-RNA

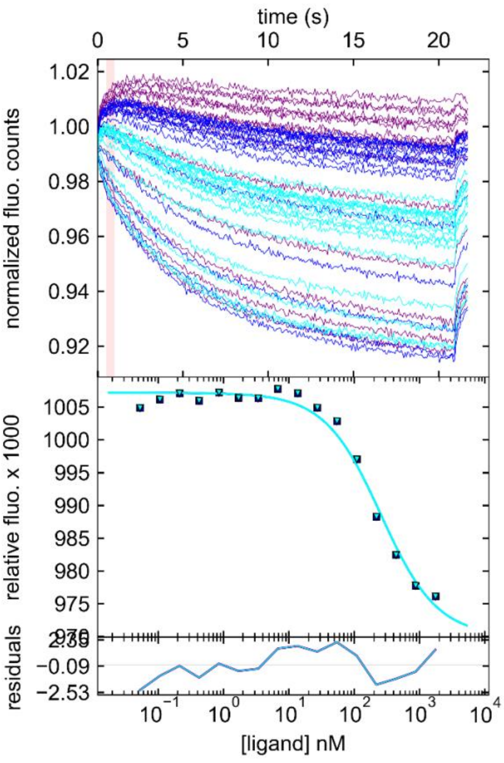

cap2(m^6A)-RNA

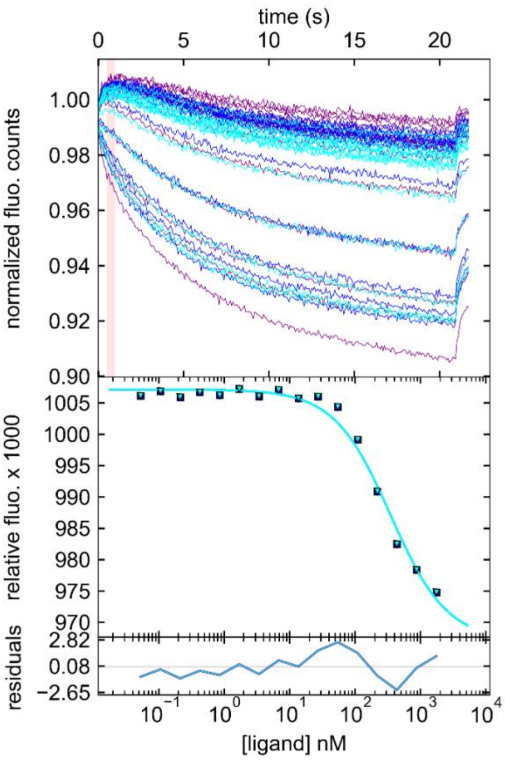

cap2-1(m^6A)-RNA

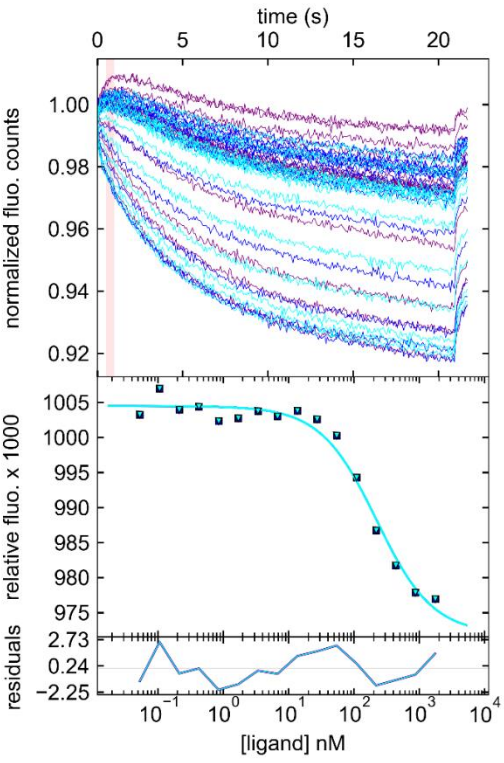

ppp-RNA

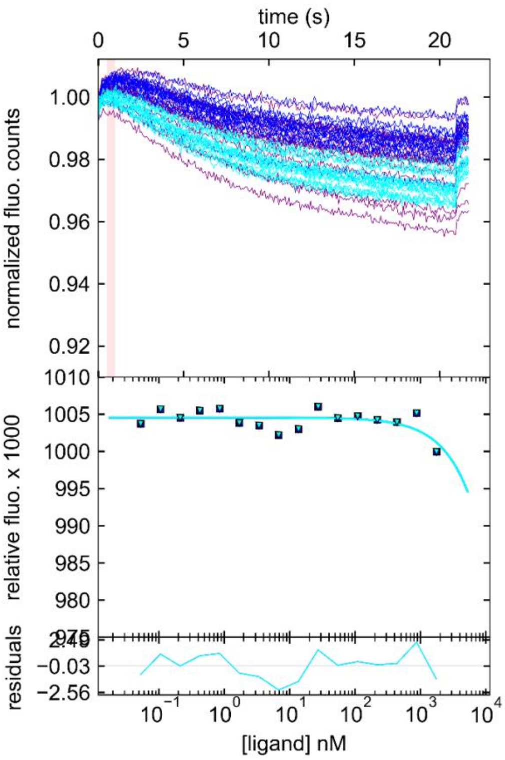

Figure S8 Determination of murine eIF4E-capped RNA complexes dissociation constants by microscale thermophoresis (MST) competition assay. Representative MST curves and competitive binding curves obtained for each RNA in the experiment are presented.

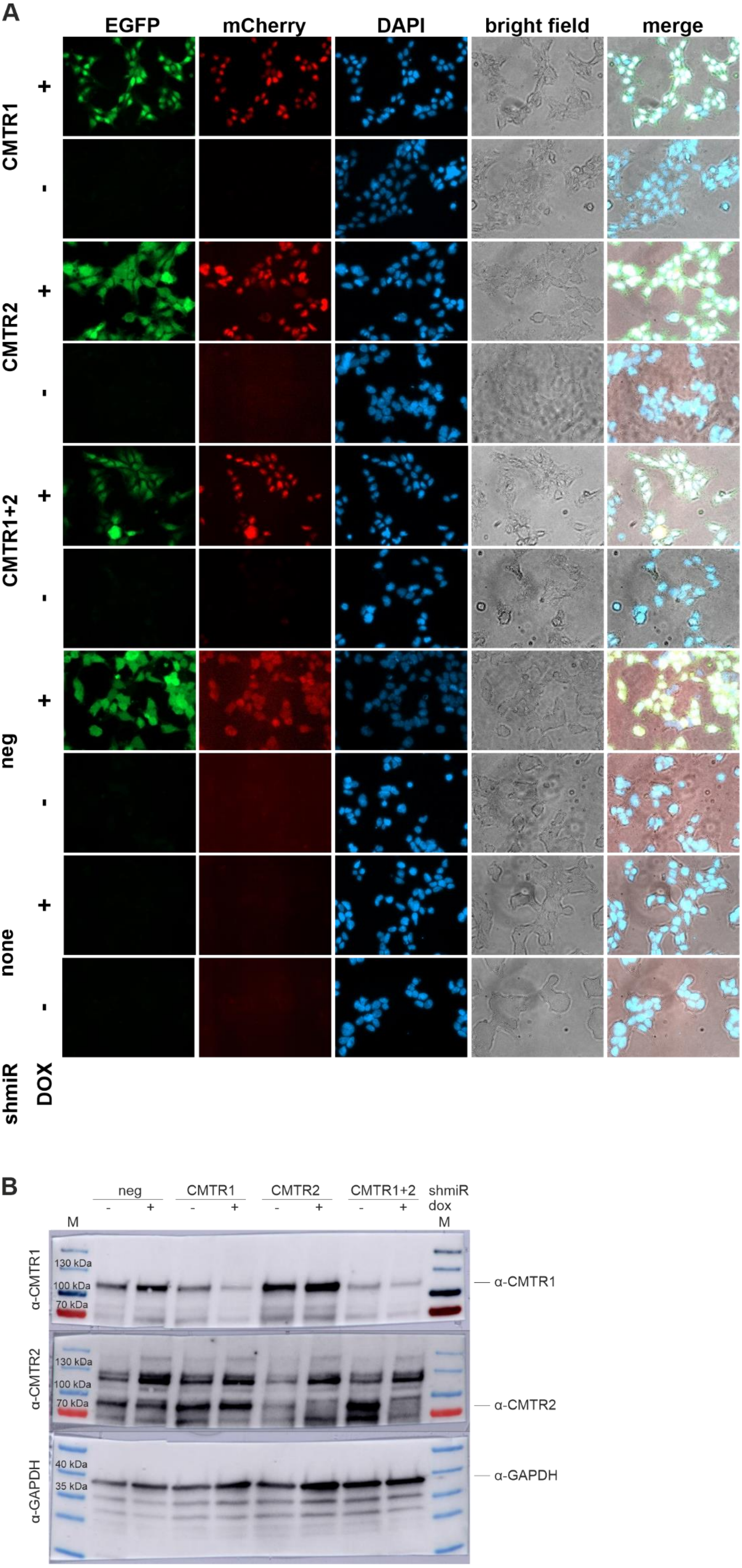
A
EGFP
mCherry
DAPI
bright field
merge
CMTR1
CMTR2
CMTR1+2
neg
none
shmiR
DOX
B
neg
CMTR1
CMTR2
CMTR1+2
shmiR
dox
M
130 kDa
100 kDa
70 kDa
40 kDa
35 kDa
α-CMTR1
α-CMTR2
α-GAPDH

Figure S9 Verification of stable cell lines generated in HEK 293 Flp-In T-REx background via **(A)** microscopic analysis and **(B)** western blot. **(A)** Expression of EGFP and mCherry fluorescent proteins was observed in constructed stable cell lines cultured with doxycycline (+DOX). Cells cultured without doxycycline (-DOX), as well as non-modified parental HEK 293 Flp-In T-REx cells (-/+DOX), served as controls. EGPF and mCherry are two reporters introduced to genomes of stable cell lines used in the study, under bidirectional promoter which expression is induced upon doxycycline treatment. **(B)** Efficient depletion of CMTR1, CMTR2 or both CMTR1 and 2 was observed in cells expressing respective shmiRs upon doxycycline induction. GAPDH served as loading control.

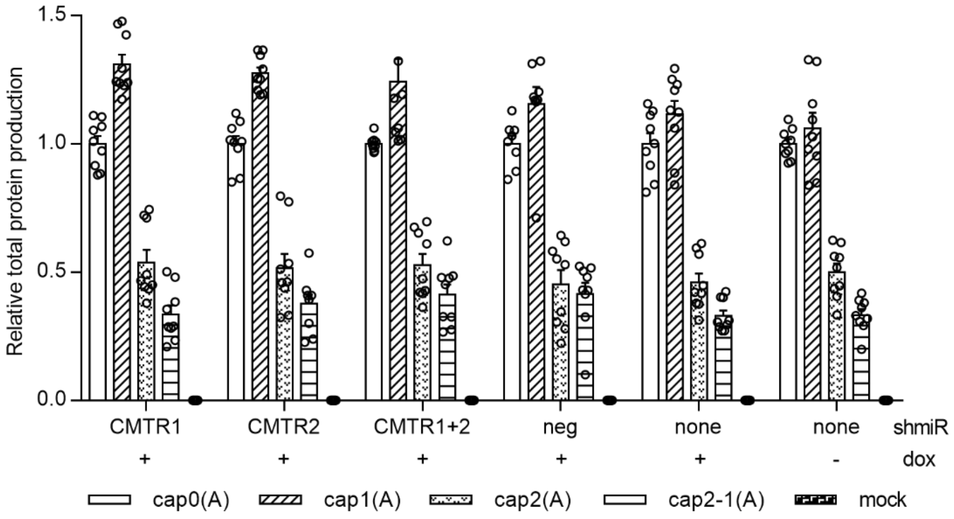

Figure S10 Relative total protein production levels after 72 h measured in the medium from cultures of different 293 Flp-In T-REx cell lines, transfected with IVT mRNAs encoding Gaussia luciferase bearing various cap analogues at their 5' ends. The graph couples results from Figure 4C for stable cell lines cultured with doxycycline with results for non-modified 293 Flp-In T-REx cells (no shmiRs) cultured with or without doxycycline, with additional single data point presentation. Three independent biological replicates are presented, each consisting of three independent transfections. Bars represent mean value ± SEM.

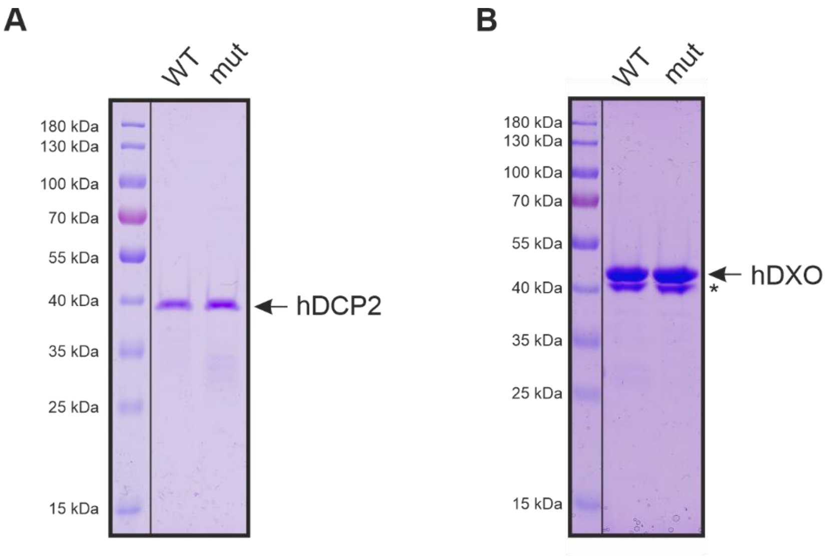

Figure S11 hDCP2 and hDXO purification. SDS-PAGE analysis of the purified recombinant hDCP2 **(A)** and hDXO **(B)** wild-type and catalytic mutant variants. Sizes of the molecular weight marker bands are indicated on the left. Full-length proteins are marked with arrows on the right. Asterisk in **(B)** shows position of the protein co-migrating with hDXO after two rounds of affinity chromatography and gel filtration, likely representing partial degradation product.

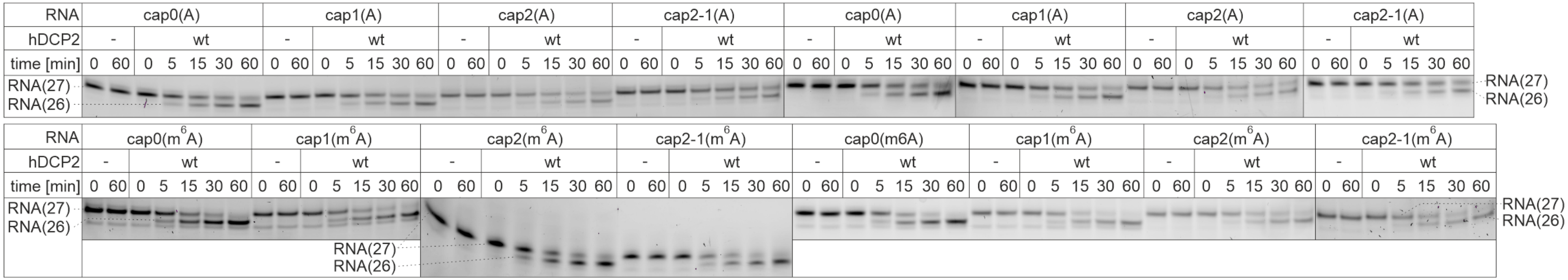

Figure S12 Susceptibility of short transcripts to recombinant hDCP2 action. Short capped RNAs were subjected to treatment with wild type hDCP2 for 60 min. Reactions without enzyme served as controls. Aliquots from indicated time points were resolved in polyacrylamide gel and bands corresponding to capped (27-nt long) and to RNAs decapped by hDCP2 action (26-nt long). Two independent replicates are presented. One more replicate of this experiment is shown in Figure 4A.

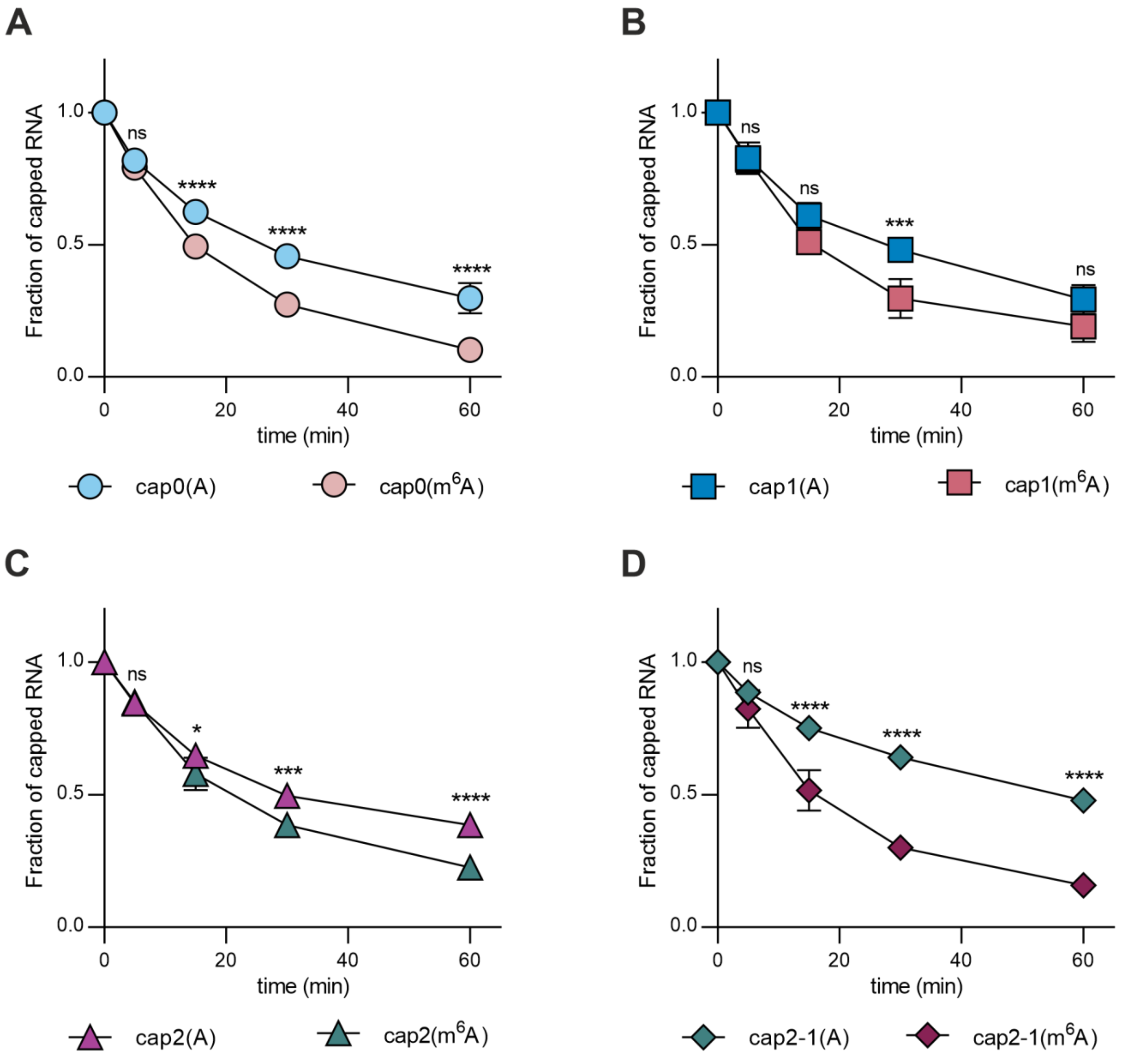

Figure S13 Presence of *N*6-methyladenosine as the first transcribed nucleotide increases susceptibility of RNA to decapping by hDCP2. **(A-D)** Quantitative results for all studied RNAs. The fraction of capped RNA remaining in the total RNA was plotted as a function of time. Data points represent mean values ± SD from triplicated experiments. Statistical significance: ns – not significant, * $P < 0.05$, ** $P < 0.01$, *** $P < 0.001$, **** $P < 0.0001$ (two-way ANOVA with Sidak's multiple comparisons test). For some points, the error bars are shorter than the height of the symbol**.**

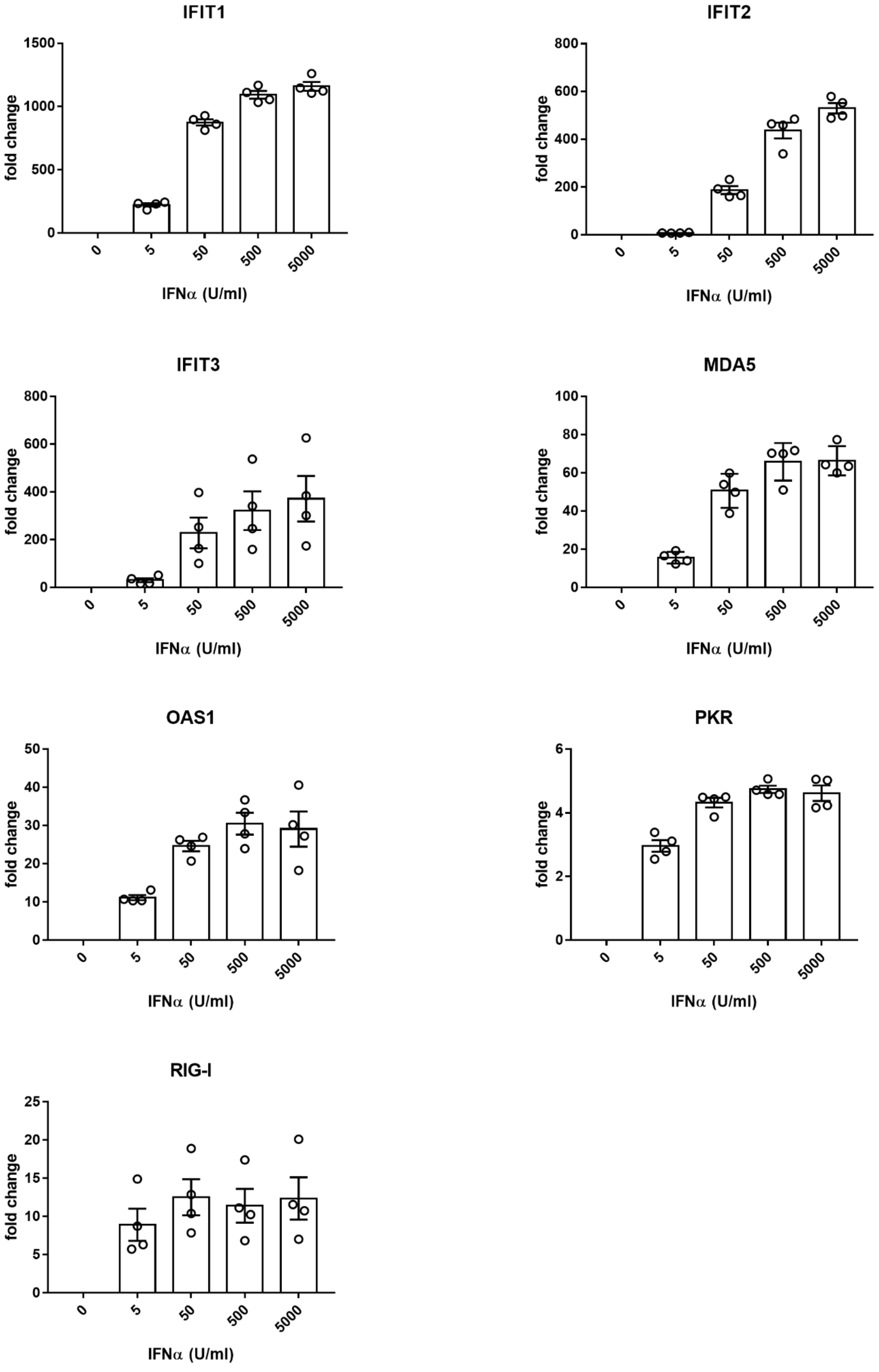

Figure S14 Changes in gene expression upon treatment with increasing concentration of IFNα for 5 h. mRNA expression analysis for the indicated genes was carried out using RT-qPCR. GAPDH housekeeping gene was used for normalization. Bars represent the mean value of mRNA level change (fold change) ± SEM from four independent biological replicates, each independent biological replicate consists of a single transfection reaction. Each point represents data from one independent biological replicate.

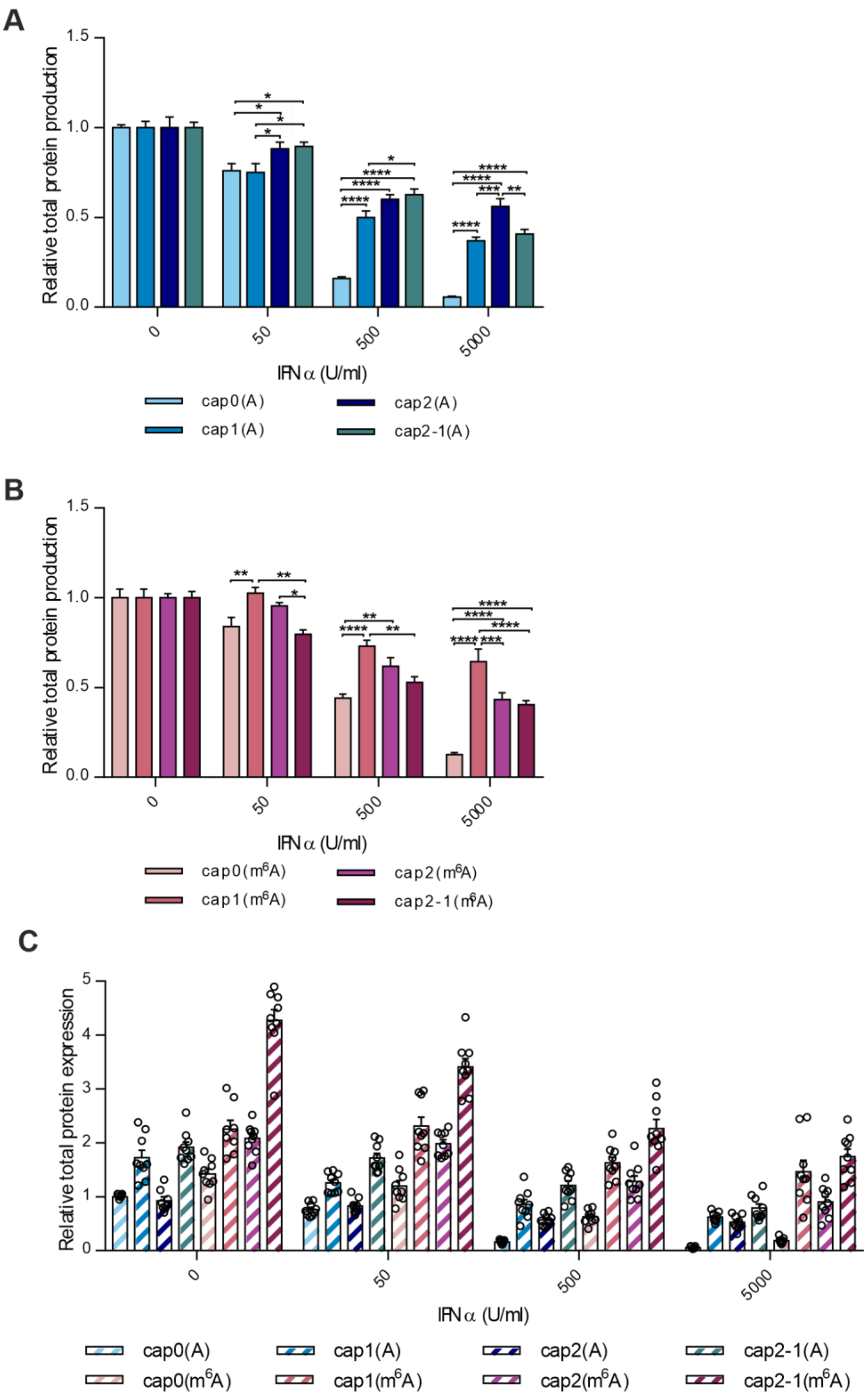

Figure S15 Changes in relative protein production in response to increasing concentration of IFNα in A549 cells. Relative protein production levels over 72 hours after 5 hour IFNα pre-treatment in A549 cells. **(A,B)** Bars for each transcript represent mean value ± SEM normalized to protein production in mock treated (0 U/ml IFNα) cells (Protein production level for each capped mRNA in mock treated cells was set as 1.0). Statistical significance: * P < 0.05, ** P < 0.01, *** P < 0.001, **** P < 0.0001 (one-way ANOVA with Turkey's multiple comparisons test); **(C)** Combined results from (A) and (B) with additional single data point presentation. Bars for each transcript represent mean value ± SEM normalized to protein production in mock treated cells transfected with mRNA bearing cap0(A).

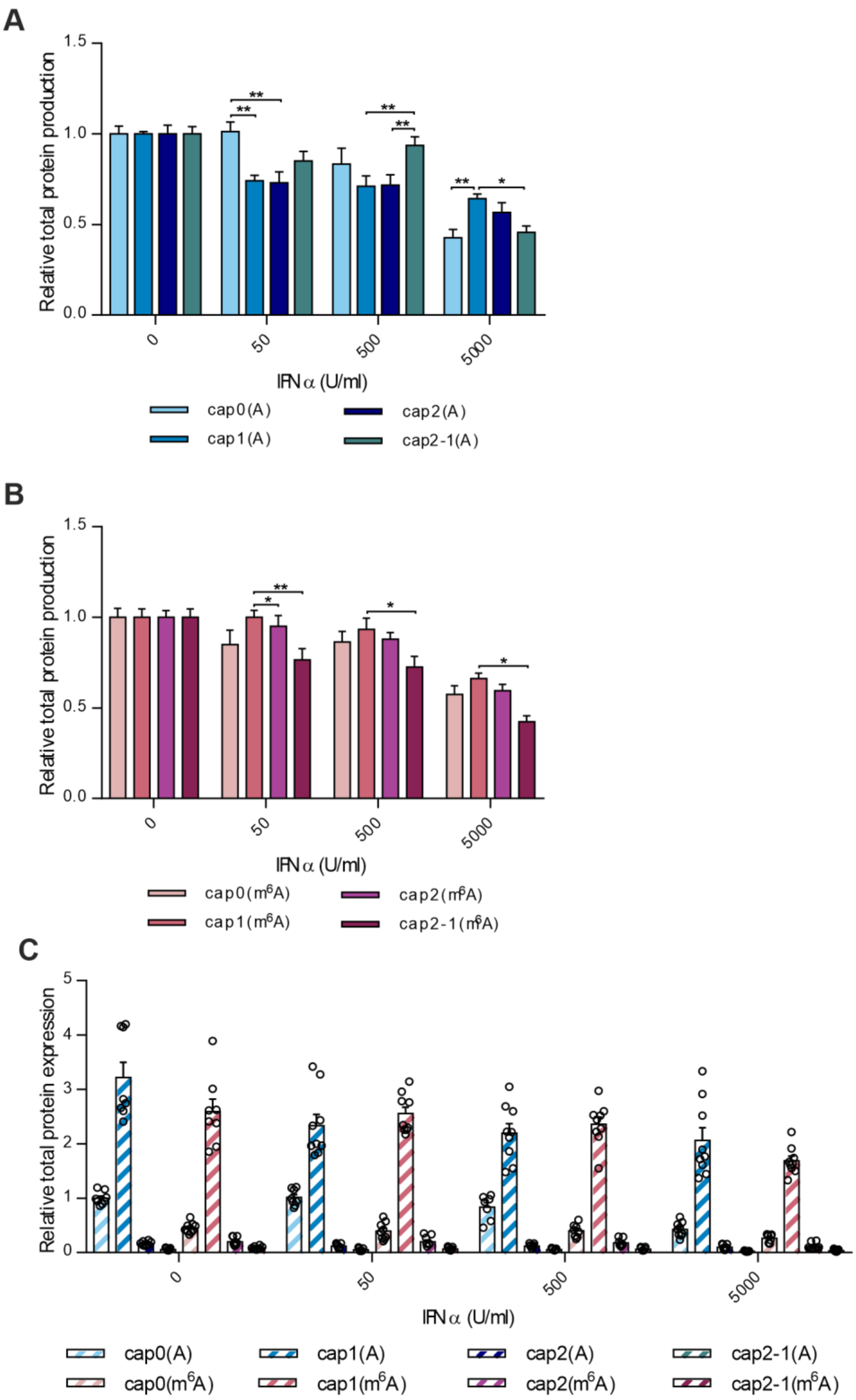

Figure S16 Changes in relative protein production in response to increasing concentration of IFNα in JAWS II cells. Relative protein production levels over 72 hours after 5 hour IFNα pre-treatment in JAWS II cells. **(A,B)** Bars for each transcript represent mean value ± SEM normalized to protein production in mock treated (0 U/ml IFNα) cells (Protein production level for each capped mRNA in mock treated cells was set as 1.0). Statistical significance: * $P < 0.05$, ** $P < 0.01$, *** $P < 0.001$, **** $P < 0.0001$ (one-way ANOVA with Turkey's multiple comparisons test); **(C)** Combined results from (A) and (B) with additional single data point presentation. Bars for each transcript represent mean value ± SEM normalized to protein production in mock treated cells transfected with mRNA bearing cap0(A).

Table S1. List of oligonucleotides used in the study

Oligonucleotide	Sequence	Purpose
IVTshF	CAGTAATACGACTCACTATAGGGGAAGCGGGCATGCGGCCAGCCATAGCCGATCA (2)	template for IVT of short RNA
IVTshR	TGATCGGCTATGGCTGGCCGCATGCCCGCTTCCCCTATAGTGAGTCGTATTACTG (2)	template for IVT of short RNA
DNAzyme	TGATCGGCTAGGCTAGCTACAACGAGGCTGGCCGC (2)	short RNA trimming
IFIT1_F	GATCAGCCATATTTCATTTTGAATC	qPCR
IFIT1_R	GAAAATTCTCTTCAGCTTTTCTGTG	qPCR
IFIT2_F	AAGAGGAAGATTTCTGAAGAGTGC	qPCR
IFIT2_R	TCTCCAAGGAATTCTTATTGTTCTC	qPCR
IFIT3_F	GAAGGAACTGGGCCGCCTGCTAAG	qPCR
IFIT3_R	GCCCTGGCCCATTTCCTCACTACC	qPCR
RIG-I_F	ATGTGCTCCTACAGGTTGTGG	qPCR
RIG-I_R	ACACTGGGATCTGATTCGCAA	qPCR
MDA5_F	GAGTCAAAGCCCACCATCTGA	qPCR
MDA5_R	CAGACCTTCTTCTGCCACTGT	qPCR
PKR_F	CAGACCCCTTGTCCAGACTTC	qPCR
PKR_R	CGGGAAGGGTAAGATGGCTTT	qPCR
OAS1_F	TCCTCAGTCCTCTCACCACTT	qPCR
OAS1_R	GAGCCTGGACCTCAAACTTCA	qPCR
GAPDH_F	ACCCACTCCTCCACCTTTGAC	qPCR
GAPDH_R	TGTTGCTGTAGCCAAATTCGTT	qPCR
hDXOfor	actggtggacagcaaatgggtcgc**ggatccATGGATCCCAGGGGGACCAAG**	cloning of full-length hDXO wt and mut into pET28-N-6xHis-TEV
hDXOrev	tcagtggtggtggtggtggtg**ctcgag*TTA*TTTGGGAGAGGGAGTCTTGG**	cloning of full-length hDXO wt and mut into pET28-N-6xHis-TEV
hDXOmutF	**AGCCACCCTCTGCTCTTCTCAGGGGCGGTAGCATGCACAGACCCCCAAGC**	cloning of full-length hDXO mut into pET28-N-6xHis-TEV
hDXOmutR	**GCTTGGGGGTCTGTGCATGCTACCGCCCCTGAGAAGAGCAGAGGGTGGCT**	cloning of full-length hDXO mut into pET28-N-6xHis-TEV
hDCP2F	GACGCGGCCGCCCCTTCACCTCACTTCATCAAAGAATTCTGCTG	cloning of 1-350 aa hDCP2 mut into pET28-N-6xHis-TEV
hDCP2R	GACGGATCCCTTATGGAGACCAAACGGGTGGAG	cloning of 1-350 aa hDCP2 mut into pET28-N-6xHis-TEV

Table S2 List of plasmids used in the study

Plasmid	Genotype	Source
phDXOwt	[pET28M N-6xHis-TEV] hDXO wt	this work
phDXOmut	[pET28M N-6xHis-TEV] hDXO mut (E234A D236A)	this work
phDCP2wt	[pET28M N-6xHis-SUMOTag] hDCP2$^{1\text{-}350}$wt	gift from Megerditch Kiledjian
phDCP2mut	[pET28M N-6xHis-SUMOTag] hDCP2$^{1\text{-}350}$mut (E147Q E148Q)	this work
pKK-RNAi-nucCHERRYmiR-EGFP-TEV (sh neg)	[BI-16 constructed based on pcDNA5/FRT/TO] nucCHERRY-shmiRNAneg; EGFP-TEV	gift from Andrzej Dziembowski and Roman Szczesny, Addgene plasmid #105810
sh CMTR1	[BI-16] nucCHERRY; EGFP-TEV-shmiRCMTR1	this work
sh CMTR2	[BI-16] nucCHERRY-shmiRCMTR2; EGFP-TEV	this work
sh CMTR1+2	[BI-16] nucCHERRY-shmiRCMTR2; EGFP-TEV-shmiRCMTR1	this work

Table S3 HPLC profiles and HRMS spectra of synthetized compounds

	pA$_m$pG$_m$pG
Chemical structure	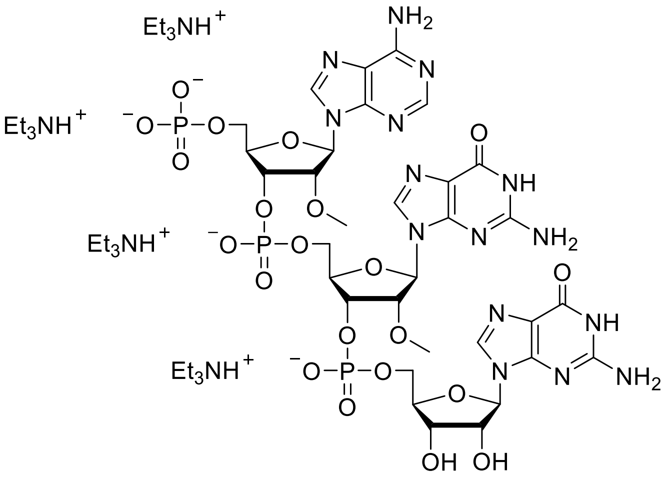
RP HPLC	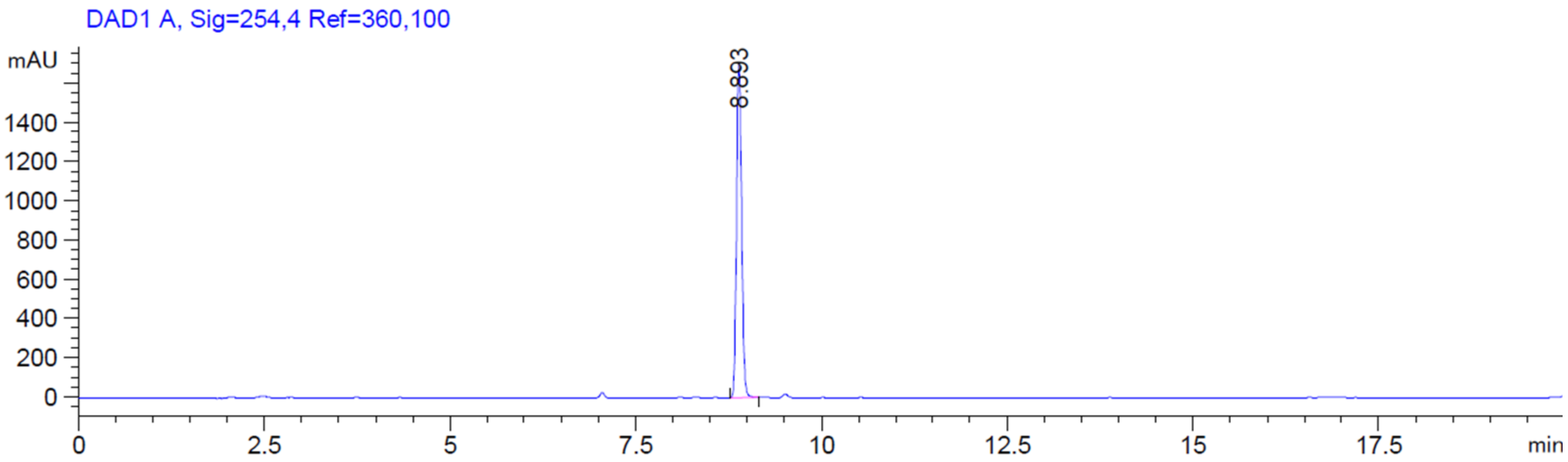

HR ESI(-) MS

Calc. $[M-H]^-$ $C_{32}H_{41}N_{15}O_{21}P_3^-$ 1064.18198

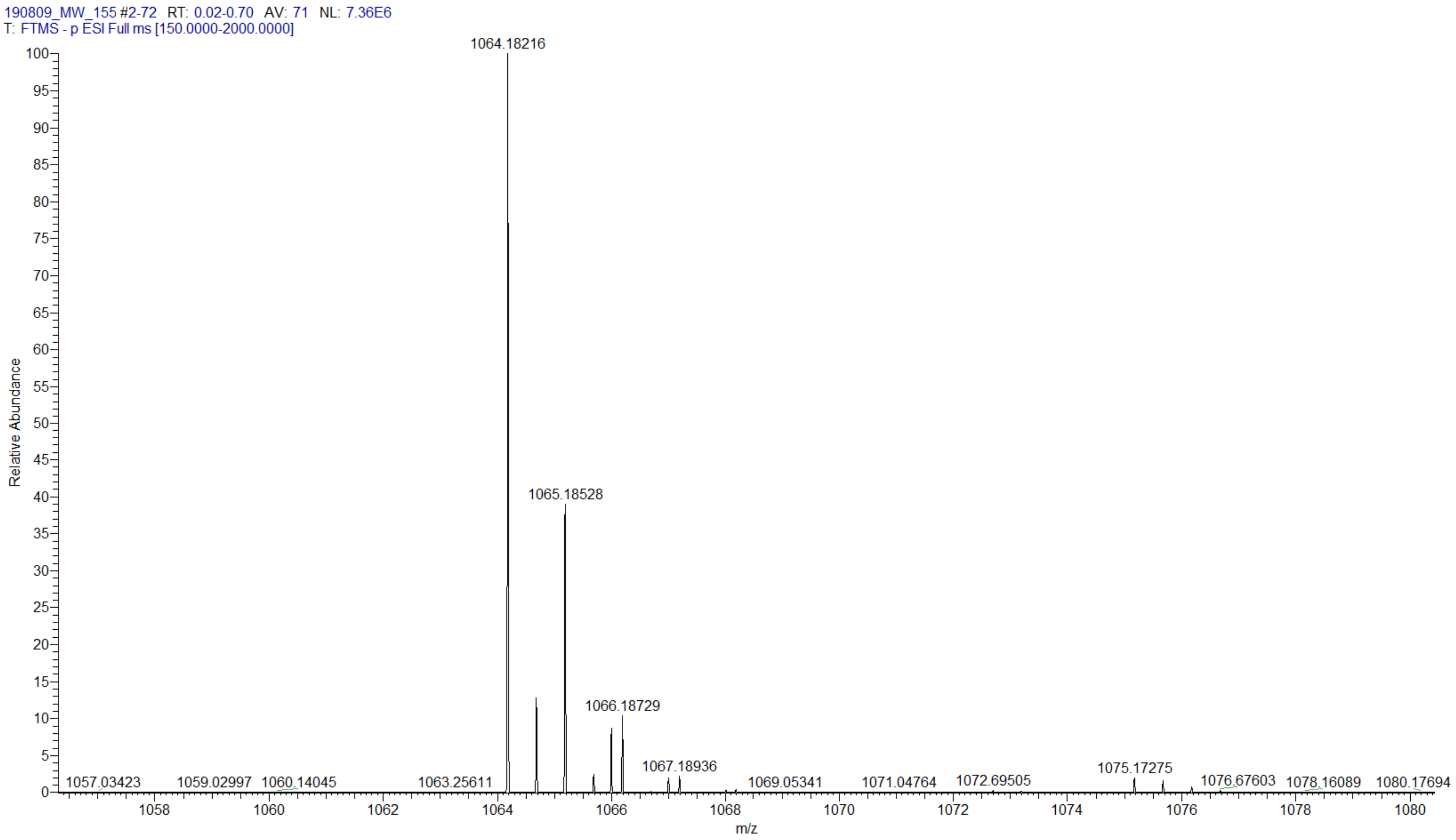

p(m^{6}A$_m$)pG$_m$pG	
**Chemical structure**	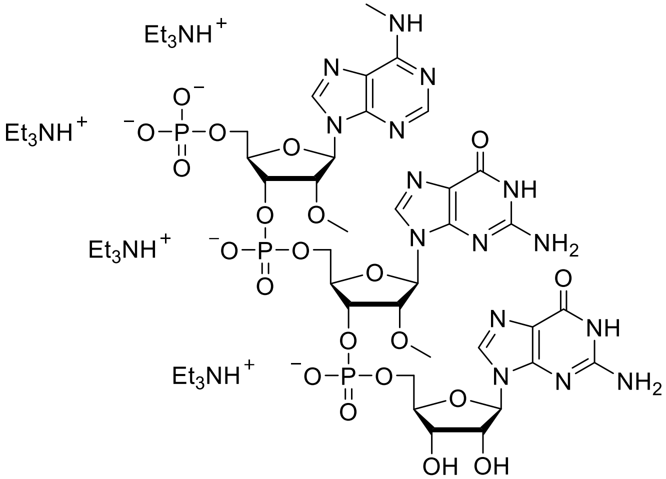
**RP HPLC**	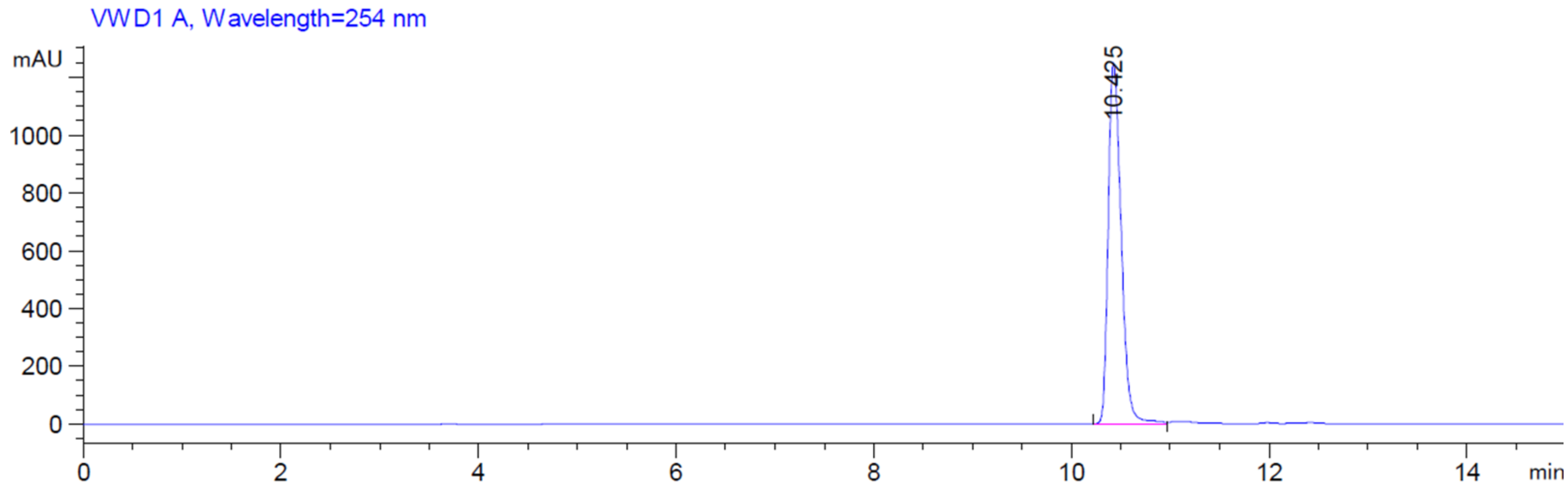

HR ESI(-) MS

Calc. [M-H]⁻ $C_{33}H_{43}N_{15}O_{21}P_3^-$ 1078.19763

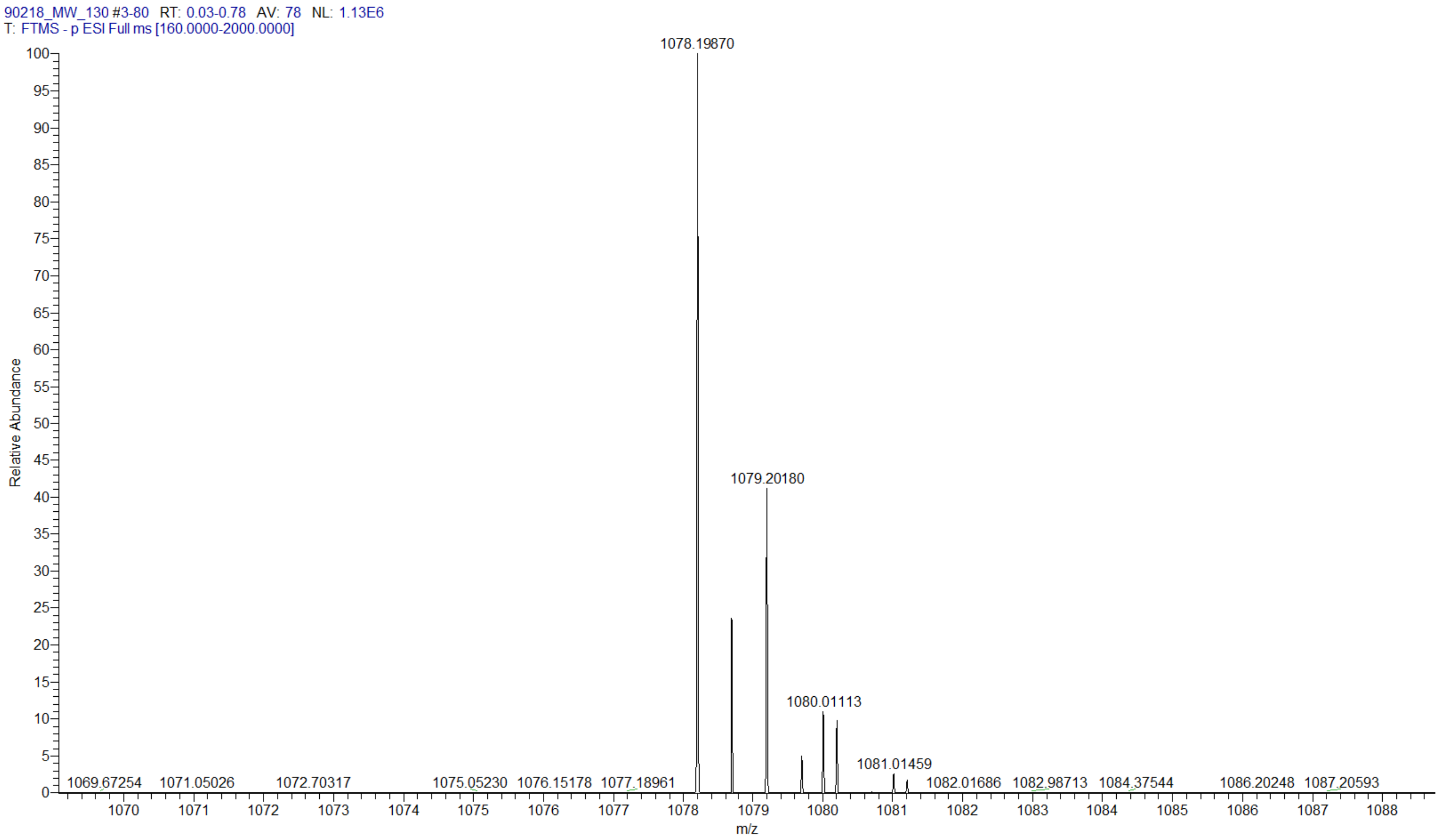

pApG$_m$pG

Chemical structure

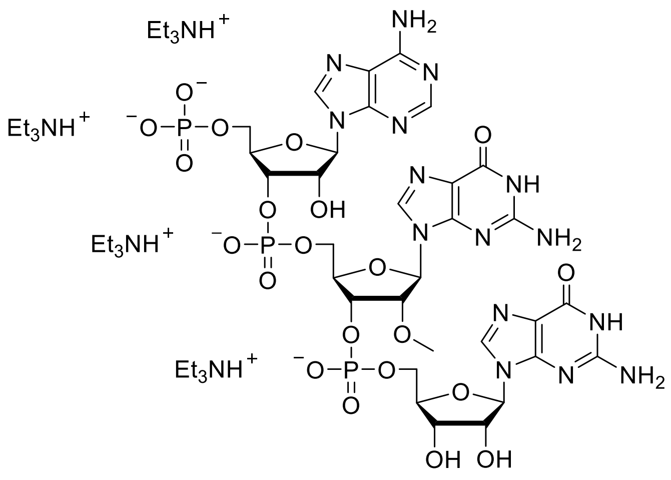

RP HPLC

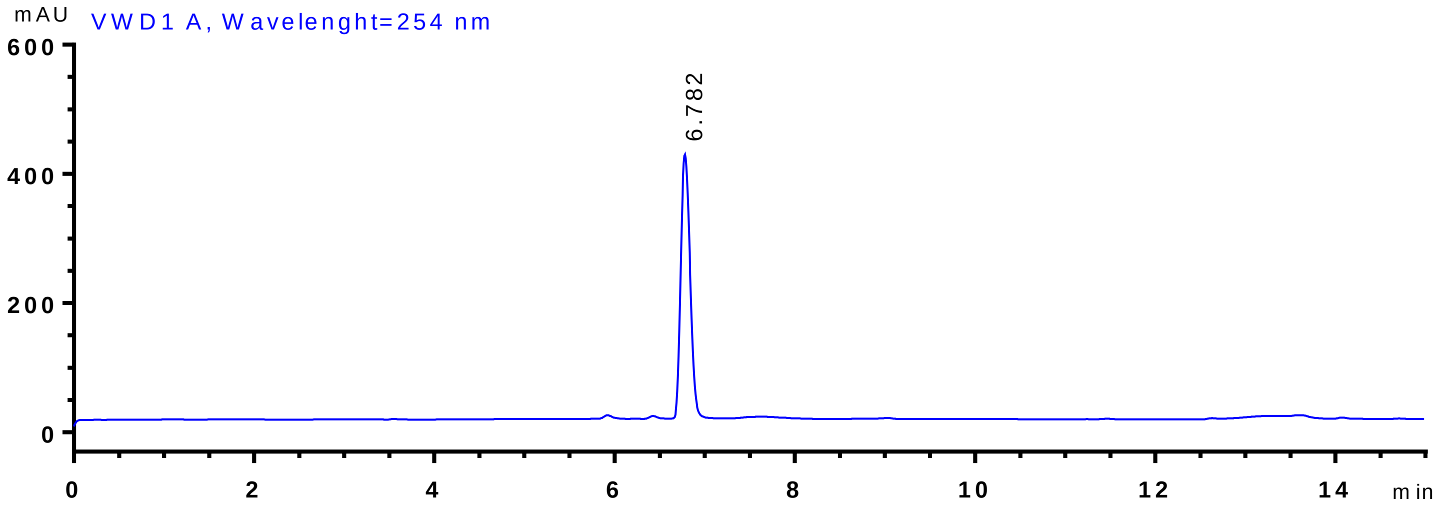

HR ESI(-) MS

Calc. [M-H]⁻ $C_{31}H_{39}N_{15}O_{21}P_3^-$ 1050.16633)

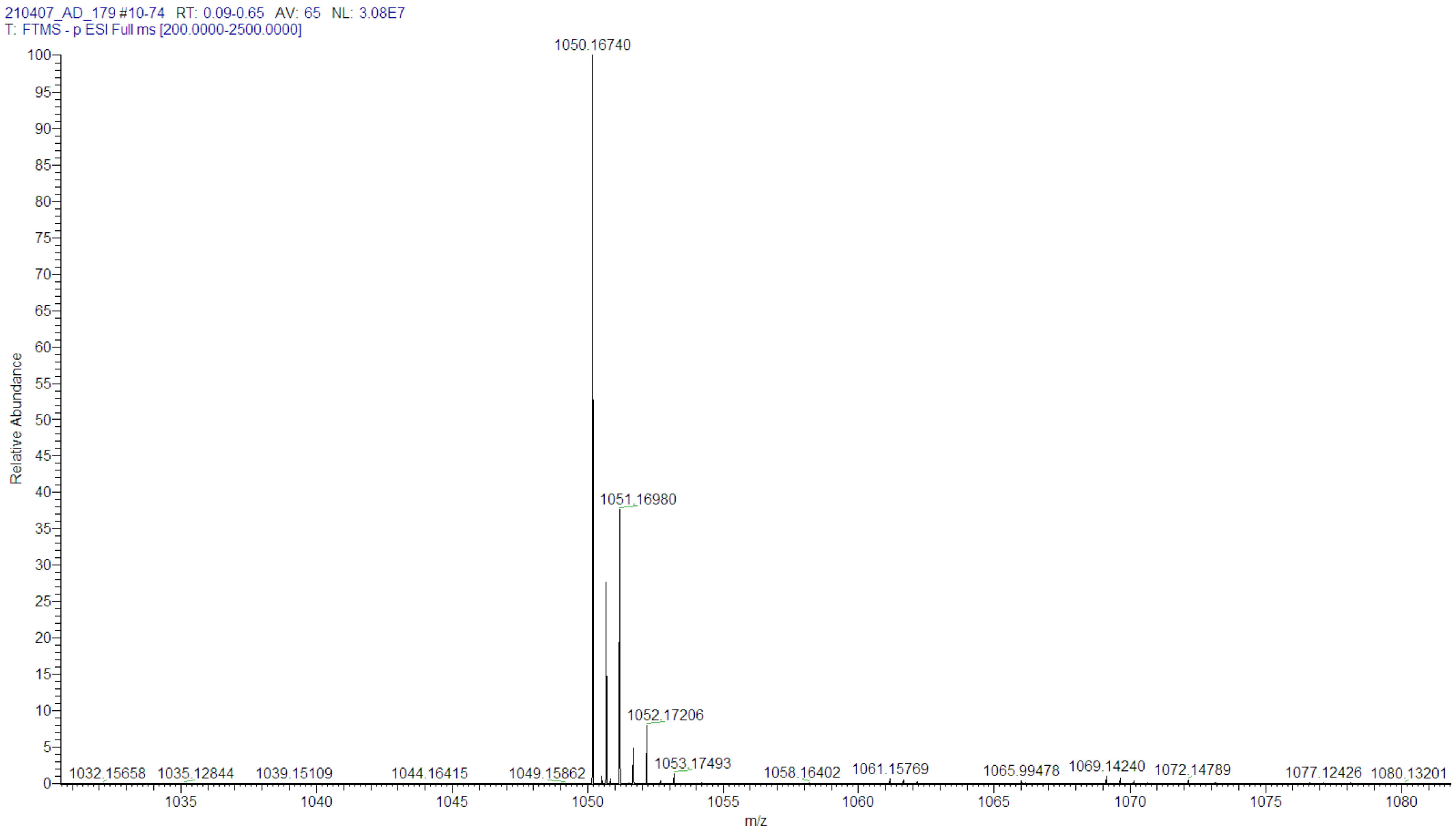

p(m^{6}A)pG$_m$pG

Chemical structure

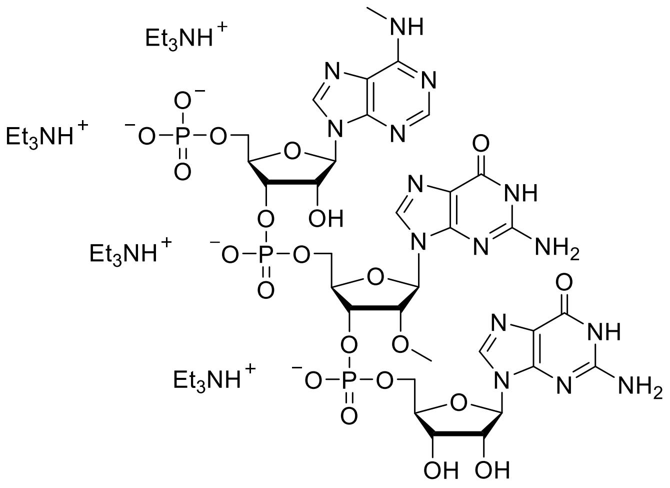

RP HPLC

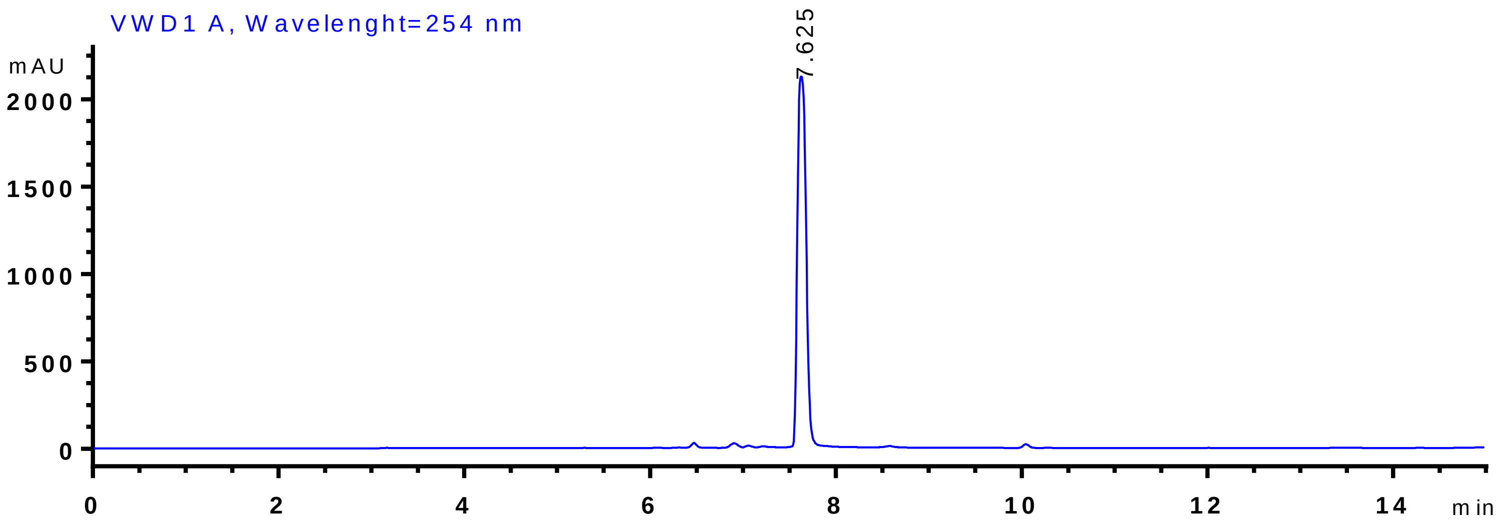

HR ESI(-) MS

Calc. [M-H]$^-$ $C_{32}H_{41}N_{15}O_{21}P_3^-$ 1064.18198

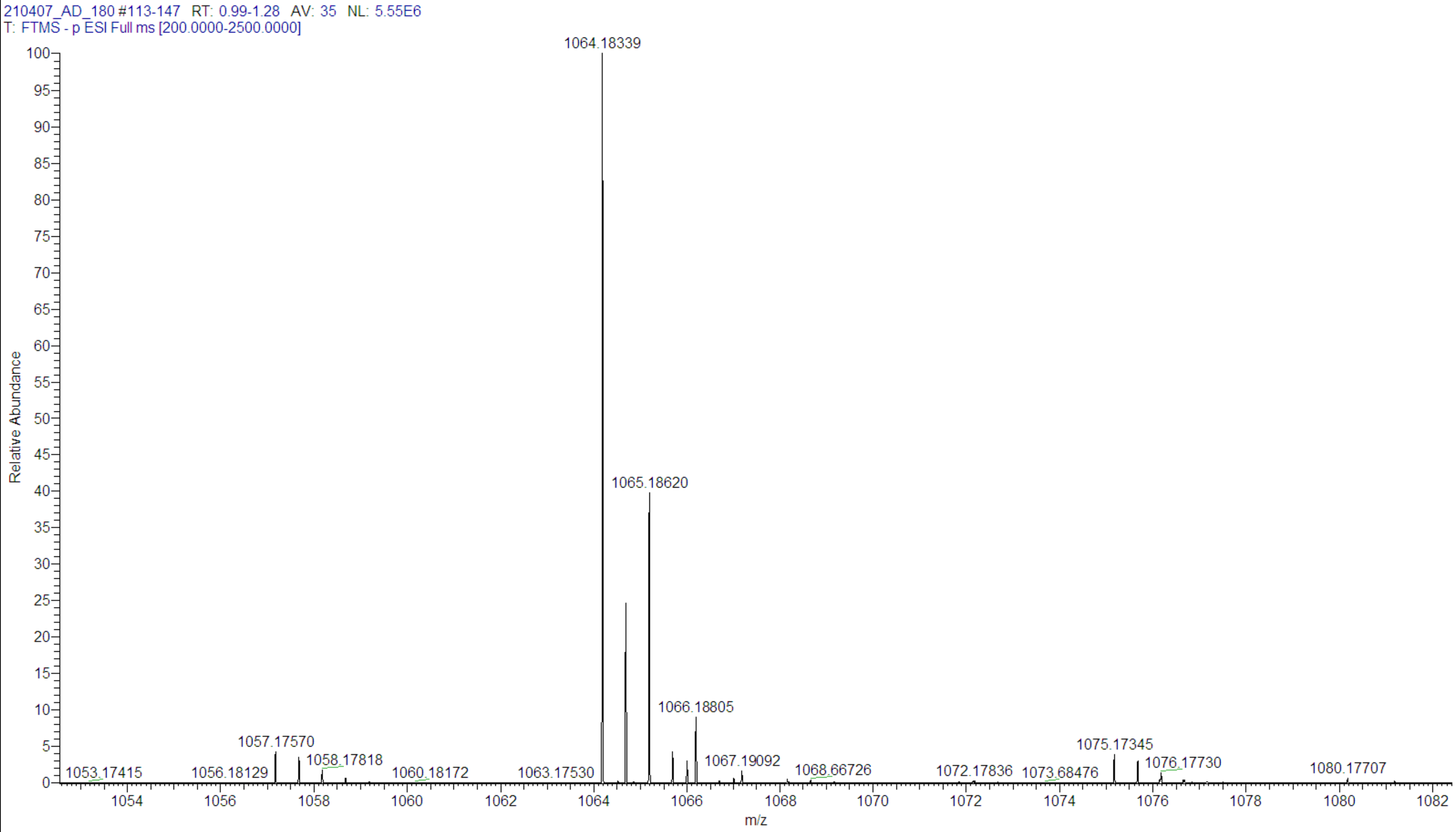

m^{7}GpppA$_m$pG$_m$pG

Chemical structure

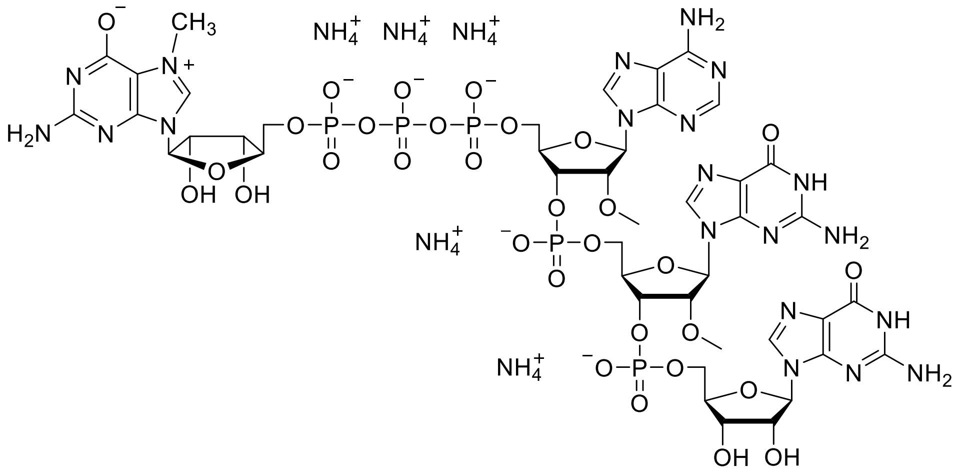

RP HPLC

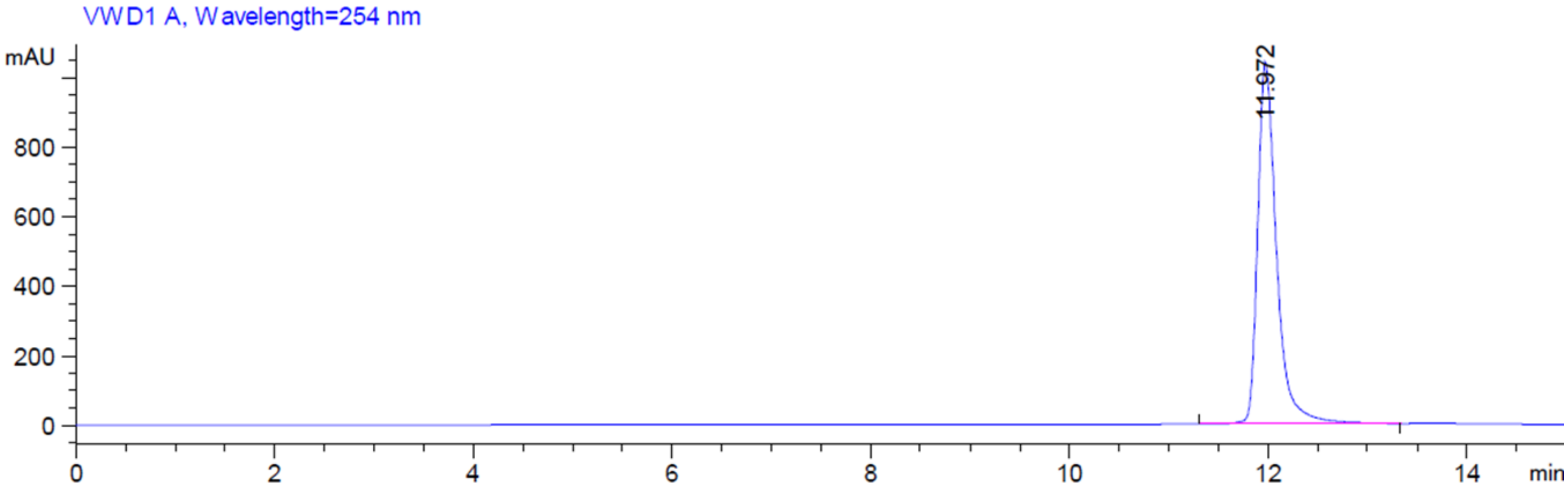

HR ESI(-) MS

Calc. [M-H]$^-$ $C_{43}H_{56}N_{20}O_{31}P_5^-$ 1503.21139)

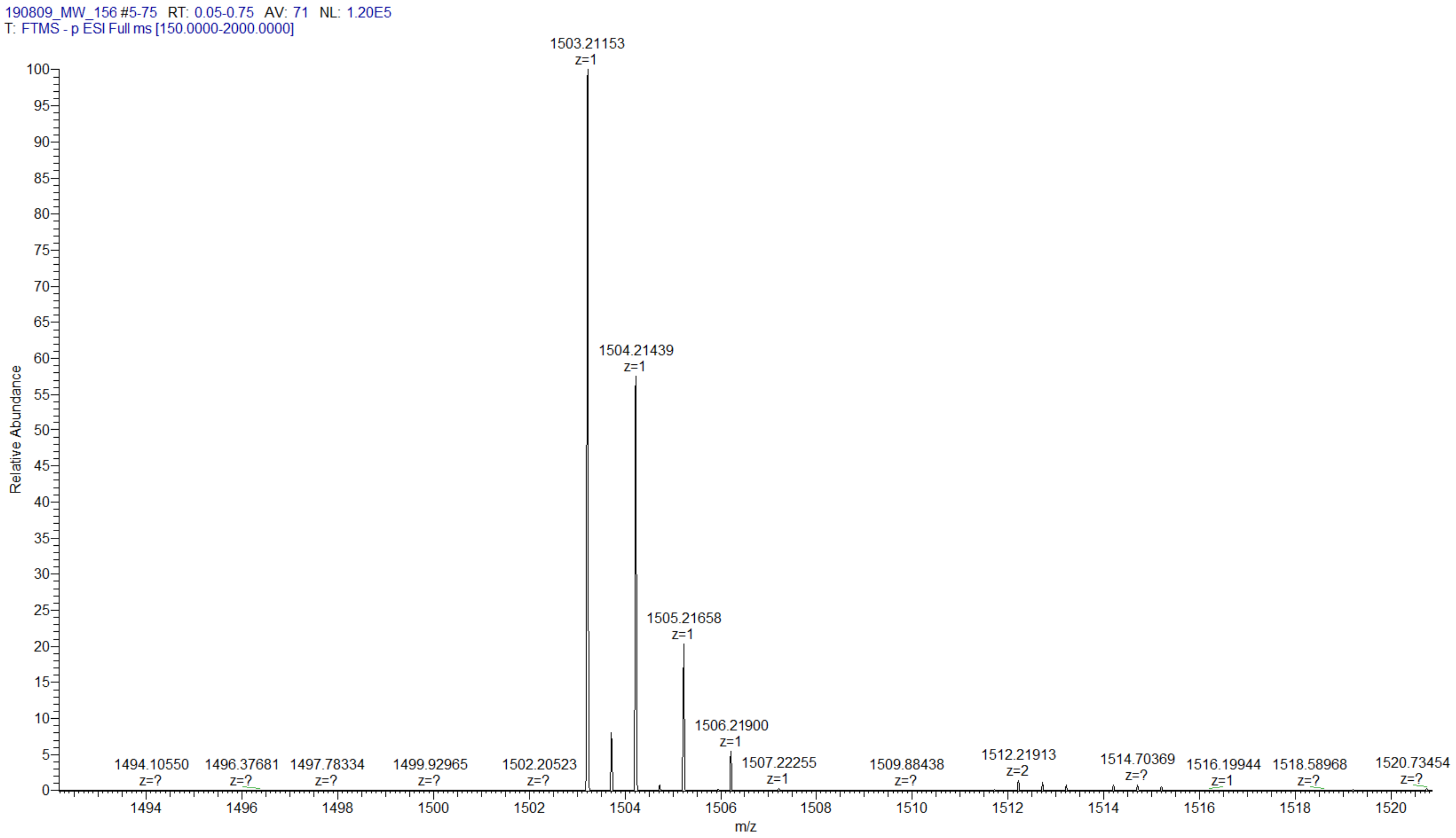

$m^7Gppp(m^6A_m)pG_mpG$

Chemical structure

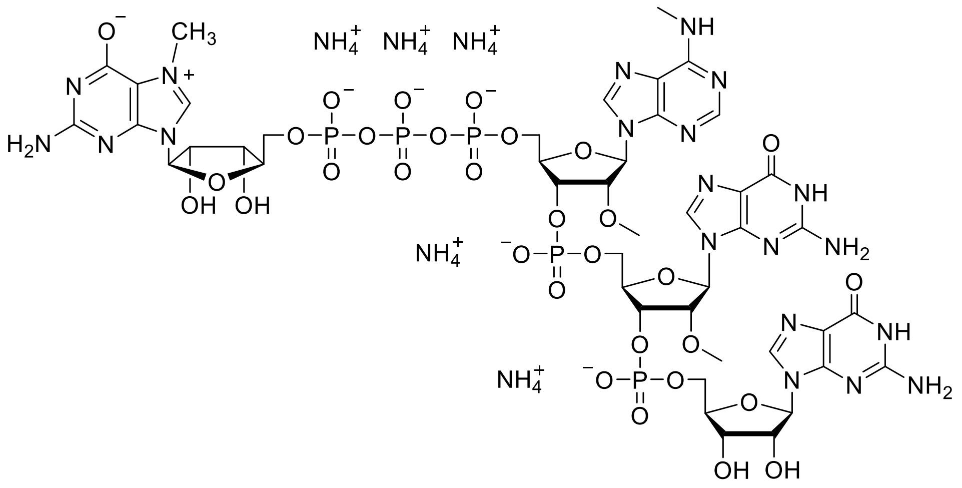

RP HPLC

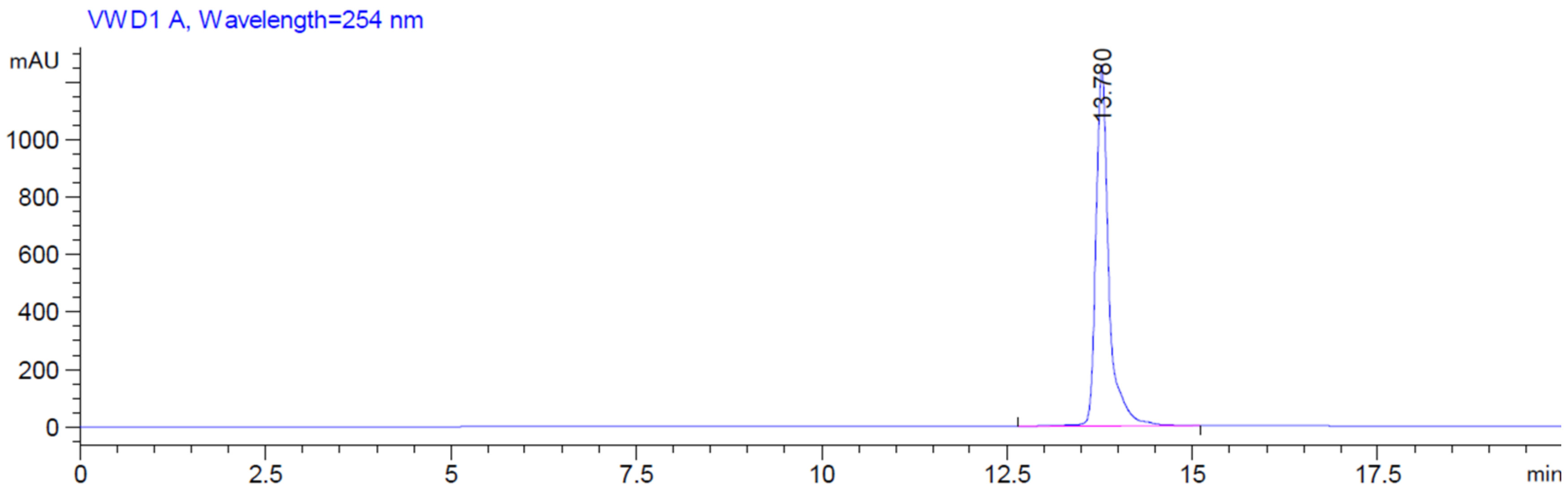

HR ESI(-) MS

Calc. [M-H]$^-$ $C_{44}H_{58}N_{20}O_{31}P_5^-$ 1517.22704)

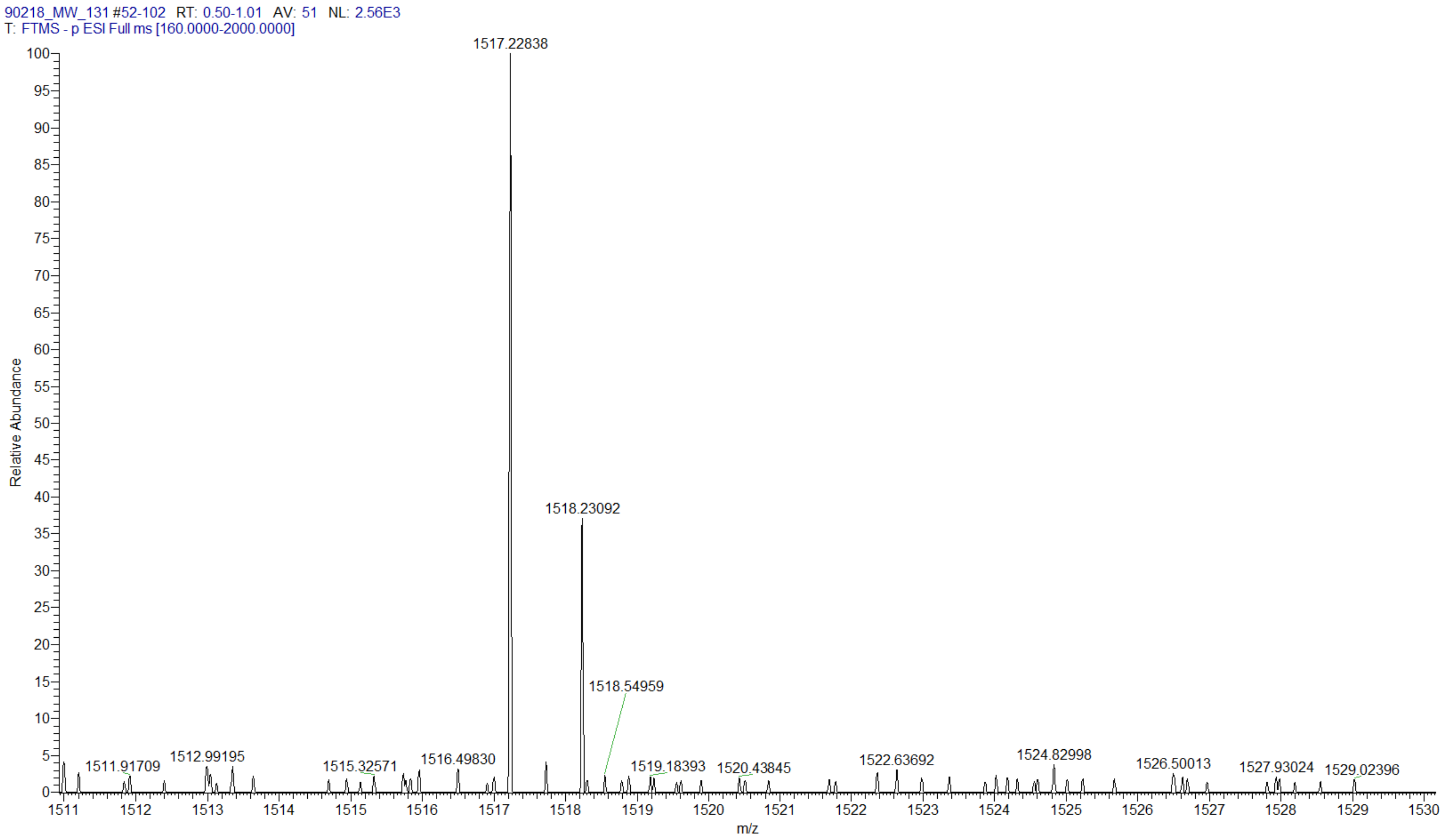

m^{7}GpppApG$_m$pG

Chemical structure

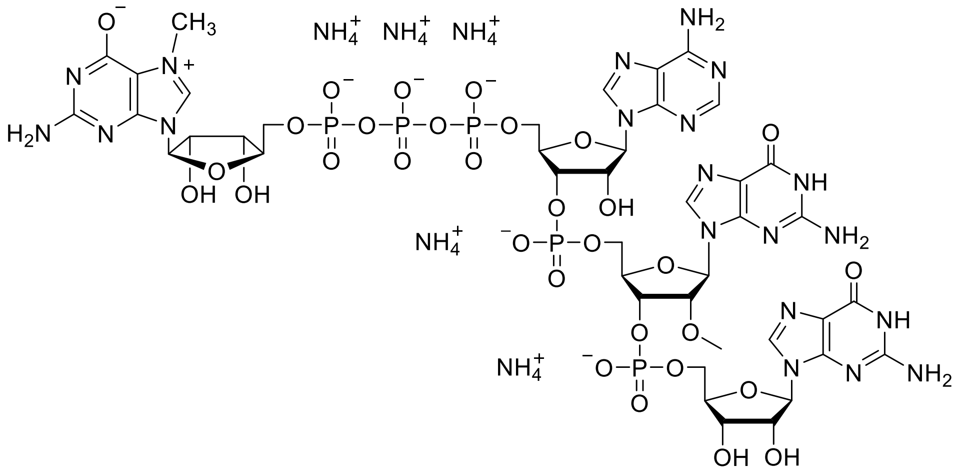

RP HPLC

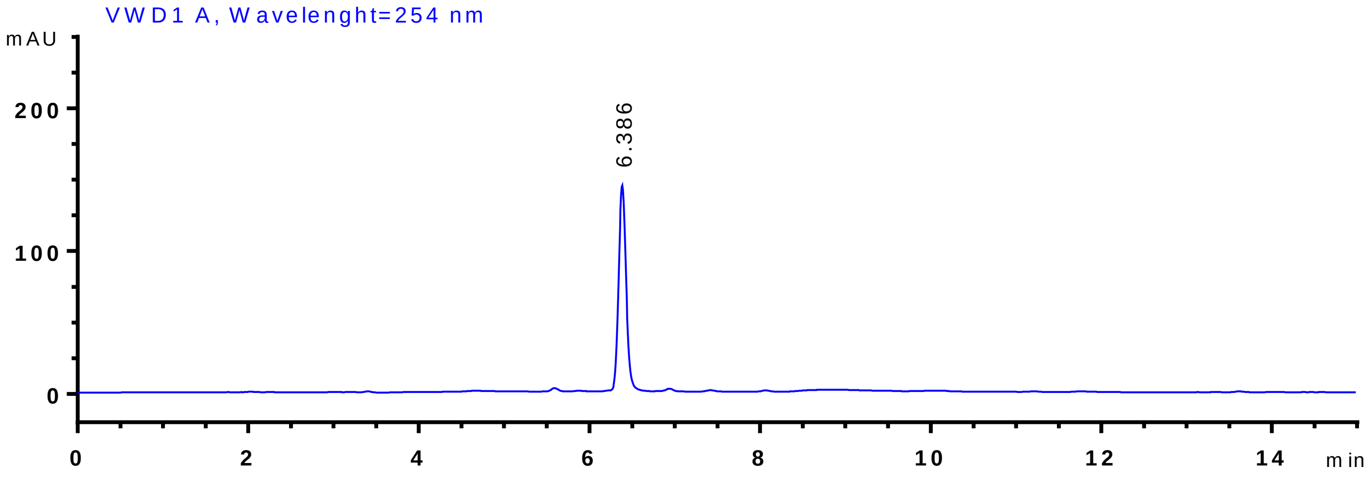

HR ESI(-) MS

Calc. $[M-H]^-$ $C_{42}H_{54}N_{20}O_{31}P_5^-$ 1489.19574)

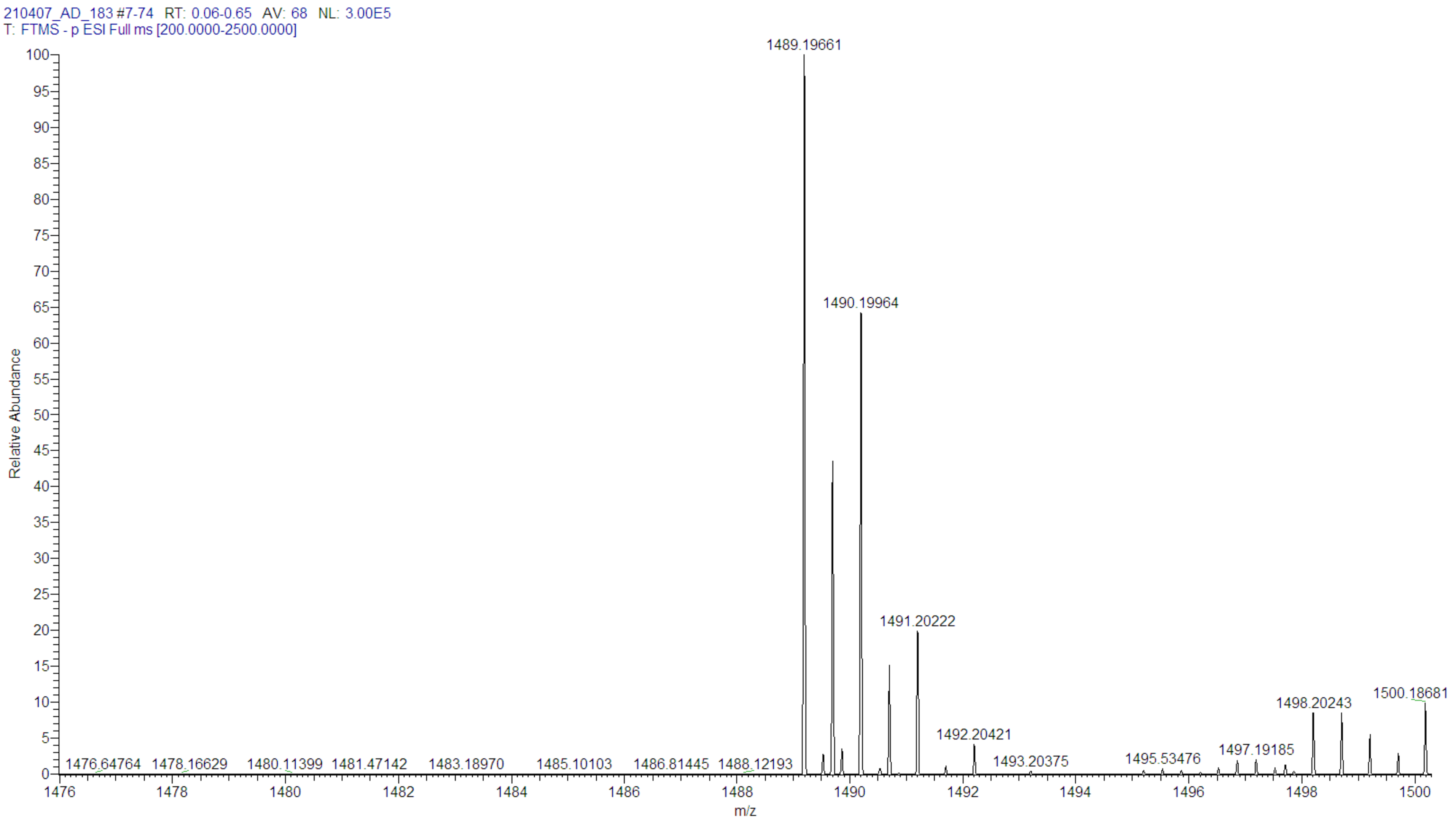

m^{7}Gppp(m^{6}A)pG$_m$pG

Chemical structure

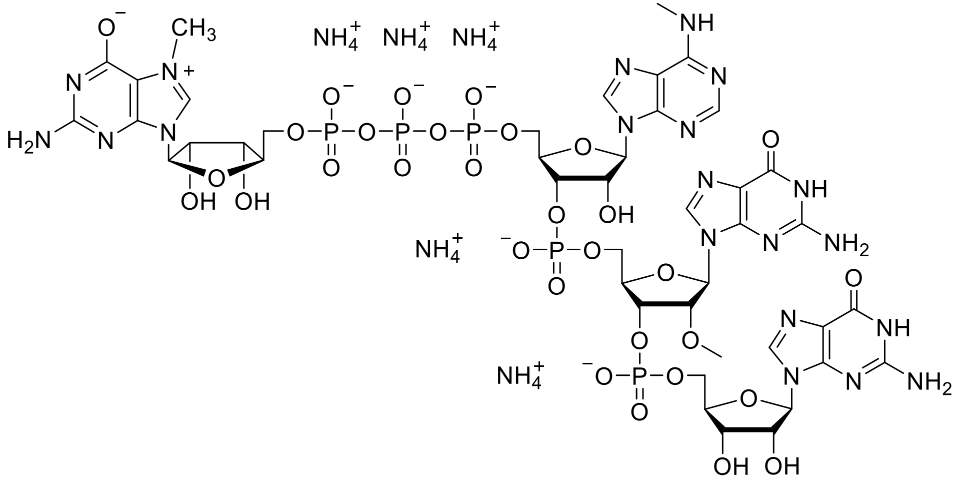

RP HPLC

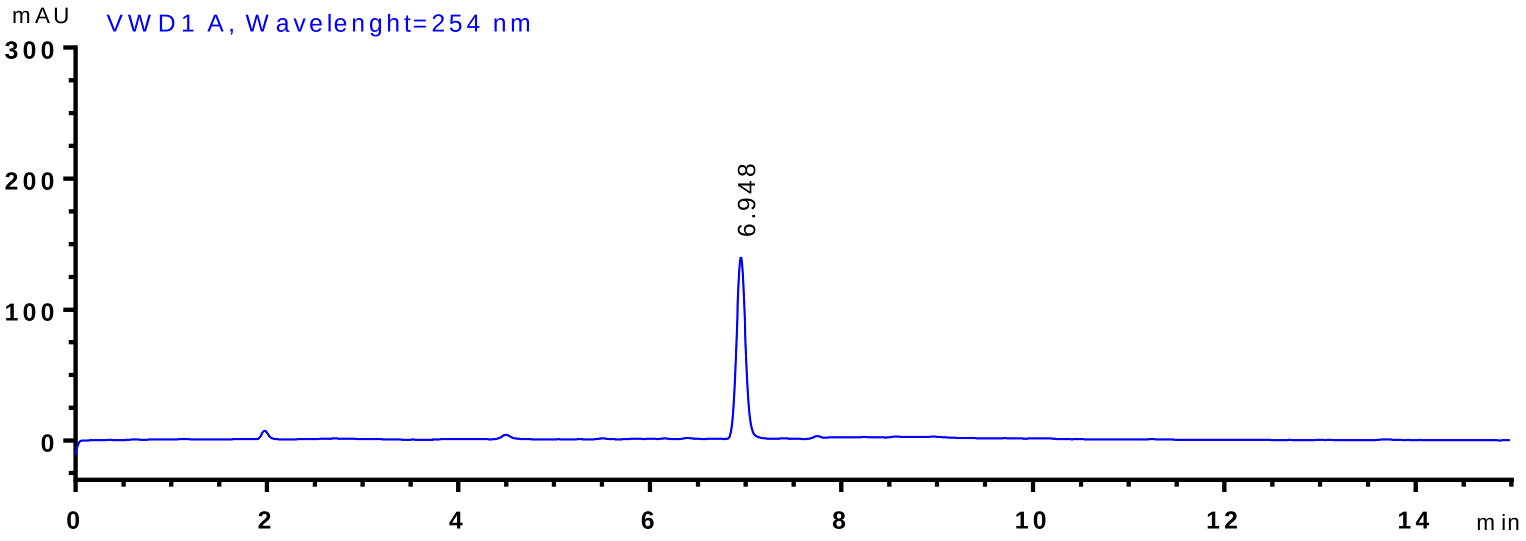

HR ESI(-) MS

Calc. [M-H]$^-$ $C_{43}H_{56}N_{20}O_{31}P_5^-$ 1503.21139)

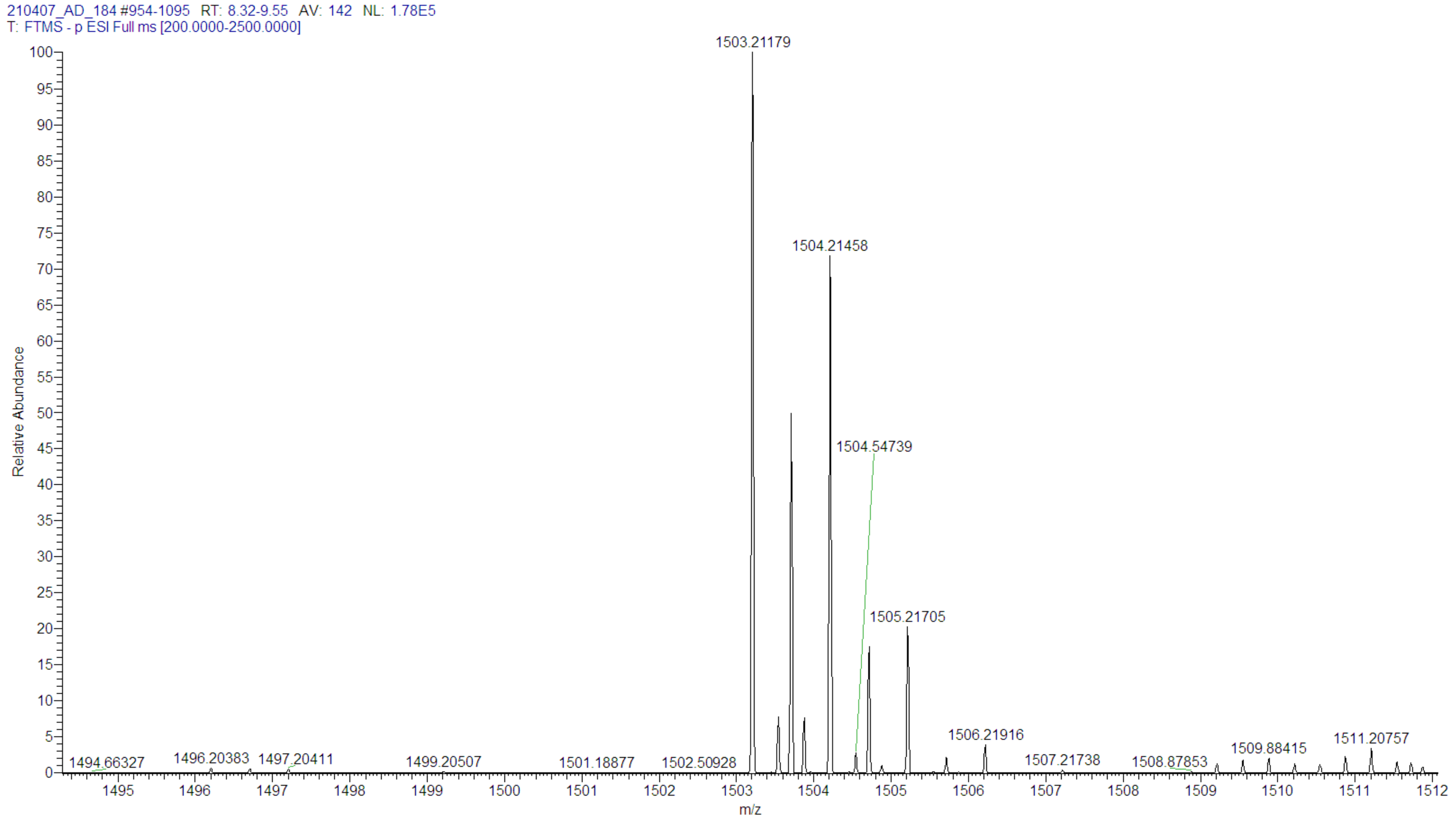

References

1. Tyanova, S., Temu, T., Sinitcyn, P., Carlson, A., Hein, M.Y., Geiger, T., Mann, M. and Cox, J. (2016) The Perseus computational platform for comprehensive analysis of (prote)omics data. *Nat Methods*, **13**, 731-740.
2. Coleman, T., Wang, G. and Huang, F. (2004) Superior 5 ' homogeneity of RNA from ATP-initiated transcription under the T7 phi 2.5 promoter. *Nucleic Acids Research*, **32**.
